# Better Use of Data to improve parent Satisfaction (BUDS): protocol for a prospective before-and-after pilot study employing mixed methods to improve parent experience of neonatal care

**DOI:** 10.1136/bmjpo-2019-000515

**Published:** 2019-06-25

**Authors:** Susanna Sakonidou, Izabela Andrzejewska, Sophia Kotzamanis, Wendy Carnegie, Mable Nakubulwa, Thomas Woodcock, Neena Modi, Derek Bell, Chris Gale, BUDS Project Steering Group, Chris Gale

**Affiliations:** 1 Neonatal Medicine, Imperial College London, London, UK; 2 Parent Representative, Chelsea and Westminster Hospital, London, UK; 3 NIHR CLAHRC for Northwest London, London, UK

**Keywords:** neonatology, patient perspective, qualitative research

## Abstract

**Introduction:**

Having a baby that requires neonatal care is stressful and traumatic. Parents often report dissatisfaction with communication of clinical information. In the UK neonatal care data are recorded daily using electronic patient record systems (EPR), from which deidentified data form the National Neonatal Research Database (NNRD). We aim to evaluate the impact of sharing neonatal EPR data with parents, on parent-reported satisfaction, parent–staff interactions, staff workload and data completeness.

**Methods:**

A prospective, before-and-after, mixed-method study. Participants are parents of inpatient babies (maximum 90) and staff in a tertiary neonatal intensive care unit, London, UK. The intervention was developed by former neonatal parents, neonatologists and neonatal nurses: a communication tool for parents comprising individualised, written, daily infant updates for parents, derived from EPR data. The intervention will be provided to parents over 6 weeks. Plan-Do-Study-Act cycles will inform the tool’s iterative development and improvement. The tool’s impact will be measured using a validated parent survey, staff survey, data completeness measures and interviews.

**Analysis:**

Primary outcome: parent satisfaction ‘with communication of clinical information and involvement in care’. Secondary outcomes: parent–staff interactions, staff workload, data completeness. Baseline survey data will be obtained from clinical service evaluation preceding the intervention. Baseline data completeness will be derived from the NNRD. During the intervention, surveys will be administered biweekly and data completeness assessed daily. We will analyse outcomes using run charts and partially paired statistical tests. Parent and staff interviews will explore information exchange and the communication tool’s impact.

**Discussion:**

This study will evaluate the impact of a parent co-designed intervention on communication with parents in neonatal care and the completeness of routinely recorded electronic clinical data. Better use of routinely recorded clinical data provides the opportunity to improve parent satisfaction and increase the research utility of such data, benefiting clinical care.

**Ethics and dissemination:**

Reviewed and approved by the West Midlands—South Birmingham REC (18/WM/0175).

**Registration number:**

ISRCTN62718241.

**What is known about the subject**

- Neonatal care significantly affects parents’ mental health, infant development and outcomes.
- Parent satisfaction is increasingly used as a parent experience measure and is commonly assessed using invalidated questionnaires.
- Interventions aimed at improving parent satisfaction traditionally do not involve parents in their design.

**What this study hopes to add**

- Interventions co-designed with parents receiving National Health Service (NHS) neonatal care have the potential to improve parent satisfaction.
- Using a validated parent satisfaction questionnaire for outcome measurement adds to the validity of results and enables comparison with other interventions.
- Implementing an intervention using routinely recorded infant data has the potential to make it sustainable and generalisable across NHS neonatal care.

## Introduction

One in eight UK newborn babies will require hospital care on a neonatal unit.^1^ This can be traumatic for parents, who often report it is difficult to obtain clinical information from staff^2^ and feel excluded from their baby’s care. Anxiety, depression and post-traumatic stress disorder symptoms have been reported in as high as 35% of parents following neonatal care^3 4^ and this stress has been shown to interfere with parent–child attachment and bonding.^5^ Improving parent experience in neonatal care can reduce parent stress, potentially improving parent–child bonding^5^ and infant outcomes.^6^ Parent satisfaction is inversely related to stress^7^ and is increasingly used as a parent experience measure and service quality indicator to inform high quality neonatal care.^8^

The latest national UK survey of parents’ neonatal experiences found that ‘parent satisfaction with receiving clinical information’ and ‘feeling involved in babies’ care’ were among the lowest scoring of all areas evaluated.^2^ Improving the quality and quantity of communication between parents and neonatal staff benefits patient care, promotes positive parent–child interaction and improves wider family well-being and satisfaction.^9 10^ There is evidence that parent satisfaction can be improved through simple interventions, such as providing written information to parents,^11 12^ however, previous interventions required additional work from staff to collect, record, collate and provide information to parents, limiting their sustainability and generalisability. Further limitations of previous studies include the failure to involve parents in study development and the use of invalidated questionnaires to measure the impact of interventions.^13^

The aim of this study is to use existing, routinely recorded data, from existing neonatal electronic patient record systems (EPR) to implement, further co-develop and evaluate a neonatal communication tool with parents. In the UK a defined set of data items are extracted from neonatal EPR systems to form the National Neonatal Research Database (NNRD).^14^ This holds information on all babies admitted for NHS neonatal care and is used for audit, benchmarking and research, but is currently not routinely shared with parents. The completeness of data in the NNRD is high^14^ but has the potential to further improve. There is a lack of evidence for interventions to improve the completeness of routinely collected data in neonatal care and more widely.

We will evaluate the impact of sharing routinely recorded infant information with parents, on parent satisfaction with ‘communication of clinical information and involvement in care’ using a validated questionnaire, and on the completeness of NNRD data extracted from neonatal EPR systems. We hypothesise that sharing individualised, daily EPR data with parents will lead to higher reported parent satisfaction and data completeness.

## Methods and analysis

### Parent and public involvement and co-development of the communication tool

This study was conceived in response to the clinical need identified by parents with neonatal care experience; a partnership including families with experience of preterm birth identified ‘what emotional and practical support improves attachment and bonding, and does the provision of such support improve outcomes for premature babies and their families?’ as a top 10 research priority.^15^

Parents of babies previously requiring NHS neonatal care were directly engaged in the study design. During the preparatory phase, the shared aim of the study, proposed intervention and preliminary qualitative work were informed by parent focus groups. A study steering group for the Better Use of Data to improve parent Satisfaction (BUDS) project was formed, including neonatologists, a neonatal nurse, parent participants, representation from Bliss (the national charity for babies born premature or sick) and the NIHR CLAHRC NWL improvement science team. A project-planning meeting was held to identify the problem with parent satisfaction, set the project’s aim to improve it and discuss ways to achieve that. Potential techniques were identified by parents for parents, one of which was a communication tool to share daily written infant information with parents. Parents co-designed the communication tool and validated a parent satisfaction survey for use in this study through focus groups and cognitive testing interviews. Parents will continue to be involved, as an advisory group, throughout the study in line with the 4Pi National Involvement Standards for effective co-development.^16 17^

Further iterative development of the communication tool, involving parents, will continue during evaluation using improvement science methodology (discussed below). Our parent representative will be involved in drafting all study output reports and will be encouraged and supported to present our findings at conferences.

Once the trial has been published, we will co-develop parent-centred study reports with parents from our steering group and Bliss, to be disseminated by Bliss and via social media. Study participants will be informed of the results through the study’s website and via email in a study newsletter.

### Communication tool

We previously identified the information parents report as important when their baby is on a neonatal unit through qualitative work with ex-neonatal parents. This information was matched to the routinely recorded neonatal daily EPR data items and we developed parent-friendly explanations of these EPR data items with parents. We co-designed a communication tool template for parents called ‘*My Baby’s Summary Report*’; this comprised a printed sheet of paper including individualised, daily infant updates for parents derived from EPR data, in parent-centred language.

### Design

**Figure 1:**
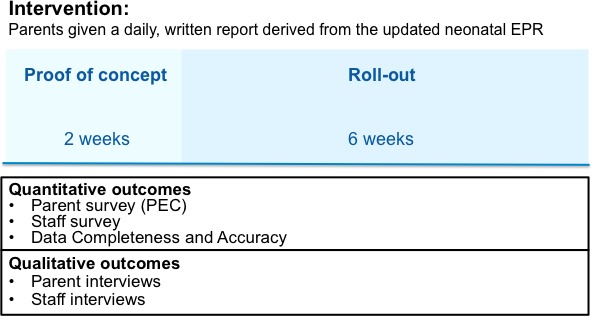
Study design overview. EPR, electronic patient record systems; PEC, parents’ experiences of communication in neonatal care.

A prospective before-and-after study employing mixed methods. The intervention (the communication tool) will be implemented in stages across the three intensity unit areas of the Chelsea and Westminster hospital neonatal intensive care unit (from low to high) over an 8-week period; a 2-week proof of concept phase will precede a 6-week roll-out phase (figure 1). An individualised *‘My Baby’s Summary Report’* will be administered to recruited parents in a sealed envelope every weekday. Quality improvement methodologies, including Plan-Do-Study-Act (PDSA) cycles^18–20^ and run-charts^21^ will be used to implement and further improve the communication tool. The content and the exact timing of the implementation in relation to the study will be fully described to avoid other concurrent parent-centred interventions influencing our study results.

### Participants

All eligible parents and neonatal staff members will be approached. Written informed consent will be obtained from all participants.

#### Parents

The lead researcher will recruit parents of babies that are inpatients on the Chelsea and Westminster hospital neonatal unit by directly approaching them on the unit during their baby’s stay. This neonatal unit in London, UK, provides tertiary level neonatal intensive care, including specialist surgical care, with 750 annual admissions and a 36-cot capacity.

- *Inclusion criteria:* We will start recruiting parents with babies in the lower intensity part of the unit for 4 weeks (Special Care Baby Unit- SCBU), who are medically stable and not acutely unwell. We will additionally recruit parents in the High Dependency Unit (HDU). area for 2 weeks and parents from the Intensive Care Unit (ICU) area for the final 2 weeks.
- *Exclusion criteria:* Parents that cannot speak and/or read English will be excluded as the study’s information documents and communication tool are in English. Parents younger than 16 years of age will be excluded.

Recruited parents will be asked to complete a demographic form including their age, gender, ethnic group, infant’s gestational age and infant’s length of stay on the neonatal unit.

#### Neonatal staff members

The lead researcher, in her role as part of the clinical team, will recruit staff members (nurses and doctors) by approaching them on the neonatal unit. Staff of all grades/seniority will be included.

### Outcome measures

#### Primary outcome

The primary outcome is parent satisfaction ‘with communication of clinical information and involvement in care’ and will be measured by a self-administered *Parents’ Experiences of Communication in neonatal care* survey (PEC). The PEC survey is an adaptation of the original UK national Neonatal survey 2014 carried out by the Picker Institute (see online supplementary file 3). We have completed qualitative cognitive survey testing with parents on the Chelsea and Westminster neonatal unit and are currently conducting a survey validation study, using preliminary survey data. The PEC study will be published once validation analysis is completed. The survey contains 35 questions (Likert scale and free text). There are five sections: staff on the neonatal unit (nine questions); information and support for parents (20 questions, six of which specific to *My Baby’s Summary Report*); your involvement in your baby’s care (four questions); leaving the neonatal unit (one question); your comments (one question).

10.1136/bmjpo-2019-000515.supp3 Supplementary data

#### Secondary outcomes

- Parent–staff interactions: Assessed by the PEC survey.
- Staff workload: Staff members will be asked to complete a three-question Staff Survey:

1. In the last 24 hours, have you updated parents during a ward round? Y/N
2. In the last 24 hours, how many times have you spoken to parents face-to-face about their baby (outside ward round times)?
3. In the last 24 hours, how many times have you spoken to parents over the phone?

- EPR data completeness and accuracy (expressed in %): The lead researcher will compare EPR-recorded data to handwritten clinical documentation to produce a daily measure of data completeness.
- Qualitative evaluations: The lead researcher will conduct one-on-one in-depth interviews with parents (30 min) and staff members (10 min). Interviews will explore the parent experience of receiving information updates in neonatal care and feeling involved in their baby’s care (see online supplementary file 1). For staff, interviews will explore their experience of giving parents information updates and perceptions of how updating parents affects their workload (see online supplementary file 2). Interviews will be recorded and anonymously transcribed.

10.1136/bmjpo-2019-000515.supp1 Supplementary data

10.1136/bmjpo-2019-000515.supp2 Supplementary data

### Implementation of the intervention

#### Stage 1: ‘Proof of concept’ (2 weeks—SCBU, lowest intensity area)

We will recruit three parents from SCBU and administer an individualised *‘My Baby’s Summary Report’* to them in a sealed envelope every weekday, over 2 weeks. We will administer PEC and staff surveys at baseline, weekly and at the end, and we will assess EPR data completeness and accuracy daily.

Data analysis will be aimed at improving the *‘My Baby’s Summary Report’* template and the piloting process. We will use PDSA cycles^18–20^ to analyse parental free-text feedback from PEC surveys and adapt the report’s template, creating the final template version for use in Stage 2 ‘Roll-out’. We will plot the parent and staff survey data on run charts, a standard method of quality control and a useful tool in quality improvement that enables one to monitor the performance of a process over time.^21^

#### Stage 2: ‘Roll out’ (6 weeks—SCBU 2 weeks—HDU 2 weeks—ITU 2 weeks)

**Figure 2:**
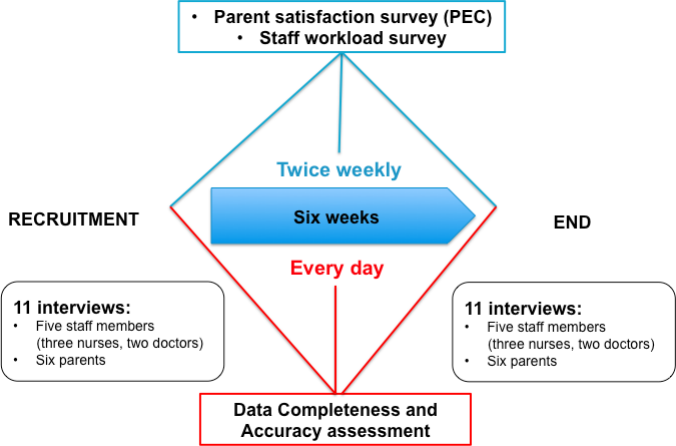
Roll-out stage. PEC, parents’ experiences of communication in neonatal care.

We will recruit a maximum of 90 parents, administer *‘My baby’s Summary Report’* every weekday and conduct a qualitative and quantitative evaluation over 6 weeks, while also using quality improvement methodology to further improve the communication tool (figure 2).

### Analysis

#### Baseline measurement

Baseline measures for parent satisfaction and staff workload will be obtained from on-going evaluations undertaken as part of clinical service evaluation on the neonatal unit; these evaluations use the PEC survey and staff survey. Data from PEC and staff surveys from a 6-week period before the start of the trial will be used to generate baseline median values for each PEC survey item and staff survey questions using run charts (21).

#### Quantitative outcomes

For parent and staff outcomes, we will

1. Demonstrate how the outcomes change over time: For continuous variables derived from the PEC’s Likert scale questions we will use run charts (21). For categorical variables we will conduct a comparison of response distributions precommunication and postcommunication tool implementation.
2. Evaluate the communication tool’s specific impact on outcomes: Using partially paired parent data, we will compare the difference in the outcomes of parent satisfaction, parent–staff interactions and staff workload from baseline to repeat measures, between the baseline measurement period (previous service evaluation assessment) and the trial intervention period. NNRD data completeness will be expressed in percentages, compared with 3-month historical NNRD baseline measures and analysed using run charts (21).

#### Qualitative evaluation

We will analyse parent and staff interview transcripts, identify and code themes using a Grounded Theory Approach.^22^ We will analyse results by theme and produce a summary report of how parents and staff interact on the neonatal unit, how information exchange takes place, how parents and staff feel about it and would like it to happen (precommunication and postcommunication tool usage). We will also produce a summary report of parents’ and staff impressions of the communication tool.

## Discussion

This study will evaluate the impact of a parent co-designed intervention to improve communication in neonatal care. The strengths of this study include the active involvement of parents throughout, from conception and design through to dissemination, and that parents with infants receiving neonatal care are prospectively co-designing the intervention. A further strength is that the intervention uses routinely recorded NHS neonatal infant data, without incurring any extra work to staff. In contrast to previous studies, we are using a parent satisfaction questionnaire validated with parents of babies receiving NHS neonatal care to evaluate our intervention. These parent survey data will also be used to further develop and improve the intervention by analysing parent free-text feedback and conducting PDSA improvement cycles. Finally, because parent satisfaction is not a static measure, but can change over time (even in the absence of any interventions), we are comparing outcome measures post intervention to routinely recorded longitudinal baseline measures, rather than single preintervention and postintervention measures. Limitations of our study include involvement only of parents that can speak and understand written English. This is because parents need to be involved in the co-design: our self-administered survey is only available in English and this process cannot easily be facilitated using interpreters. Another limitation is recruitment, which will be done using a non-randomised design and therefore is susceptible to bias. Randomisation of an intervention such as that being developed and evaluated in this study is inherently difficult to blind and has a high risk of contamination; parents in neonatal care regularly converse and exchange ideas. In order to avoid specific parent characteristics influencing the study’s outcomes, we will measure and correct for parent demographic details. We will also describe any other relevant parent-centred co-interventions taking place during our study period. Finally, as with any survey-based study we may have significant drop off. Survey responses are subjective and can be influenced by many factors, including parental anxiety, depressive symptoms, severity and changes in infants’ condition, which we are not measuring, as our study is a small-scale pilot. A cluster RCT may be the optimal methodological approach to definitively evaluate an intervention such as this; our study will inform such a future cluster RCT to further evaluate the impact of sharing routinely recorded infant information in neonatal care.

On completion of this study we will have also identified the final template of EPR data items that parents would like to receive on a daily basis in neonatal care. The incorporation of these findings into a digital medium, such as a mobile phone application and/or website, would allow for better efficiency and wider generalisability of the study findings.
