# Supplementary material for: Better Use of Data to improve parent Satisfaction (BUDS): protocol for a prospective before-and-after pilot study employing mixed methods to improve parent experience of neonatal care
Source: BMJ Paediatr Open. 2019 Jun 25;3(1):e000515. doi: 10.1136/bmjpo-2019-000515 (PMC6598559; doi:10.1136/bmjpo-2019-000515)
Supplement: Supplementary data [file bmjpo-2019-000515supp003.pdf]

Parents' experiences of neonatal care: Findings from Neonatal Survey 2014

Executive Summary

March 2015

Sarah-Ann Burger

www.pickereurope.org

Picker Institute Europe

Picker Institute Europe is an international charity dedicated to ensuring the highest quality health and social care for all, always. We are here to:

- Influence policy and practice so that health and social care systems are always centred around people's needs and preferences.
- Inspire the delivery of the highest quality care, developing tools and services which enable all experiences to be better understood.
- Empower those working in health and social care to improve experiences by effectively measuring, and acting upon, people's feedback.

© Picker Institute Europe 2015

Published by and available from:
Picker Institute Europe
Buxton Court
3 West Way
Oxford, OX2 0JB
England

Tel: 01865 208100
Fax: 01865 208101
Email: Info@PickerEurope.ac.uk
Website: www.pickereurope.org

Registered Charity in England and Wales: 1081688

Registered Charity in Scotland: SC045048

Company Limited by Registered Guarantee No 3908160

Picker Institute Europe has UKAS accredited certification for ISO20252: 2012 (GB08/74322) and ISO27001:2005 (GB10/80275). Picker Institute Europe is registered under the Data Protection Act 1998 (Z4942556). This research conforms to the Market Research Society's Code of Practice.

Contact for Further Information

For further information about this report, please contact: Sarah-Ann Burger or Amy Tallett at the Picker Institute on 01865 208100 or email Neonatal.Survey@PickerEurope.ac.uk

Introduction

Assessing parents' experiences of neonatal services is crucial for an understanding of how the quality of care can be improved. By ensuring that organisations carry out the survey in a consistent and systematic way, it is possible to build up a national picture of parents' experiences of neonatal care and, with caution, to compare the performance of different organisations, monitor change over time, and identify variations between different groups of respondents. Such findings can be used at a local level to drive improvements in overall experiences of neonatal services.

This document summarises the findings from the Neonatal Survey 2014 carried out by the Picker Institute in collaboration with Bliss (the charity "for babies born too soon, too small, too sick") and NHS England who kindly part-funded the survey. The purpose of the survey was to understand the experience of parents whose baby received care in a neonatal unit, to assist units in understanding where they are doing well and what could be improved.

The survey involved 88 hospital neonatal units from 72 NHS trusts in England (including special care baby units, local neonatal units and neonatal intensive care units), in addition to the neonatal services at Jersey General Hospital. Participating units fell into 13 different neonatal networks across England.

The survey fieldwork was conducted in two waves in order to obtain a sufficiently sized sample to allow the results to be presented at unit-level:

- Wave 1: sample of babies discharged from neonatal care between October 2013 and March 2014
- Wave 2: sample of babies discharged from neonatal care between April and September 2014

For each neonatal unit, a sample of up to 100 parents per wave whose babies were consecutively discharged alive[1] were sent a questionnaire to complete at home. Questionnaires were mailed to parents between May and December 2014 and up to two reminders were sent to non-responders.

Parents aged 16 and over were eligible for the survey if their baby had received neonatal care for 24 hours or longer and had been discharged home during the specified sampling periods.

A total of 15,944 eligible parents were sent a questionnaire. Responses were received from 6000 parents, a response rate of 37.6%.

[1] If a unit had fewer than 100 babies discharged in the sampling period then all eligible parents were included in the survey.

Key Findings

This section provides an overview of the key findings, focusing on those areas where parents reported their most positive and least positive experiences of neonatal care. Those questions showing the greatest variation across neonatal networks in parents' experiences are also highlighted.

Positive Parent Experience

The results presented here are based on the scores calculated for each question in the survey. For an explanation of how scores are calculated and should be interpreted, refer to Section 1 of the main report.

Table 1. Question areas where parents reported the most positive experience

Question	Score
**G3** Were you offered overnight accommodation with your baby at the hospital before they left the neonatal unit?	95
**E5** Were you able to visit your baby on the unit as much as you wanted to?	95
**C13** Overall, did you have confidence and trust in the staff caring for your baby?	92
**G6** How likely are you to recommend this neonatal unit to friends and family, if their baby needed similar care or treatment?	91
**B4** After your baby was admitted to the neonatal unit, were you able to see your baby as soon as you wanted?	91
**C5** Were you told which nurse was responsible for your baby's care each day s/he was in the neonatal unit?	90
**C12** Did staff refer to your baby by his/her first name?	90
**D4** Were you told about any changes in your baby's condition or care?	89
**D7** Overall, did staff help you feel confident in caring for your baby?	89
**D1** Were you involved as much as you wanted in the day-to-day care of your baby, such as nappy changing and feeding?	89

Key areas for improvement

Table 2. Question areas where parents reported the least positive experience

Question	Score
**F4** Were you offered emotional support or counselling services from neonatal unit staff?	57
**B3** After you gave birth, were you ever cared for in the same ward as mothers who had their baby with them?	55
**A2** Before your baby was born (i.e. during pregnancy or labour), did a member of staff from the neonatal unit talk to you about what to expect after the birth?	54
**F2** Were you given enough written information to help you understand your baby's condition and treatment?	53
**F6** Did staff give you any information about parent support groups, such as Bliss or other local groups?	53
**F5** Were you given enough information about help you could get with expenses related to your baby's stay in the neonatal unit (such as travelling/parking expenses, hardship fund or food vouchers?	41

Variations across networks

Some differences in parental experiences by neonatal network were revealed. Overall, Greater Manchester and Trent Perinatal networks had the highest proportion of parents responding positively to most questions compared to other networks, whereas the London network had the greatest room for improvement.

Questions with the largest variation of positive feedback from parents across networks include:

- B2: After you gave birth, were you offered a photograph of your baby?
- D5: When a ward round was taking place, were you allowed to be present when your baby was being discussed?
- G3: Were you offered overnight accommodation with your baby at the hospital before they left the neonatal unit?
- A2: Before your baby was born (i.e. during pregnancy or labour), did a member of staff from the neonatal unit talk to you about what to expect after the birth?
- D6: Where possible, did staff arrange your baby’s care (such as weighing, bathing) to fit in with your usual visiting times?
- E4: If you wanted to stay overnight to be close to your baby, did the hospital offer you accommodation?
- F5: Were you given enough information about help you could get with expenses related to your baby's stay in the neonatal unit (such as travelling/parking expenses, hardship fund or food vouchers?
- B3: After you gave birth, were you ever cared for in the same ward as mothers who had their baby with them?
- C12: Did staff refer to your baby by his/her first name?

For a detailed breakdown of results by network, please refer to Section 4 of the main report.

Individual unit reports

Each neonatal unit that participated in the 2014 Neonatal Survey will receive an individual report of their findings to use locally to drive improvement. These reports include:

- Overall score for each question
- Benchmarking against units of the same type (i.e. LNU, SCBU, NICU)
- Benchmarking against the overall average score
- Benchmarking against units of the same network
- Overall frequency tables
- Freetext comments from parents

Conclusion and Next Steps

The neonatal survey 2014 results indicate areas of good performance in addition to highlighting where there is room for improvement. Communicating results and priorities for service improvement across organisations and in local areas, will be key to ensuring that changes are implemented successfully. Patients and staff should be involved in developing action plans and any resulting quality improvement activities. Therefore, the Picker Institute are hosting two regional workshops to assist units with interpreting their results and identifying areas for improvement.

Parents’ experiences of neonatal care: Findings from Neonatal Survey 2014

Final Report

March 2015

Sarah-Ann Burger

Senior Project Manager, Health Experiences Team

www.pickereurope.org

Picker Institute Europe

Picker Institute Europe is an international charity dedicated to ensuring the highest quality health and social care for all, always. We are here to:

- Influence policy and practice so that health and social care systems are always centred around people's needs and preferences.
- Inspire the delivery of the highest quality care, developing tools and services which enable all experiences to be better understood.
- Empower those working in health and social care to improve experiences by effectively measuring, and acting upon, people's feedback.

© Picker Institute Europe 2015

Published by and available from:
Picker Institute Europe
Buxton Court
3 West Way
Oxford, OX2 0JB
England

Tel: 01865 208100
Fax: 01865 208101
Email: Info@PickerEurope.ac.uk
Website: www.pickereurope.org

Registered Charity in England and Wales: 1081688
Registered Charity in Scotland: SC045048
Company Limited by Registered Guarantee No 3908160

Picker Institute Europe has UKAS accredited certification for ISO20252: 2012 (GB08/74322) and ISO27001:2005 (GB10/80275). Picker Institute Europe is registered under the Data Protection Act 1998 (Z4942556). This research conforms to the Market Research Society's Code of Practice.

Contact for Further Information

For further information about this report, please contact: Sarah-Ann Burger or Amy Tallett

Email: Neonatal.Survey@PickerEurope.ac.uk

Tel: 01865 208 100

Contents

Section One 2
Introduction 2
Section Two 2
Survey Response 2
Section Three 2
Overall Results 2
Section Four 2
Overall Results by Network 2
Section Five 2
Overall Results by Unit Type 2
Section Six 2
Free Text Comments 2
Appendix A 2
Frequency Tables 2

Section One

Introduction

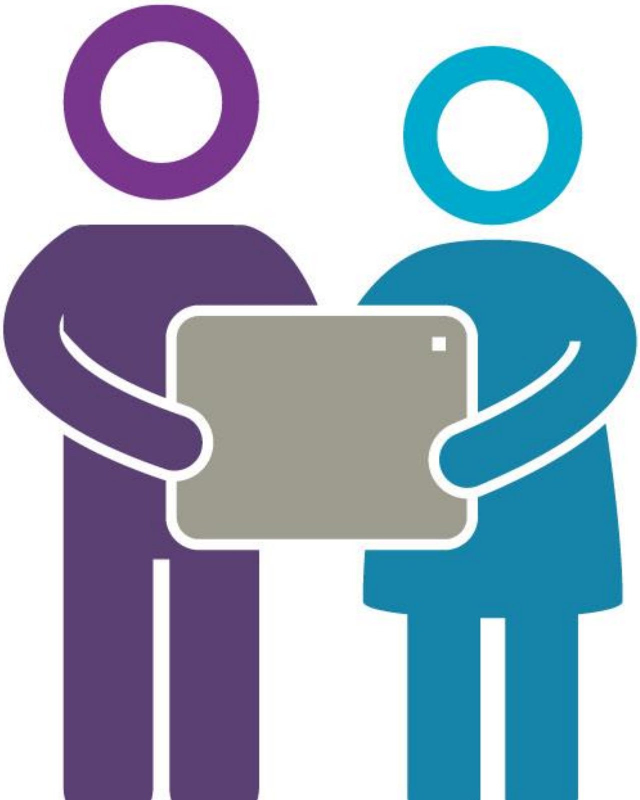

Background

Assessing parents' experiences of neonatal services is crucial for an understanding of how the quality of care can be improved. By ensuring that organisations carry out the survey in a consistent and systematic way, it is possible to build up a national picture of parents' experiences of neonatal care and, with caution, to compare the performance of different organisations, monitor change over time, and identify variations between different groups of respondents. Such findings can be used at a local level to drive improvements in overall experiences of neonatal services.

The results presented in this report are the overall results of all participating neonatal units in England from the Neonatal Survey 2014, carried out by the Picker Institute in collaboration with Bliss (the charity "for babies born too soon, too small, too sick") and NHS England who kindly part-funded the survey. The purpose of the survey was to understand the experience of parents whose baby received care in a neonatal unit, to assist units in understanding where they are doing well and what could be improved.

The Questionnaire

In 2010, Bliss (the charity "for babies born too soon, too small, too sick") approached researchers at the Picker Institute to develop a questionnaire, in conjunction with the neonatal networks, to examine parents' experiences of neonatal care. The survey was implemented in 2010-2011 with 125 English neonatal units, including special care baby units (SCBUs), local neonatal units (LNUs), and neonatal intensive care units (NICUs).

The survey was repeated in 2014 in order to:

- give NHS Trusts and neonatal units in England another opportunity to assess parental experiences of neonatal care and benchmark against other participating organisations using a standardised methodology;
- monitor performance and measure the impact of any quality improvement initiatives introduced since the last survey.

For the 2014 survey the Picker Institute, in collaboration with Bliss and NHS England, refined and updated the questionnaire following a consultation exercise with neonatal network leads. Some relatively minor survey changes were made to ensure that the questions were consistent with current service provision. The amended survey was cognitively tested using three rounds of cognitive interviews with 12 parents whose baby had recently received care in a neonatal unit. Cognitive testing involves interviews to go through the questionnaire with members of the target group to ensure that the survey questions are appropriate and relevant, and interpreted as intended, to maximise the validity of the data that they collect.

The updated 2014 survey of parents' experiences of neonatal care was offered to all NHS Trusts and units in England. The survey was promoted via communications from the Picker Institute, Bliss and NHS England. 72 NHS trusts (88 hospital neonatal units) in England signed up to the survey (including special care baby units, local neonatal units and neonatal intensive care units), in addition to the neonatal services at Jersey General Hospital.

©2015 Picker Institute Europe. All Rights Reserved.

Survey methodology

Picker Institute Europe coordinated the survey fieldwork, which was conducted in two waves in order to obtain a sufficiently sized sample to allow the results to be presented at unit-level:

- Wave 1: sample of babies discharged from neonatal care between October 2013 and March 2014
- Wave 2: sample of babies discharged from neonatal care between April and September 2014

For each unit, a sample of up to 100 parents whose babies were consecutively discharged alive from the neonatal unit were sent a questionnaire to complete at home[1]. Questionnaires were mailed to parents' between May and December 2014 and up to two reminders were sent to non-responders. Parents aged 16 and over were eligible for the survey if their baby had received neonatal care for 24 hours or longer and had been discharged home during the specified sampling periods.

Parents were sent a questionnaire, a covering letter from the trust, a multiple language sheet offering help with the survey, and a FREEPOST envelope. Parents wishing to complete the survey filled it in and returned it to the Picker Institute in the FREEPOST envelope. Non-responders were sent a reminder letter after 2-3 weeks, and another questionnaire and final reminder letter after a further 2-3 weeks.

The Picker Institute ran a Freephone helpline for patients who had any queries or concerns about the survey. This included links to LanguageLine with immediate access to interpreters in over 100 languages. Parents wishing to opt-out of the survey could do so by returning the questionnaire blank, or by calling the Freephone helpline. A total of 73 calls were received, which included 1 LanguageLine request.

[1] If a unit had fewer than 100 babies discharged in the sampling period then all eligible parents were included in the survey.

©2015 Picker Institute Europe. All Rights Reserved.

Interpreting this report

The results present the overall findings for all participating units, in addition to:

- breakdowns by network and,
- breakdowns by unit type (NICU, SCBU, LNU).

The percentage calculations throughout the report **exclude respondents that did not answer**. Full frequency tables are included at the end of the report in Appendix A, which display the number of missing responses for each question, along with details of who and how many people answered, and the proportion of patients that selected each response option. Please note that differences between networks, and unit type have not been tested for statistical significance.

The data have been 'standardised' by the age of respondents and by the gestational age of the baby at birth. The only exception to this approach is in section H on the demographics as it is more appropriate to present the real percentage figures rather than standardised data for these questions.

Scores

For findings presented in Section 3 'Overall Results' the individual responses to questions in the survey were converted into scores on a scale of 0 to 100. A score of 100 represents the best possible response. Therefore, the higher the score for each question, the better the units are performing. The overall score for each question is calculated as an average of the individual scores. The scores are not percentages, so a score of 80 does not mean that 80% of parents who experienced neonatal care have had a particular experience (e.g. ticked 'Yes' to a particular question), it means that the overall scores is 80 out of a maximum of 100. A 'scored' questionnaire showing the scores assigned to each question is available upon request.

Not all response options are able to be scored; many questions within the questionnaire include an option that cannot be used to evaluate the unit's performance. For example, if a respondent cannot remember or does not know the answer to a question, a score is not given. It is also not appropriate to score all questions within the questionnaire for benchmarking purposes. This is because not all questions assess the neonatal units in any way, or they may be 'filter questions' designed to filter out respondents to whom following questions do not apply. An example of a filter question would be A1 "Before your baby was born, did you know that they might need care in a neonatal unit?"

Graphs

In Section 4 and Section 5 of the report, findings are presented as graphs. These are arranged by questionnaire section. The graphs included in this report display percentages for each question, comparing networks and the national average (section 3), and by unit type and the national average (section 4). For questions that measure performance, the graphs use a range of red, amber and green to present the percentage of people who responded positively (green), less positively, (amber) or negatively (red) to a specific question.

©2015 Picker Institute Europe. All Rights Reserved.

Confidence intervals

As the survey was not of all parents who responded rather than all parents, the results may not be exactly the same as if everyone had responded. However, we can estimate the level of confidence we should have in the results by considering the confidence interval surrounding the results. The table below shows the level of confidence we would have for various numbers of respondents.

Number of respondents	Confidence Interval (+/-)
50	12.0%
100	8.5%
200	6.0%

Example: For a particular question, 200 patients responded, of which 25% answered ‘yes’.

From the table above, we can see that for 200 respondents the confidence interval would be +/- 6.0%. We would therefore estimate that the true results could be between 19.0% and 31.0%. However, if only 50 patients responded, and 25% answered ‘yes’, the confidence intervals would be +/- 12% so the true result could be between 13% and 37%.

Rounding of percentages

Note that throughout the report, partial percentages have been rounded to the nearest full number. For example 12.8% is rounded up to 13%, while 5.3% would be rounded down to 5%.

Overall average

The overall average is based on results from the 87 hospital neonatal units in England who participated in the survey. The overall average excludes Jersey General Hospital as they are a non-NHS provider.

Individual unit reports

Each neonatal unit that participated in the Neonatal Survey 2014 receives an individual report of their findings to use locally to drive improvement. The report aims to assist units to identify key areas for improvement from the perspective of parents. Results are presented in a few ways including:

- Overall score for each question
- Benchmarking against units of the same type (i.e. LNU, SCBU, or NICU)
- Benchmarking against the overall average score
- Benchmarking against units of the same network
- Overall frequency tables
- Freetext comments from parents

©2015 Picker Institute Europe. All Rights Reserved.

Section Two

Survey Response

Response rates and demographics

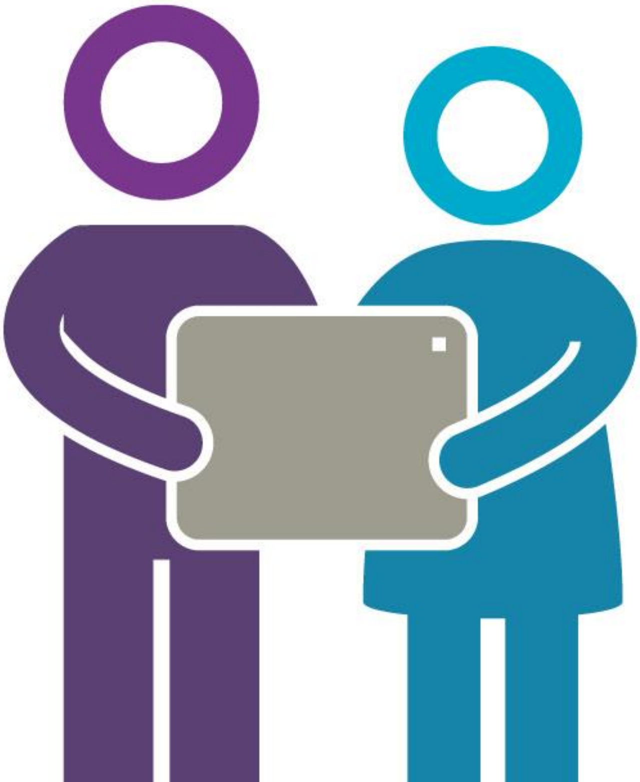

Response Rates

This section displays the response rate for each NHS neonatal unit that participated in the survey, as well as the overall response rate for each network.

Overall, responses were received from 6000 parents, a response rate of **37.6%.** Trent Perinatal Network received the highest response rate (46%, n=242), whereas South West Midlands Maternity and Newborn Network (31%, n=346) had the lowest response rate compared to other networks.

Table 1. Response rates by neonatal units and respective networks.

Network	Unit	Eligible Invited	Returned	Response Rate
**Cheshire and Merseyside Neonatal Network**	Arrowe Park Hospital	193	57	30%
	Whiston Hospital	148	42	28%
	Alder Hey Hospital	86	41	48%
	Liverpool Women's Hospital	296	90	30%
	Macclesfield District General Hospital	99	42	42%
	Countess Of Chester Hospital	196	82	42%
	Ormskirk & District General Hospital	130	54	42%
	Warrington Hospital	200	82	41%
	**Total**	**1348**	**490**	**36%**
**Greater Manchester Neonatal Network**	Wythenshawe Hospital	189	26	14%
	Royal Bolton Hospital	193	68	35%
	Royal Albert Edward Infirmary	124	48	39%
	St Mary's Hospital, Manchester	197	72	37%
	North Manchester General Hospital	194	76	39%
	Royal Oldham Hospital	198	82	41%
	**Total**	**1095**	**372**	**34%**
**Lancashire and South Cumbria Neonatal Network**	Royal Preston Hospital	198	68	34%
	**Total**	**198**	**68**	**34%**

©2015 Picker Institute Europe. All Rights Reserved.

Table 1. *continued*

Network	Unit	Eligible Invited	Returned	Response Rate
**London Neonatal Network**	The Royal London Hospital	193	20	10%
	Whipps Cross University Hospital	194	18	9%
	Newham General Hospital	194	17	9%
	The Royal Free Hospital	144	51	35%
	Kingston Hospital	196	89	45%
	Ealing Hospital	125	30	24%
	King George Hospital	178	53	30%
	Queen's Hospital, Romford	181	53	29%
	St Thomas' Hospital	199	87	44%
	University Hospital Lewisham	199	96	48%
	Queen Elizabeth Hospital Woolwich	142	47	33%
	St George's Hospital	192	88	46%
	Chelsea & Westminster Hospital	195	91	47%
	University College Hospital	199	76	38%
	Northwick Park Hospital	183	40	22%
	St Mary's Hospital	175	53	30%
	Queen Charlotte's And Chelsea Hospital	178	63	35%
	**Total**	**3067**	**972**	**32%**
**Midlands Central Newborn Network**	Queen's Hospital, Burton Upon Trent	91	49	54%
	University Hospital Coventry	196	78	40%
	George Eliot Hospital	181	61	34%
	Northampton General Hospital	182	72	40%
	Leicester Neonatal Service	196	83	42%
	**Total**	**846**	**343**	**41%**
**South East Coast Neonatal Network**	Frimley Park Hospital	199	106	53%
	Medway Maritime Hospital	197	75	38%
	St Peter's Hospital	185	82	44%
	East Surrey Hospital	197	96	49%
	William Harvey Hospital	211	81	38%
	The Queen Elizabeth The Queen Mother Hospital	180	64	36%
	Conquest Hospital	181	66	36%
	**Total**	**1350**	**570**	**42%**

©2015 Picker Institute Europe. All Rights Reserved.

Table 1. *continued*

Network	Unit	Eligible Invited	Returned	Response Rate
**South West Midlands Maternity and Newborn Network**	Birmingham Women's Hospital	634	196	31%
	Birmingham Heartlands Hospital	95	29	31%
	Good Hope Hospital	99	34	34%
	Alexandra Hospital	74	28	38%
	Worcestershire Royal Hospital	211	59	28%
	**Total**	**1113**	**346**	**31%**
**South West Neonatal Network**	Yeovil District Hospital	120	54	45%
	St Michael's Hospital	191	80	42%
	Musgrove Park Hospital	198	94	47%
	Royal Cornwall Hospital	182	86	47%
	Royal Devon And Exeter Hospital	197	93	47%
	Derriford Hospital	198	77	39%
	The Great Western Hospital	198	103	52%
	Gloucestershire Royal Hospital	187	62	33%
	Southmead Hospital	196	93	47%
	**Total**	**1667**	**742**	**45%**
**Thames Valley and Wessex Neonatal Network**	St Mary's Hospital, Isle Of Wight	145	46	32%
	Wexham Park Hospital	196	70	36%
	Milton Keynes Hospital	195	79	41%
	Princess Anne Hospital	193	87	45%
	Queen Alexandra Hospital	193	82	42%
	Salisbury District Hospital	135	79	59%
	Horton General Hospital	65	28	43%
	John Radcliffe Hospital	187	92	49%
	Stoke Mandeville Hospital	197	79	40%
	**Total**	**1506**	**642**	**43%**
**The Staffordshire, Shropshire & Black Country Newborn and Maternity Network**	University Hospital Of North Staffordshire	195	58	30%
	Russells Hall Hospital	198	81	41%
	Princess Royal Hospital	192	83	43%
	**Total**	**585**	**222**	**38%**

©2015 Picker Institute Europe. All Rights Reserved.

Table 1. *continued*

Network	Unit	Eligible Invited	Returned	Response Rate
**Trent Perinatal Network**	Nottingham City Hospital	167	82	49%
	King's Mill Hospital	186	89	48%
	Queens Medical Centre	169	71	42%
	**Total**	**522**	**242**	**46%**
**Yorkshire & Humber North Neonatal Network**	Bradford Royal Infirmary Maternity Unit	166	52	31%
	York Hospital	164	72	44%
	Scarborough Hospital	133	41	31%
	Leeds Neonatal Service	392	176	45%
	Calderdale Royal Hospital	197	74	38%
	Pinderfields General Hospital	173	75	43%
	Dewsbury And District Hospital	133	45	34%
	**Total**	**1358**	**535**	**39%**
**Yorkshire & Humber South Neonatal Network**	Sheffield Children’s Hospital Neonatal Surgical Unit	188	66	35%
	Rotherham District General Hospital	146	54	37%
	Chesterfield Royal Hospital	136	64	47%
	The Jessop Wing, Sheffield	187	77	41%
	Diana Princess Of Wales Hospital	194	44	23%
	Scunthorpe General Hospital	195	55	28%
	Bassetlaw District General Hospital	61	24	39%
	Doncaster Royal Infirmary	182	72	40%
	**Total**	**1289**	**456**	**35%**

©2015 Picker Institute Europe. All Rights Reserved.

About the respondents

A total of 15,944 eligible parents were sent a questionnaire. 6,000 returned completed questionnaires giving an overall response rate of 37.6%. 85% of respondents were the baby's mother, 2% were the baby's father, and 12% of parents completed the questionnaire together. Seven guardians completed the questionnaire (<1% of all respondents).

Table 2: Respondent characteristics

**Respondent Characteristics**	**n**	**%**
Percentage of babies born at:		
Up to 29 weeks pregnant	490	8%
30 - 37 weeks pregnant	3107	52%
38 weeks pregnant or more	2362	40%
Age group (mother):		
16 - 18 years	12	<1%
19 - 24 years	591	10%
25 - 29 years	1366	23%
30 -34 years	2030	34%
35 years and older	1996	33%
Ethnic group (mother):		
White	4832	81%
Mixed	123	2%
Asian or Asian British	632	11%
Black or Black British	257	4%
Chinese or Other ethnic group	154	3%
Network Type		
Local neonatal unit	675	11%
Neonatal intensive care unit	2841	47%
Special care baby unit	2484	41%

©2015 Picker Institute Europe. All Rights Reserved.

Section Three

Overall Results

Overview of overall scores

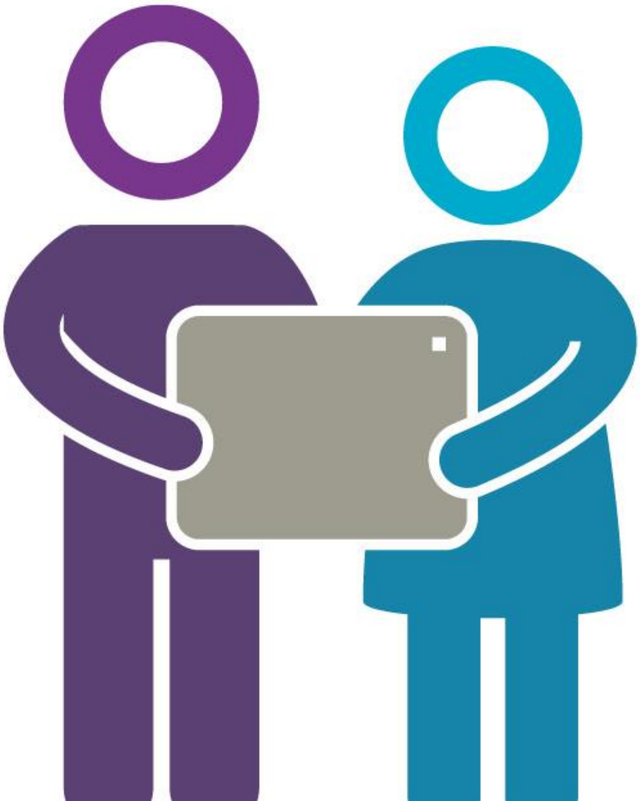

Overall results

This section provides a broad overview of the main survey findings for all participating units by questionnaire section, as well as those areas where overall, parents reported their most positive and least positive experiences of neonatal care. The results presented here are the scores for each question in the survey for which this is suitable. For an explanation of how scores are calculated and should be interpreted, refer to Section 1.

The survey results, including breakdowns by neonatal network are discussed in more detail in Section 4.

Table 3. Overall score by questionnaire section

A. Before your baby was born

Higher scores are better

Question	Score
**A2** Before your baby was born (i.e. during pregnancy or labour), did a member of staff from the neonatal unit talk to you about what to expect after the birth?	54

B. Your baby's admission to neonatal care

Question	Score
**B2** After you gave birth, were you offered a photograph of your baby?	63
**B3** After you gave birth, were you ever cared for in the same ward as mothers who had their baby with them?	55
**B4** After your baby was admitted to the neonatal unit, were you able to see your baby as soon as you wanted?	91

©2015 Picker Institute Europe. All Rights Reserved.

C. Staff on the neonatal unit

Higher scores are better

Question	Score
**C1** When you visited the unit, did the staff caring for your baby introduce themselves to you?	79
**C2** Were you given enough information about the neonatal unit (such as rules, procedures and facilities for parents)	76
**C3** Was the purpose of the machines, monitors and alarms used in the neonatal unit clearly explained to you?	73
**C4** Were infection control practices explained to you, such as hand washing and procedures for visitors?	85
**C5** Were you told which nurse was responsible for your baby's care each day s/he was in the neonatal unit?	90
**C6** Were you able to talk to staff on the unit about your worries and concerns?	88
**C7** Were you able to speak to a doctor about your baby as much as you wanted?	66
**C8** Were the nurses on the unit sensitive to your emotions and feelings?	85
**C9** Were the doctors on the unit sensitive to your emotions and feelings?	83
**C10** In your opinion, was important information about your baby passed on from one member of staff to another?	84
**C11** Did staff give you conflicting information about your baby's condition or care?	67
**C12** Did staff refer to your baby by his/her first name?	90
**C13** Overall, did you have confidence and trust in the staff caring for your baby?	92

©2015 Picker Institute Europe. All Rights Reserved.

D. Your involvement in your baby's care

Higher scores are better

Question	Score
**D1** Were you involved as much as you wanted in the day-to-day care of your baby, such as nappy changing and feeding?	89
**D2** Did you have as much skin- to-skin contact with your baby as you wanted?	72
**D3** Did the neonatal staff include you in discussions about your baby's care and treatment?	78
**D4** Were you told about any changes in your baby's condition or care?	89
**D5** When a ward round was taking place, were you allowed to be present when your baby was being discussed?	80
**D6** Where possible, did staff arrange your baby's care (such as weighing, bathing) to fit in with your usual visiting times?	74
**D7** Overall, did staff help you feel confident in caring for your baby?	89
**D8** If you wanted to express breast milk for your baby, were you given the support you needed from neonatal staff?	86
**D9** When you were in the neonatal unit, were you given the feeding equipment you needed for expressing, such as a breast pump and sterilisation equipment?	87
**D10** Were you given enough privacy in the neonatal unit for expressing milk and/or breastfeeding your baby?	88
**D11** If you wanted to breastfeed your baby, were you given enough support to do this from neonatal staff?	84
**D12** If you fed your baby formula milk, were you given enough support to do this from neonatal staff?	85

E. Environment and facilities

Question	Score
**E1** Were you given enough privacy when discussing your baby's care on the neonatal unit with staff?	84
**E2** Was there enough space for you to sit alongside your baby's cot in the unit?	82
**E3** In your opinion, was there adequate security on the neonatal unit?	88
**E4** If you wanted to stay overnight to be close to your baby, did the hospital offer you accommodation?	70
**E5** Were you able to visit your baby on the unit as much as you wanted to? (please only think about unit-related reasons and not personal reasons such as needing to care for other children)	95

©2015 Picker Institute Europe. All Rights Reserved.

F. Information and support for parents

Higher scores are better

Question	Score
**F1** If you asked questions about your baby's condition and treatment, did you get answers you could understand?	88
**F2** Were you given enough written information to help you understand your baby’s condition and treatment?	53
**F4** Were you offered emotional support or counselling services from neonatal unit staff?	57
**F5** Were you given enough information about help you could get with expenses related to your baby's stay in the neonatal unit (such as travelling/parking expenses, hardship fund or food vouchers?	41
**F6** Did staff give you any information about parent support groups, such as Bliss or other local groups?	53

G. Leaving the neonatal unit

Question	Score
**G3** Were you offered overnight accommodation with your baby at the hospital before they left the neonatal unit?	95
**G4** Did you feel prepared for your baby’s discharge from neonatal care?	86
**G5** Were you given enough information on what to expect in terms of your baby’s progress and recovery?	76
**G6** How likely are you to recommend this neonatal unit to friends and family, if their baby needed similar care or treatment?	91

©2015 Picker Institute Europe. All Rights Reserved.

Ranked Scores

This section presents the areas where overall, parents reported their most positive and least positive experience of neonatal care. Scores are ranked from highest to lowest. Those questions with the highest scores shows where overall participating neonatal units are doing well. Those with lower scores present where overall, units have the most room for improvement. For an explanation of how scores are calculated and should be interpreted, refer to Section 1. Higher scores are better.

Table 4. Ranked overall scores

Score 90 +

Question	Score
**G3** Were you offered overnight accommodation with your baby at the hospital before they left the neonatal unit?	95
**E5** Were you able to visit your baby on the unit as much as you wanted to?	95
**C13** Overall, did you have confidence and trust in the staff caring for your baby?	92
**G6** How likely are you to recommend this neonatal unit to friends and family, if their baby needed similar care or treatment?	91
**B4** After your baby was admitted to the neonatal unit, were you able to see your baby as soon as you wanted?	91
**C5** Were you told which nurse was responsible for your baby's care each day s/he was in the neonatal unit?	90
**C12** Did staff refer to your baby by his/her first name?	90

©2015 Picker Institute Europe. All Rights Reserved.

Score 85 - 89

Question	Score
**D4** Were you told about any changes in your baby's condition or care?	89
**D7** Overall, did staff help you feel confident in caring for your baby?	89
**D1** Were you involved as much as you wanted in the day-to-day care of your baby, such as nappy changing and feeding?	89
**E3** In your opinion, was there adequate security on the neonatal unit?	88
**F1** If you asked questions about your baby's condition and treatment, did you get answers you could understand?	88
**C6** Were you able to talk to staff on the unit about your worries and concerns?	88
**D10** Were you given enough privacy in the neonatal unit for expressing milk and/or breastfeeding your baby?	88
**D9** When you were in the neonatal unit, were you given the feeding equipment you needed for expressing, such as a breast pump and sterilisation equipment?	87
**D8** If you wanted to express breast milk for your baby, were you given the support you needed from neonatal staff?	86
**G4** Did you feel prepared for your baby's discharge from neonatal care?	86
**C8** Were the nurses on the unit sensitive to your emotions and feelings?	85
**D12** If you fed your baby formula milk, were you given enough support to do this from neonatal staff?	85
**C4** Were infection control practices explained to you, such as hand washing and procedures for visitors?	85

Score 79 - 84

Question	Score
**D11** If you wanted to breastfeed your baby, were you given enough support to do this from neonatal staff?	84
**C10** In your opinion, was important information about your baby passed on from one member of staff to another?	84
**E1** Were you given enough privacy when discussing your baby's care on the neonatal unit with staff?	84
**C9** Were the doctors on the unit sensitive to your emotions and feelings?	83
**E2** Was there enough space for you to sit alongside your baby's cot in the unit?	82
**D5** When a ward round was taking place, were you allowed to be present when your baby was being discussed?	80
**C1** When you visited the unit, did the staff caring for your baby introduce themselves to you?	79

©2015 Picker Institute Europe. All Rights Reserved.

Score 70 - 79

Question	Score
**D3** Did the neonatal staff include you in discussions about your baby's care and treatment?	78
**C2** Were you given enough information about the neonatal unit (such as rules, procedures and facilities for parents)	76
**G5** Were you given enough information on what to expect in terms of your baby's progress and recovery?	76
**D6** Where possible, did staff arrange your baby's care (such as weighing, bathing) to fit in with your usual visiting times?	74
**C3** Was the purpose of the machines, monitors and alarms used in the neonatal unit clearly explained to you?	73
**D2** Did you have as much skin- to-skin contact with your baby as you wanted?	72
**E4** If you wanted to stay overnight to be close to your baby, did the hospital offer you accommodation?	70

Score below 70

Question	Score
**C11** Did staff give you conflicting information about your baby's condition or care?	67
**C7** Were you able to speak to a doctor about your baby as much as you wanted?	66
**B2** After you gave birth, were you offered a photograph of your baby?	63
**F4** Were you offered emotional support or counselling services from neonatal unit staff?	57
**B3** After you gave birth, were you ever cared for in the same ward as mothers who had their baby with them?	55
**A2** Before your baby was born (i.e. during pregnancy or labour), did a member of staff from the neonatal unit talk to you about what to expect after the birth?	54
**F2** Were you given enough written information to help you understand your baby's condition and treatment?	53
**F6** Did staff give you any information about parent support groups, such as Bliss or other local groups?	53
**F5** Were you given enough information about help you could get with expenses related to your baby's stay in the neonatal unit (such as travelling/parking expenses, hardship fund or food vouchers?	41

©2015 Picker Institute Europe. All Rights Reserved.

Section Four

Overall Results by Network

Overview by section

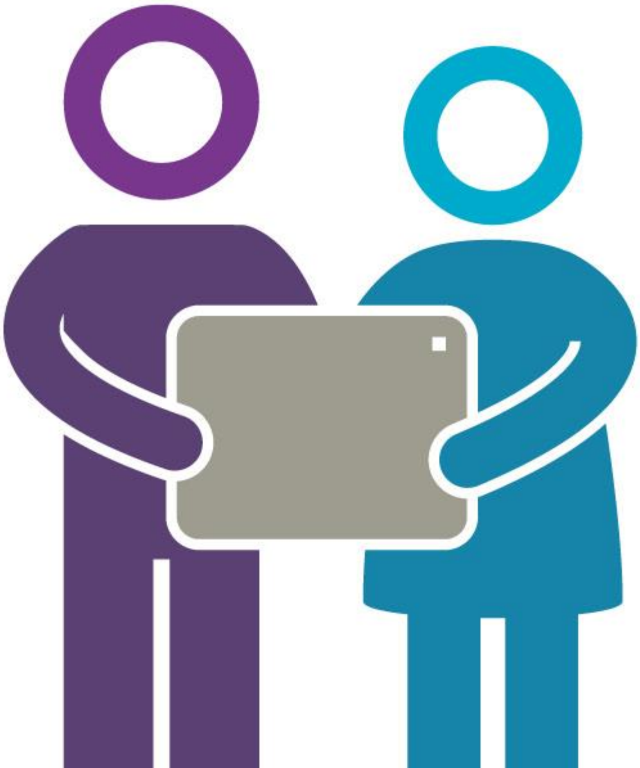

Overall Results by Network

This section shows the survey results for all participating neonatal units by network, and a comparison against the average results for all participating networks. Findings for Lancashire and South Cumbria Neonatal Network are not presented to maintain anonymity as there was only one participating unit from this network. Their results are however included in the overall average. The results are presented according to the sections in the questionnaire. Please refer to the frequency tables in Appendix A for the number of respondents to each question. Cross tabulations for each question by network is available upon request from the Picker Institute.

A. Before your baby was born

Two thirds (66%; n=3880) of parents stated they did not know before their baby was born (i.e. during pregnancy or labour) that their baby might need care in a neonatal unit, compared to 34% (n=2031) who did.

Of those who did know, 44% (n=845) stated that staff from the neonatal unit definitely spoke to them about what to expect after the birth. 22% (n=405) noted this happened to some extent and 34% (n=669) said staff did not speak to them about what to expect. Parents from the Staffordshire, Shropshire and Black Country network (47%; n=31) were most likely to note that staff did not speak to them about what to expect compared to other networks. Those respondents from Yorkshire and Humber South network (58%, n=86) and Greater Manchester network (57%, n=80) were most likely to state that staff definitely spoke to them about what to expect after the birth compared to other networks. Refer to figure 1, graph A2 for full details.

Figure 1: Section A. Graphs

Graph A2. Before your baby was born (i.e. during pregnancy or labour), did a member of staff from the neonatal unit talk to you about what to expect after the birth? (Overall n=1919)

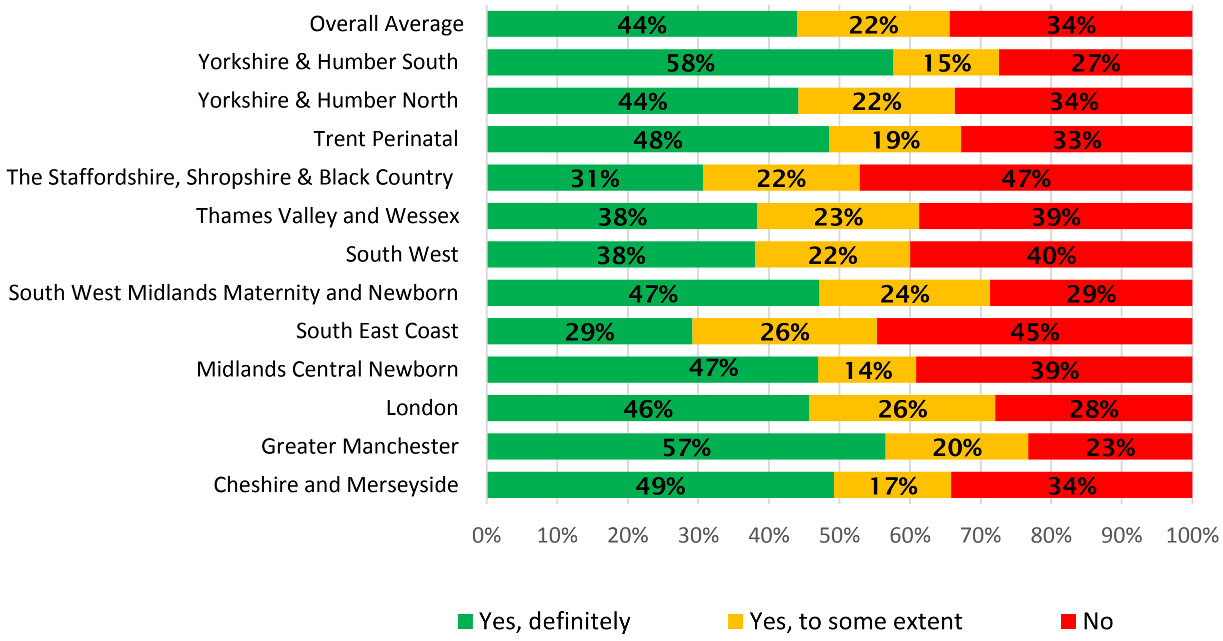

©2015 Picker Institute Europe. All Rights Reserved.

B. Your baby's admission to neonatal care

Parents were asked about their experience of their baby's admission to the neonatal unit. The parents responding from Yorkshire and Humber North network who were not discharged after they gave birth were least likely to share a ward with mothers who had their baby with them which bothered them (19%; n=83), compared to other networks (figure 2, graph B3). Younger mothers who were not discharged after they gave birth were more likely than older mothers to report that it bothered them when they were cared for in the same ward as mothers who had their baby with them (see table 5).

Table 5. Percentage of respondents to question B3, by age group

B3. After you gave birth, were you ever cared for in the same ward as mothers who had their baby with them? By mother's age group (n=5198)

	16-25 years	26-29 years	30-34 years	35 + years
**Yes, and this bothered me**	36.7%	32.6%	30.8%	27.3%
**Yes, but I did not mind**	26.9%	25.7%	24.6%	31.5%
**No, I stayed in a separate room/area**	36.3%	41.7%	44.4%	41.2%

Compared with other units, a greater proportion of parents at Yorkshire and Humber North (65%; n=293) were offered photograph of their baby. Respondents from Trent Perinatal (32%; n=62) and London (32%; n=246) networks were least likely to be offered a photograph of their baby compared to other networks (figure 2, graph B2). 40% (n=197) of parents from South East Coast network stated that it bothered them that they shared a room with mothers who had their baby with them.

The results were similar across networks for parents being able to see their baby as soon as they wanted (see figure 2, graph B4).

©2015 Picker Institute Europe. All Rights Reserved.

Figure 2: Section B. Graphs

Graph B2. After you gave birth, were you offered a photograph of your baby? (Overall n=4972)

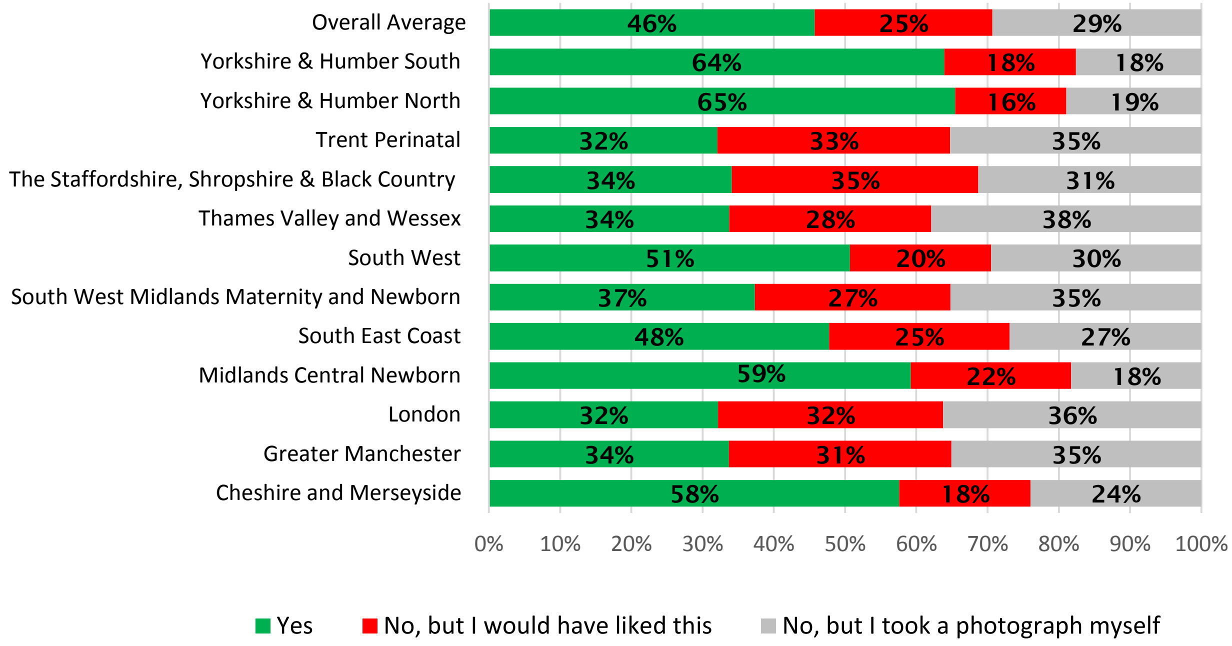

Graph: B3. After you gave birth, were you ever cared for in the same ward as mothers who had their baby with them? (Overall n=5167)

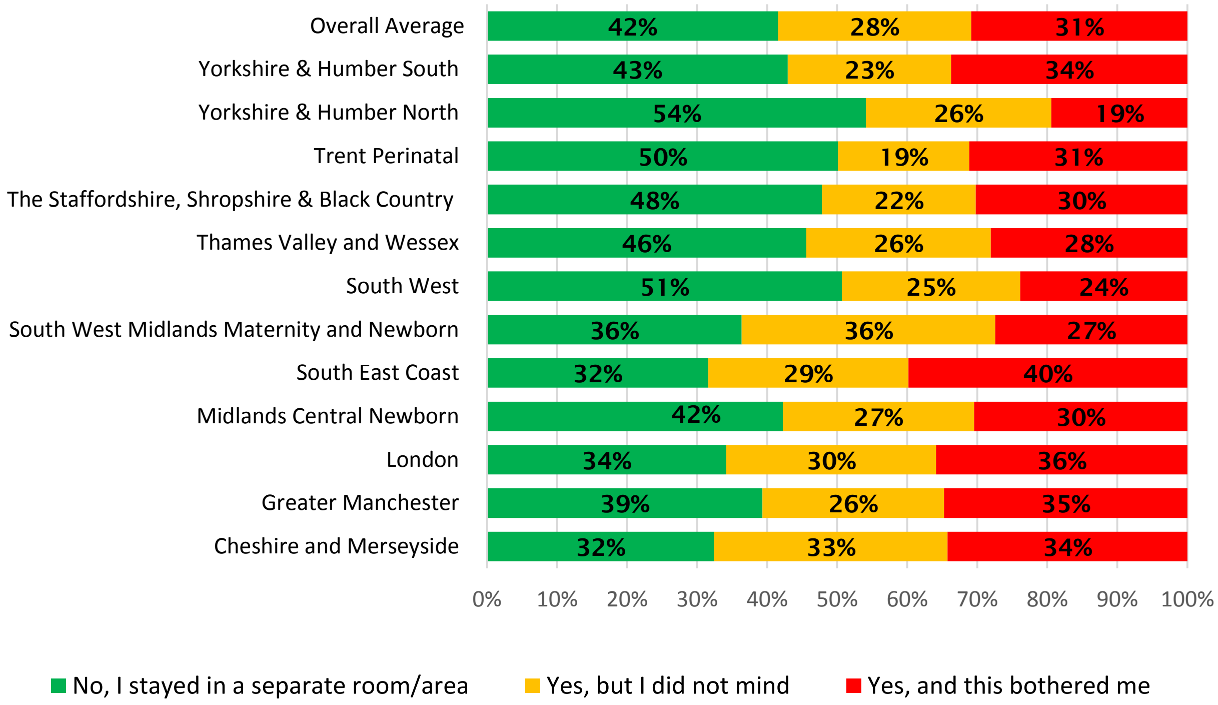

©2015 Picker Institute Europe. All Rights Reserved.

Graph B4. After your baby was admitted to the neonatal unit, were you able to see your baby as soon as you wanted? (Overall n=3858)

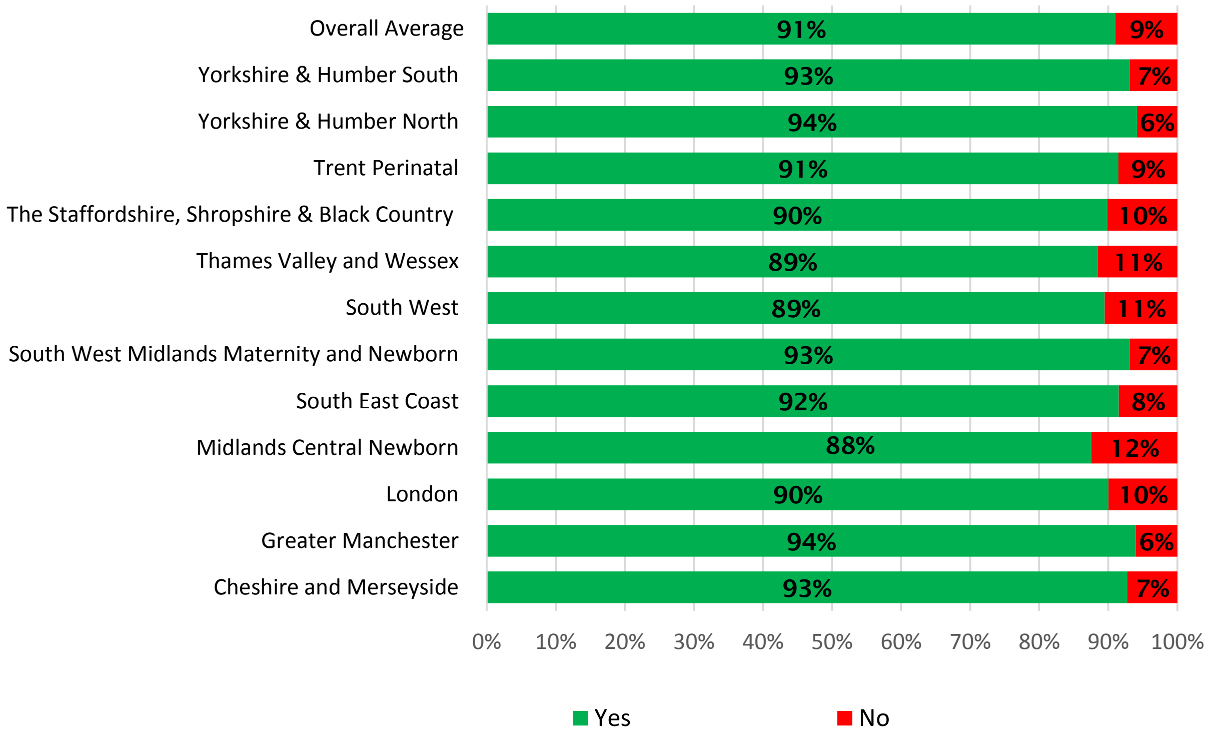

©2015 Picker Institute Europe. All Rights Reserved.

C. Staff on the neonatal unit

Figure 3 below maps the proportion of parents who responded positively to each question in section C “Staff on the neonatal unit” for all networks, and the overall average. It highlights the variations between networks for each question, and indicates that overall the London network has the most room for improvement. Whereas Trent Perinatal and Greater Manchester networks have the highest proportion of parents responding positively on most questions. The diagram also highlights the areas that most networks could improve such as: C7, parents being able to speak to doctors as much as they want to; C3, receiving information about the machines in the unit; and C11, parents receiving conflicting information from staff members. Higher percentages are better.

Overall, parents had confidence and trust in the staff caring for their baby, with 87% (n=5140) stating they always (or nearly always) had confidence and trust, 11% (n=667) sometimes did, and only 2% (n=112) stating they did not (figure 4, graph C13).

A high proportion of parents knew each day which nurse was responsible for their baby’s care. And for most networks parents stated that, where possible, staff referred to their baby by their first name. Trent Perinatal network had the highest proportion of parents (96%, n=230) stating they were always told which nurse was responsible for their baby’s care each day compared to other networks (figure 4, graph C5).

That said, overall a third of parents (33%; n=1911) noted that only some of the staff introduced themselves. Further, a large proportion (55%; n=3144) stated they were not fully able to talk to a doctor as much as they wanted. This trend was similar across all networks, however Greater Manchester network and Trent Perinatal network had the highest proportion of parents stating all staff introduced themselves and they could speak to doctors as much as they wanted (see figure 4, graphs C1 and C7 below).

Overall, parents were somewhat positive about doctors and nurses being sensitive to their emotions and feelings, and being able to talk to staff if they had worries or concerns (see figure 4, graphs C6, C8 and C9). However, approximately one third of respondents from the London network (32%; n=298) and South West Midlands network (31%; n= 99) stated that nurses were not fully sensitive to their emotions and feelings. Parents from the London, South East Coast, and South West Midlands networks were the mostly likely compared to other networks to report that doctors were not fully sensitive to their emotions and feelings (see figure 4, graph C9).

Sharing information about the neonatal unit as well as the purpose of machines, monitors and alarms could be improved across the networks with just less than half of parents stating this did not fully happen (see figure 4, graph C3). Parents mostly felt (73%, n=4078) that important information was passed on from one member of staff to another. However, only just over half of parents (56%; n=3300) stated they did not receive conflicting information about their baby’s condition or care, compared to 20% (n=1177) who stated they often received conflicting information, and 24% (n=1391) who sometimes did. Greater Manchester network had the highest proportion of parents stating that they did receive conflicting information compared to other networks (figure 4, graph C11).

©2015 Picker Institute Europe. All Rights Reserved.

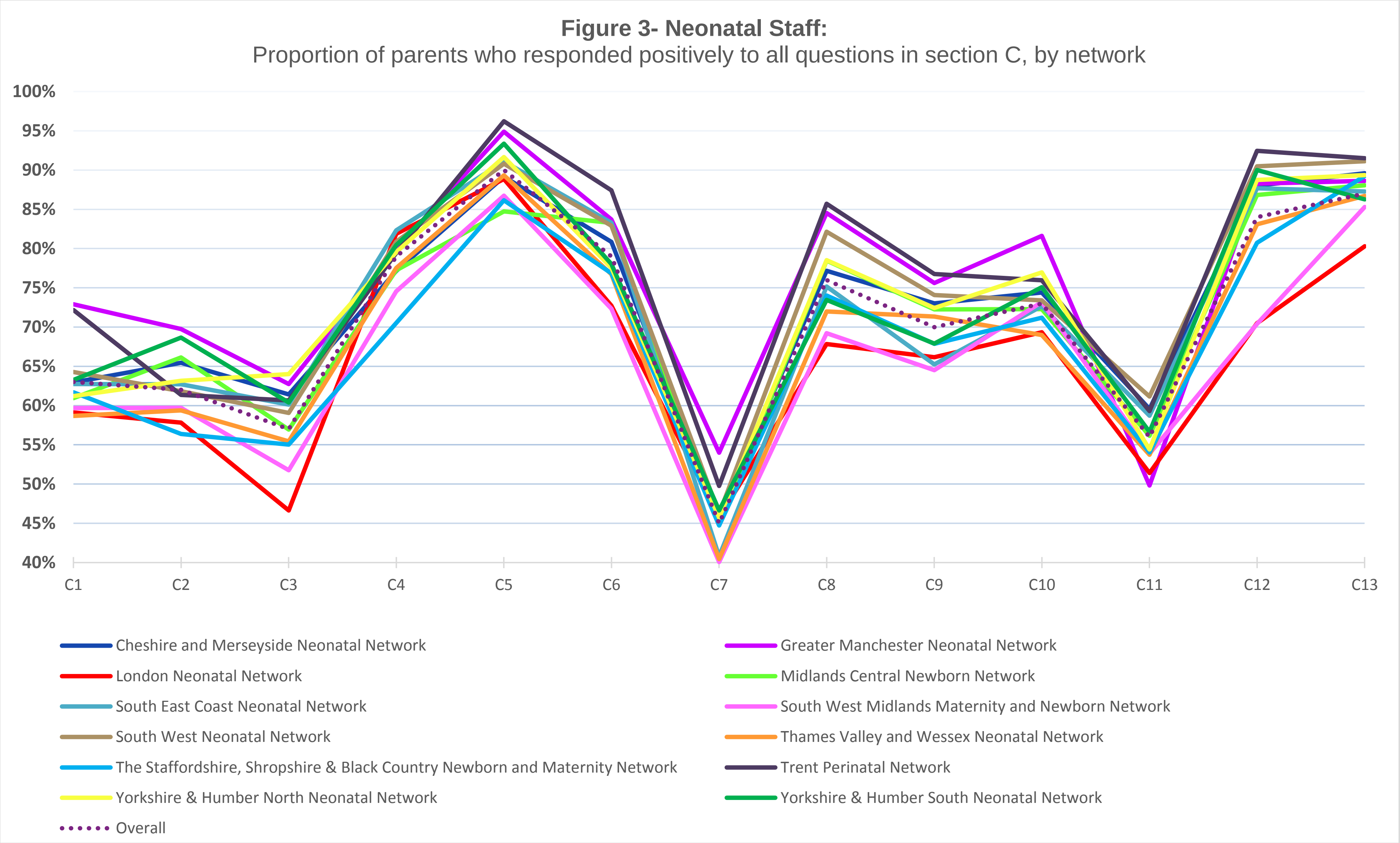
Figure 3- Neonatal Staff:
Proportion of parents who responded positively to all questions in section C, by network
100%
95%
90%
85%
80%
75%
70%
65%
60%
55%
50%
45%
40%
C1
C2
C3
C4
C5
C6
C7
C8
C9
C10
C11
C12
C13
Cheshire and Merseyside Neonatal Network
Greater Manchester Neonatal Network
London Neonatal Network
Midlands Central Newborn Network
South East Coast Neonatal Network
South West Midlands Maternity and Newborn Network
South West Neonatal Network
Thames Valley and Wessex Neonatal Network
The Staffordshire, Shropshire & Black Country Newborn and Maternity Network
Trent Perinatal Network
Yorkshire & Humber North Neonatal Network
Yorkshire & Humber South Neonatal Network
Overall

Figure 4: Section C. Graphs

Graph C1. When you visited the unit, did the staff caring for your baby introduce themselves to you? (Overall n=5804)

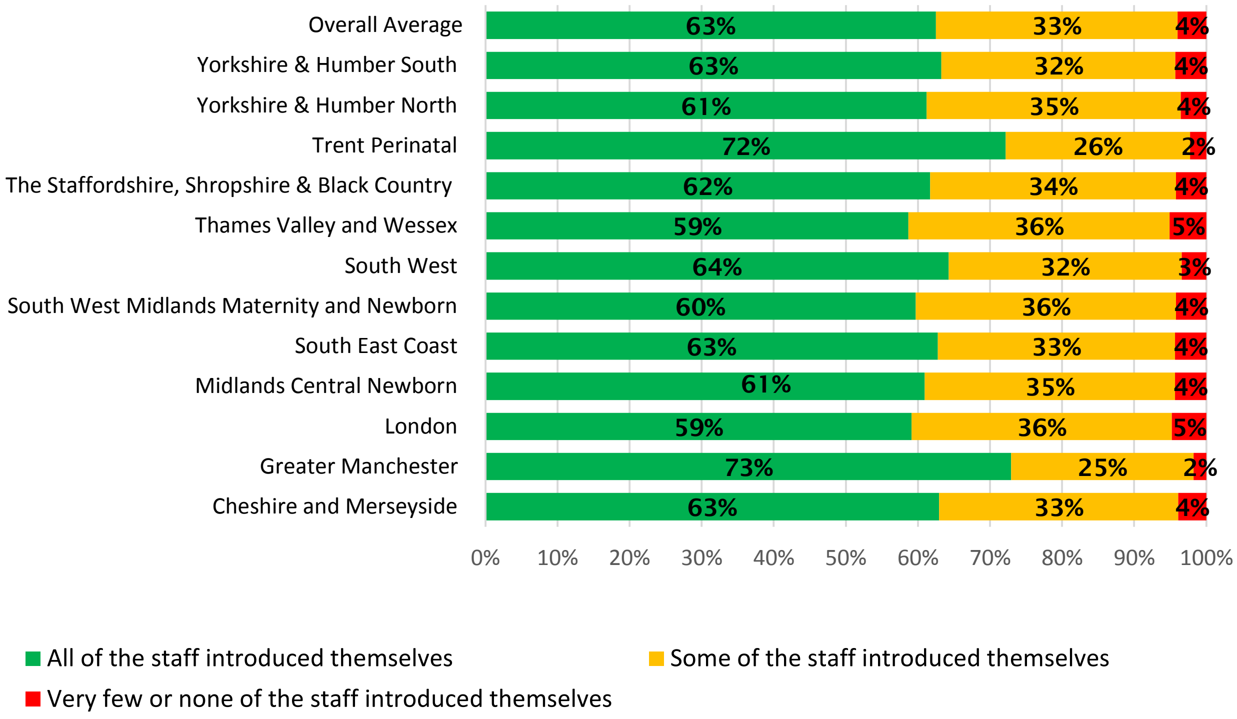

Graph C2. Were you given enough information about the neonatal unit (such as rules, procedures and facilities for parents?) (Overall n=5804)

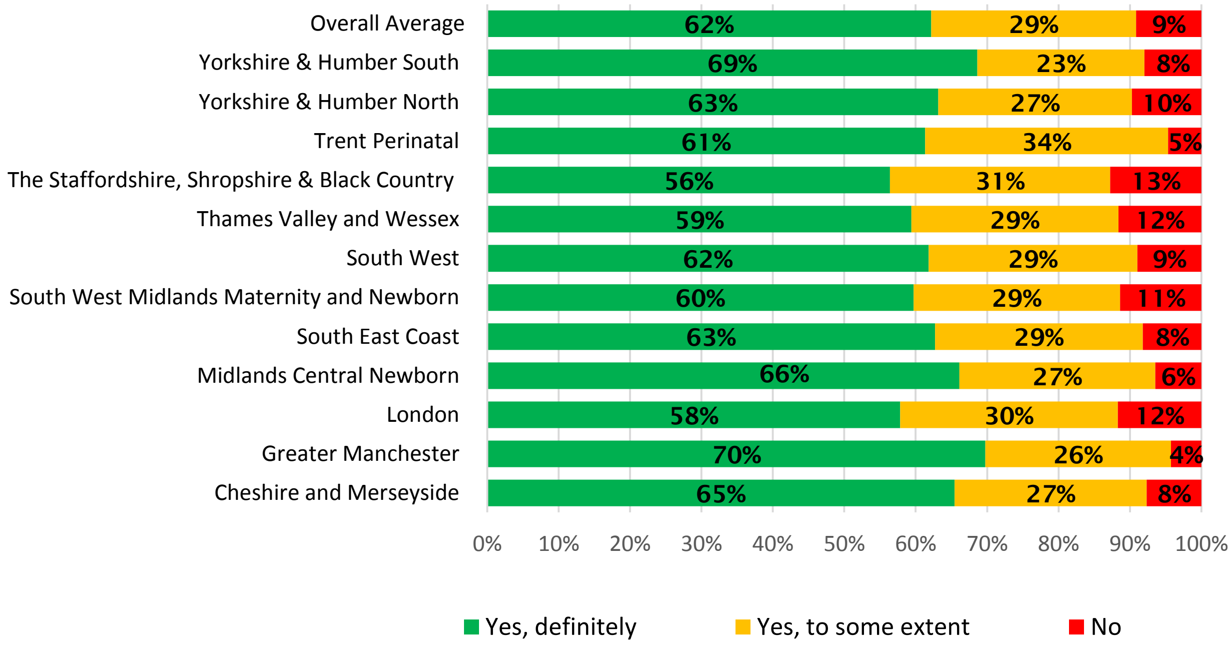

©2015 Picker Institute Europe. All Rights Reserved.

Graph C3. Was the purpose of the machines, monitors and alarms used in the neonatal unit clearly explained to you? (Overall n=5767)

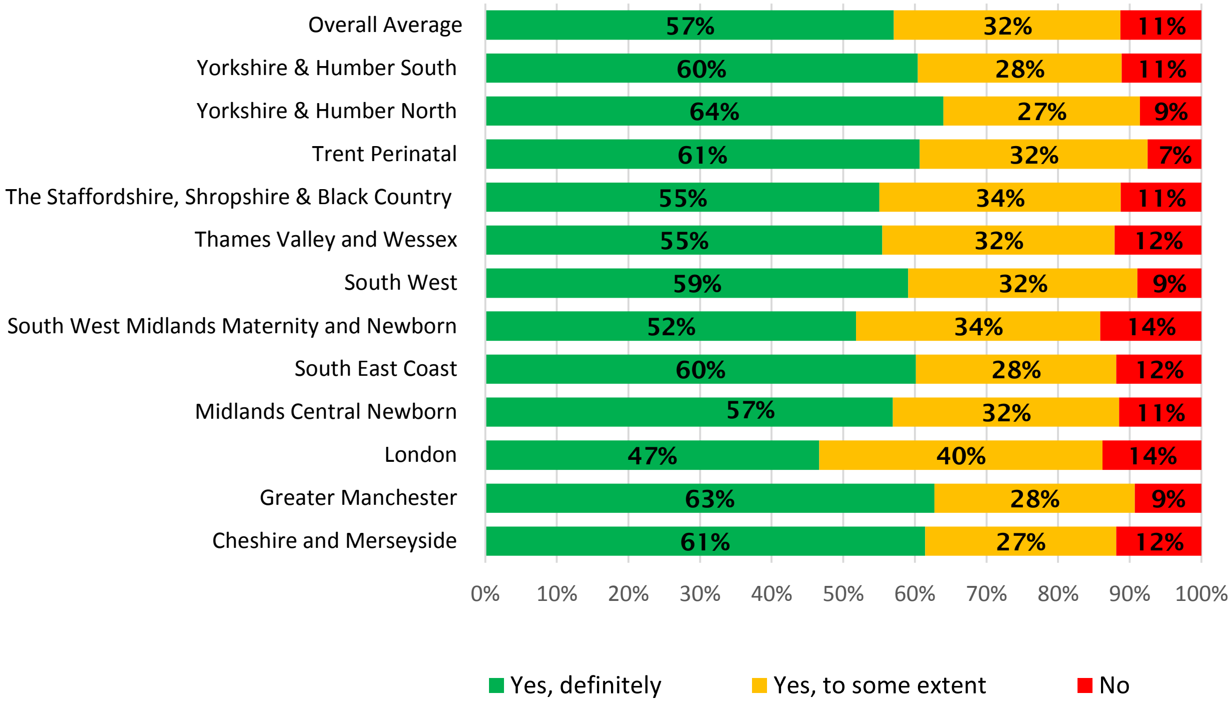

Graph C4. Were infection control practices explained to you, such as hand washing and procedures for visitors? (Overall n=5820)

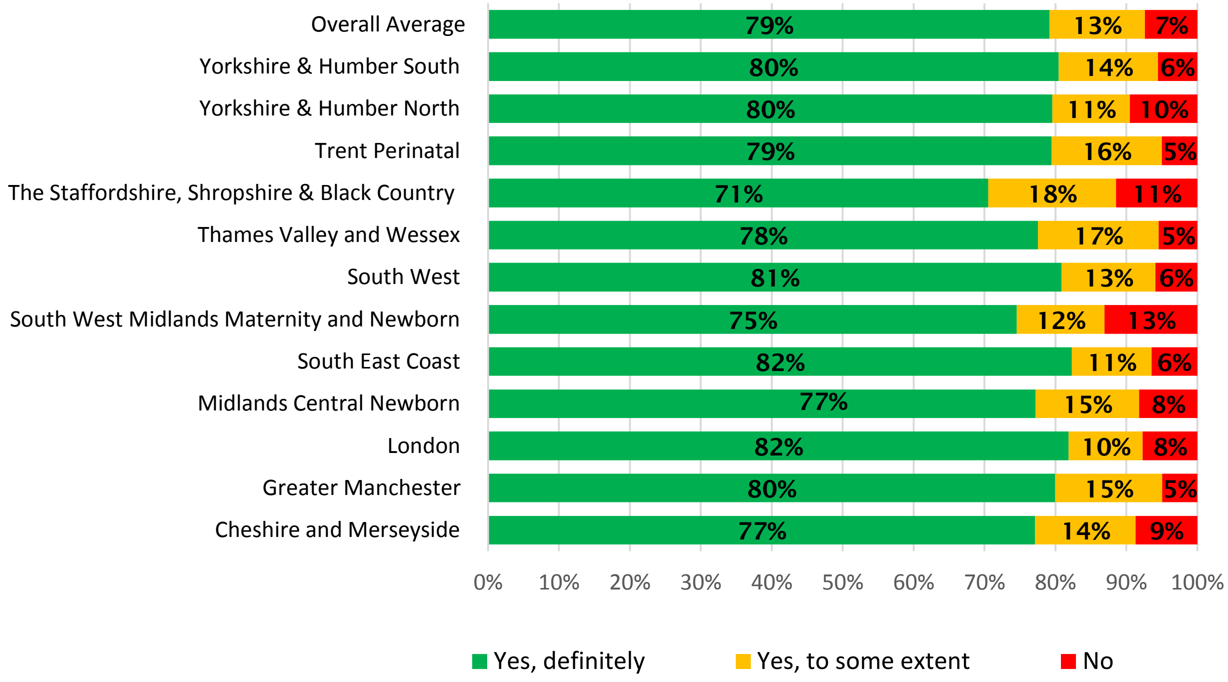

©2015 Picker Institute Europe. All Rights Reserved.

Graph C5. Were you told which nurse was responsible for your baby's care each day s/he was in the neonatal unit? (Overall n= 5888)

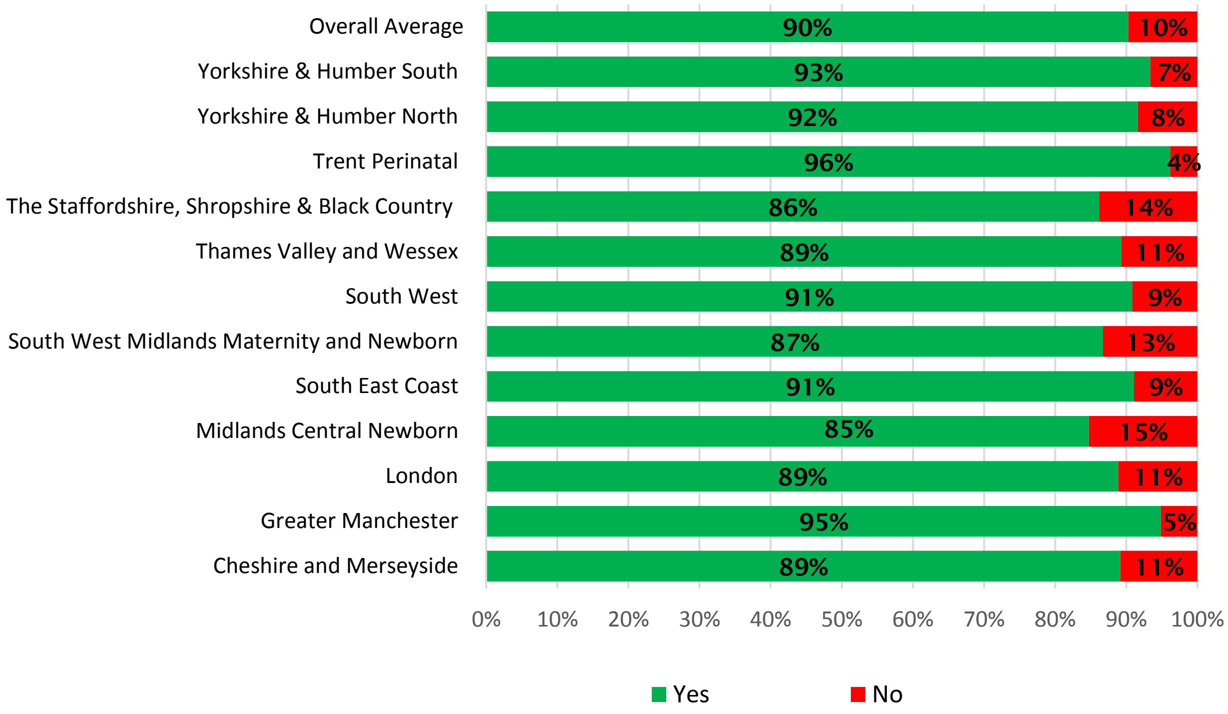

Graph C6. Were you able to talk to staff on the unit about your worries and concerns? (Overall n=5848)

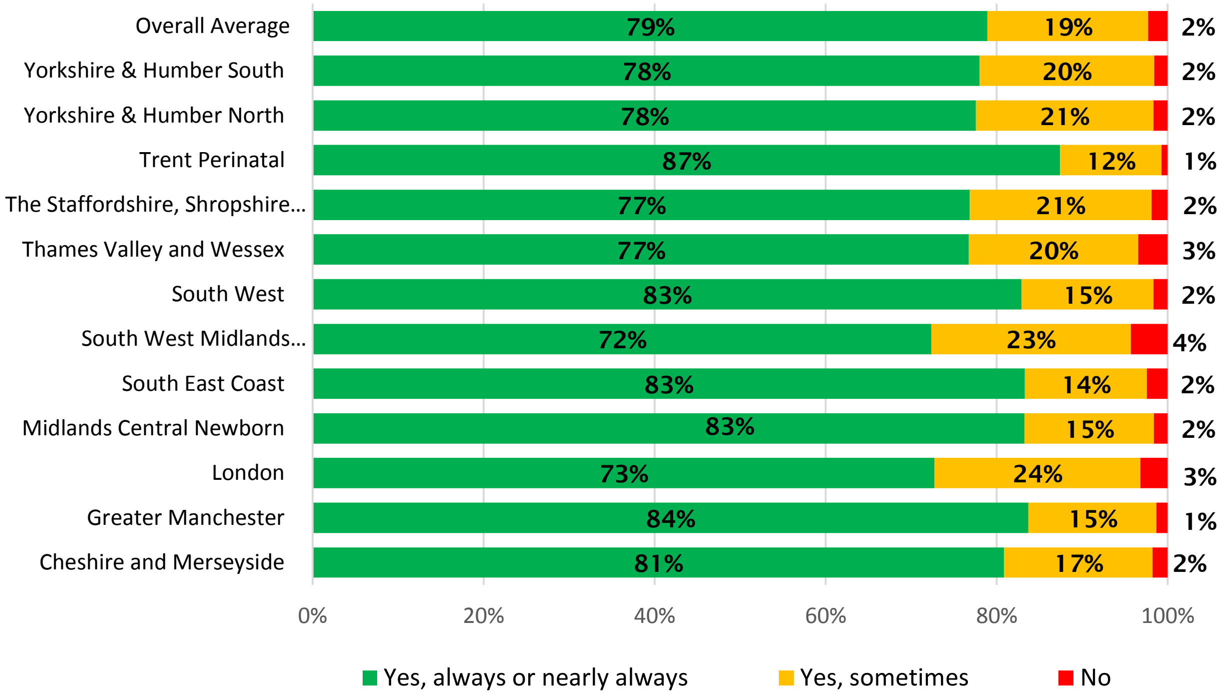

©2015 Picker Institute Europe. All Rights Reserved.

Graph C7. Were you able to speak to a doctor about your baby as much as you wanted? (Overall n=5687)

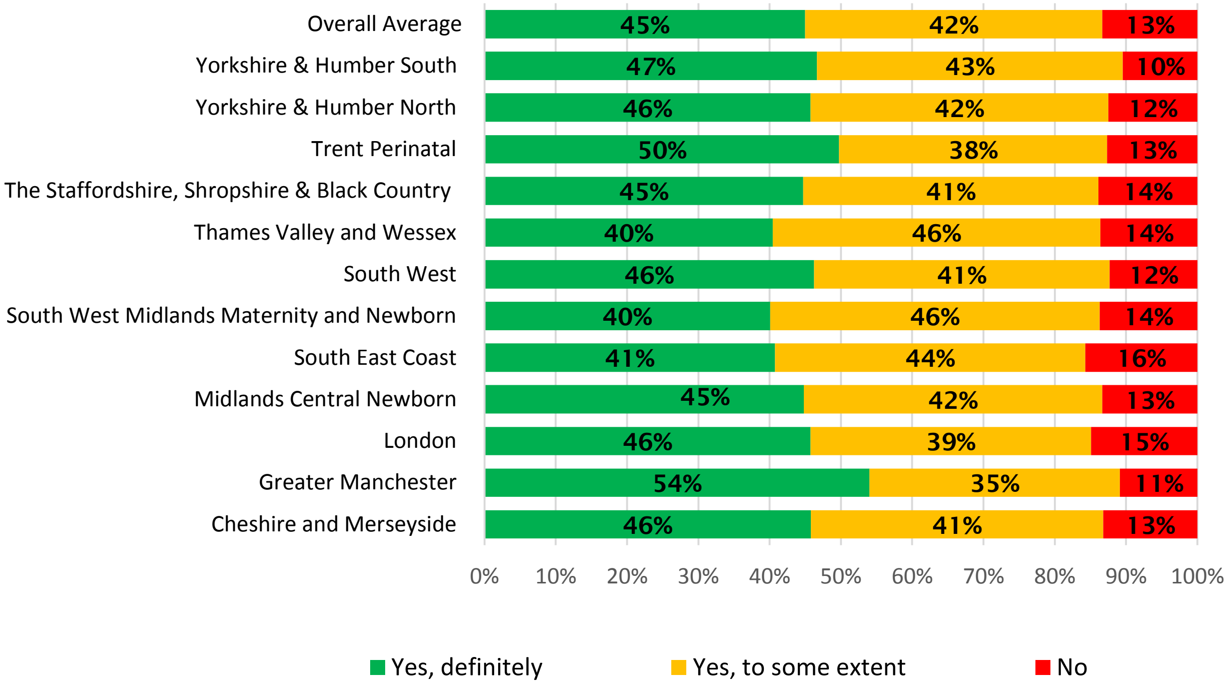

Graph C8. Were the nurses on the unit sensitive to your emotions and feelings? (Overall n=5786)

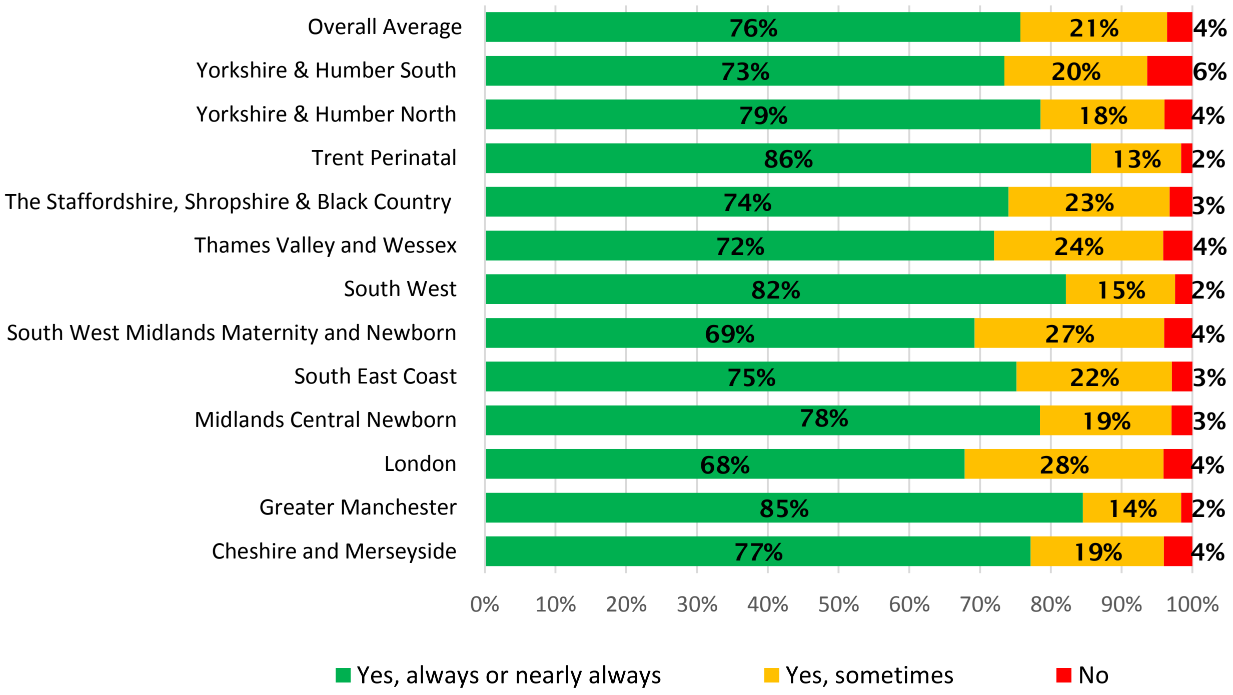

©2015 Picker Institute Europe. All Rights Reserved.

Graph C9. Were the doctors on the unit sensitive to your emotions and feelings? (Overall n=5365)

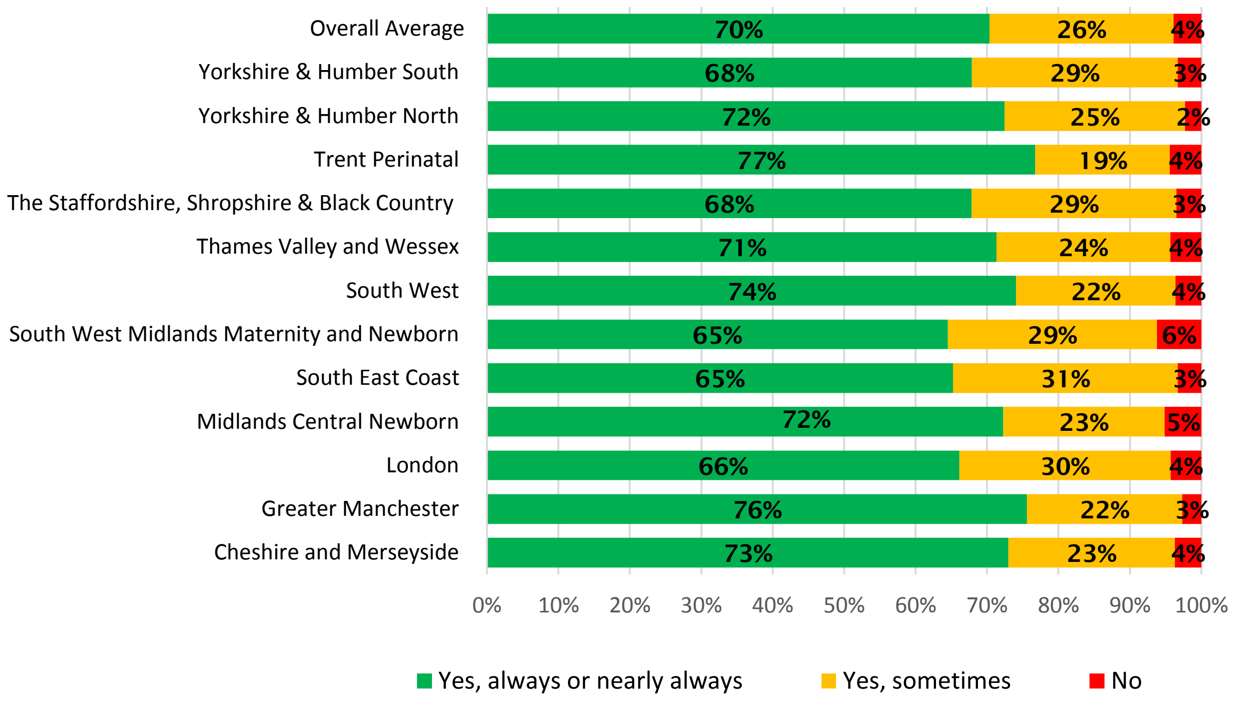

Graph C10. In your opinion, was important information about your baby passed on from one member of staff to another? (Overall n= 5603)

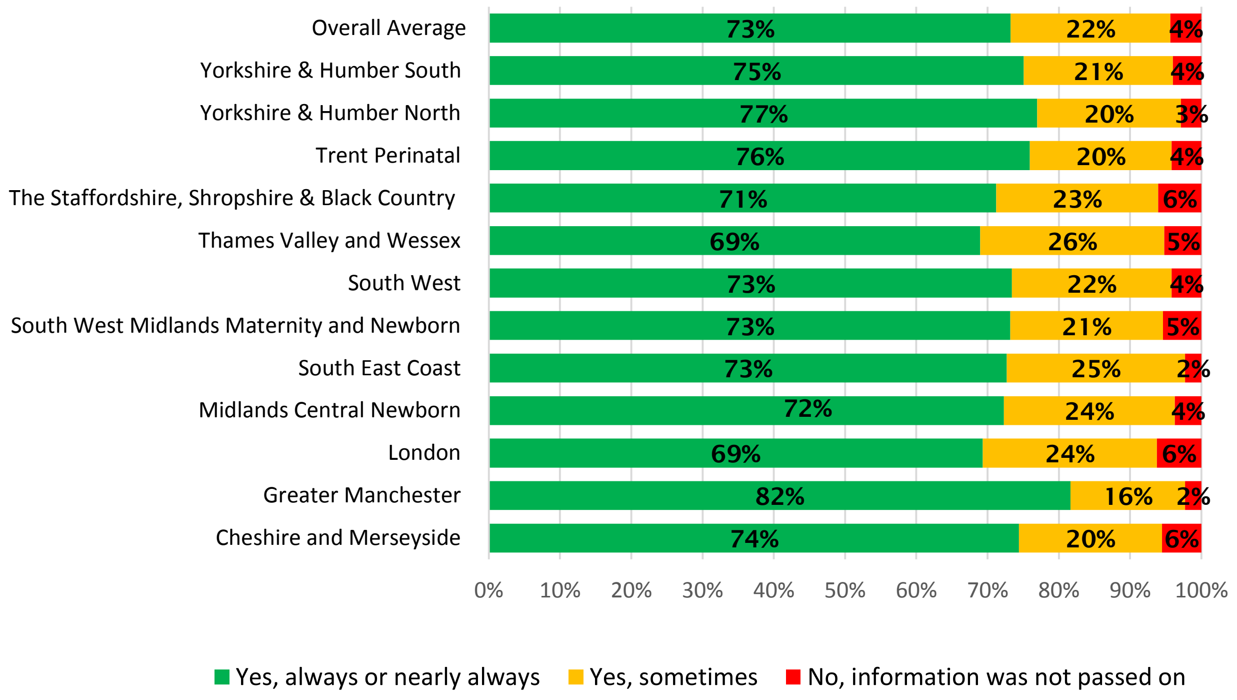

©2015 Picker Institute Europe. All Rights Reserved.

Graph C11. Did staff give you conflicting information about your baby's condition or care? (Overall n=5868)

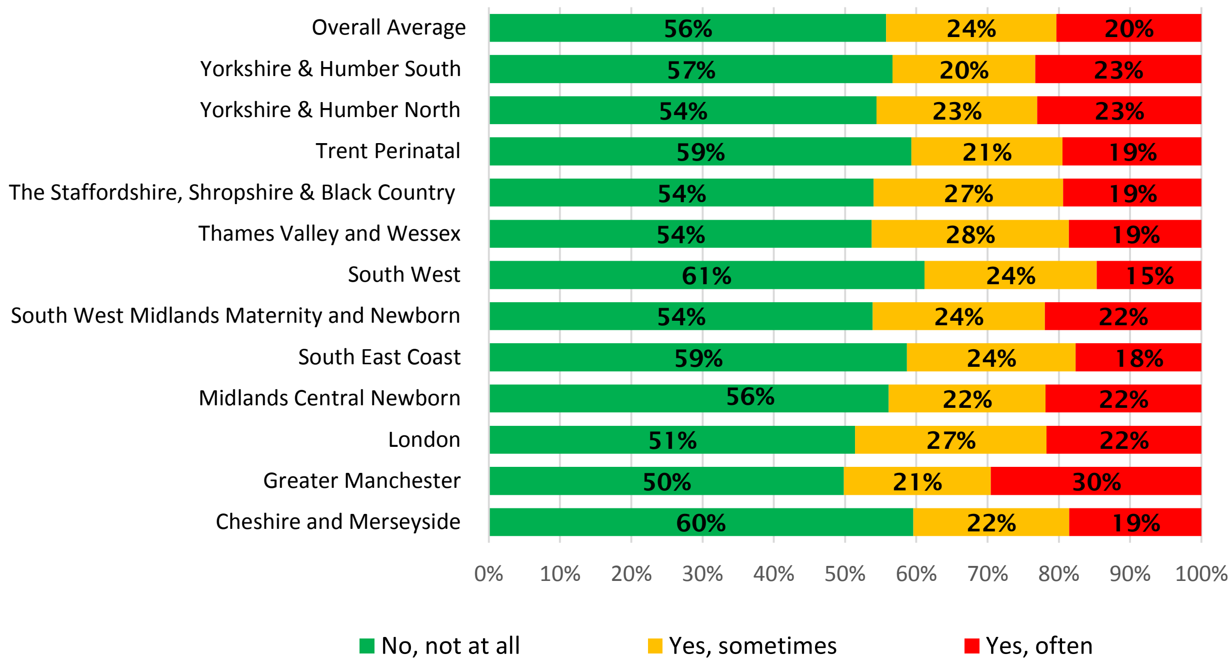

Graph C12. Did staff refer to your baby by his/her first name? (Overall n=5459)

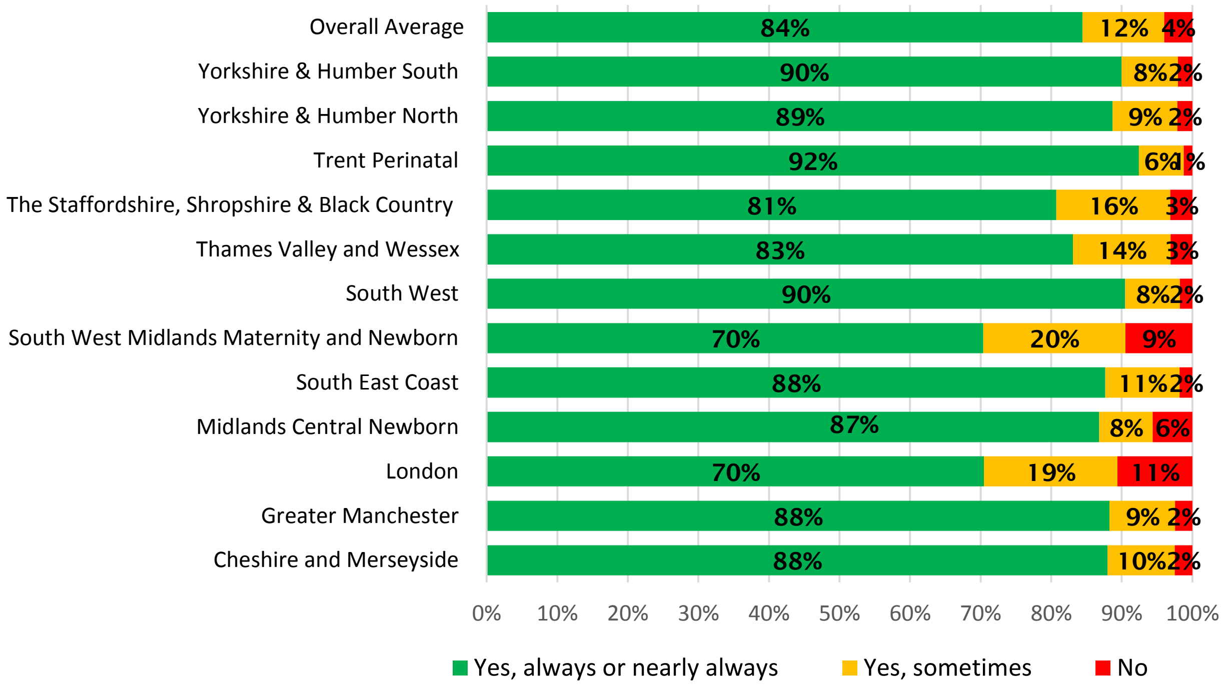

©2015 Picker Institute Europe. All Rights Reserved.

Graph C13. Overall, did you have confidence and trust in the staff caring for your baby? (Overall n=5919)

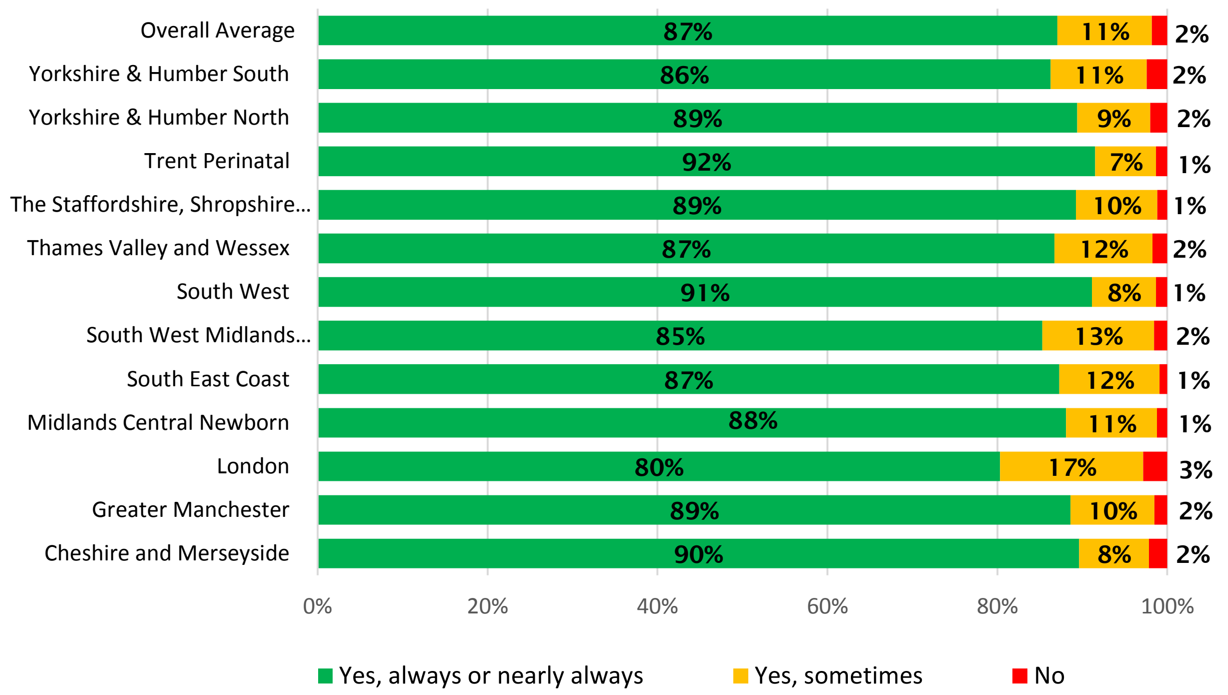

©2015 Picker Institute Europe. All Rights Reserved.

D. Your involvement in your baby's care

Parents were asked numerous questions about involvement in their baby's care. Figure 5 below maps the proportion of parents who responded positively to questions about being "involved in care" (questions D1 – D7) by network. It highlights the variations between networks for each question in this section. The figure indicates that London network has room for improvement on most questions, particularly with parents being allowed to be present during ward rounds when their baby is being discussed (D5); and arranging the baby's care to fit in with parents usual visiting times (D6).

Figure 6 below presents the proportion of parents who responded positively to questions about "Feeding" (questions D8-D12), by network. Overall, the London, and South West Midlands Maternity and Newborn networks have the most room for improvement compared to other networks. There was the most variation among networks for questions D10-12: whether mothers were given privacy to express milk or breastfeed; receiving support to breastfeed from staff; and receiving support if they used baby formula.

Overall, parents were generally as involved as much they wanted to be in day-to-day care such as nappy changing and feeding, and were told about changes in their baby's condition (see figure 7, graphs D1 and D4). However, over a third of parents (38%, n=2208) stated they were not included in discussions about their baby's care and treatment. Parents from the London network (44%, n=426) and Thames Valley network (43%, n=273) were most likely compared to other trusts to state they were not always included in discussions (see figure 7, graph D3). The London network also had the largest proportion of parents (42%, n=337) compared to other networks who reported they were not always allowed to be present during a ward round when their baby was being discussed (see figure 7, graph D5).

Just over a third of parents did not always feel staff arranged their baby's care to fit in with their usual visiting times (64%, n=2198), and were not able to have as much skin-to-skin contact as they wanted (60%, n=3070). (See figure 7, graphs D6 and D2).

That said, a large proportion of parents (81%, n=4752) stated that staff did help them feel confident in caring for their child (figure 7, graph D7). This is evident in the proportion of parents who felt staff supported them in their choice of feeding. The proportion of parents who felt they fully received the support they needed for the choice of feeding method include:

- 78% of n=4735 who wanted and could express milk
- 75% of n=4602 who wanted and could breastfeed
- 76% of n=3507 who wanted to feed their baby formula milk

(See figure 7, graphs D8, D11, & D12).

©2015 Picker Institute Europe. All Rights Reserved.

Figure 5 - Involvement in care

Proportion of parents who responded positively to questions about being involved in care (D1-D7), by network

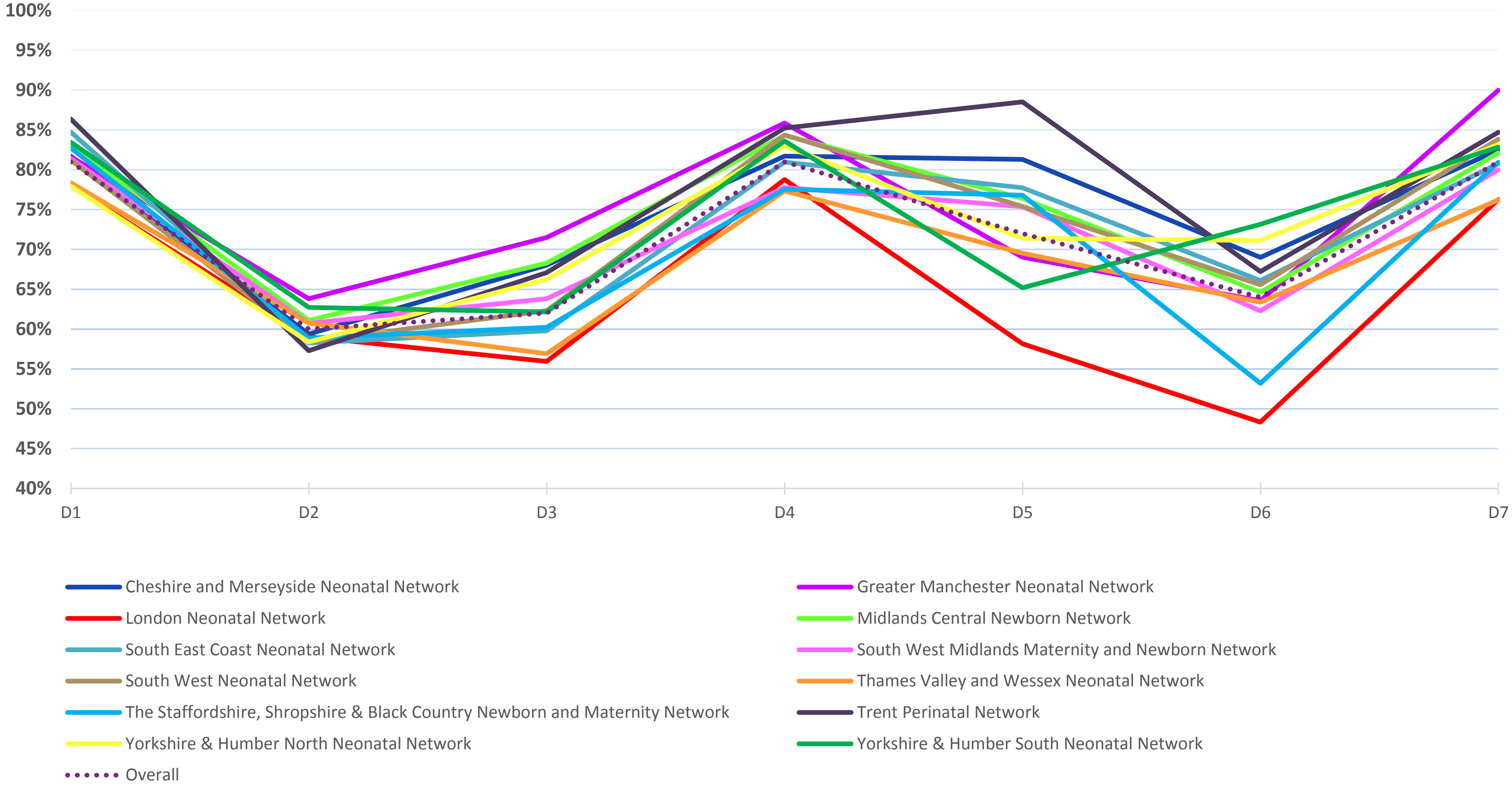

©2015 Picker Institute Europe. All Rights Reserved.

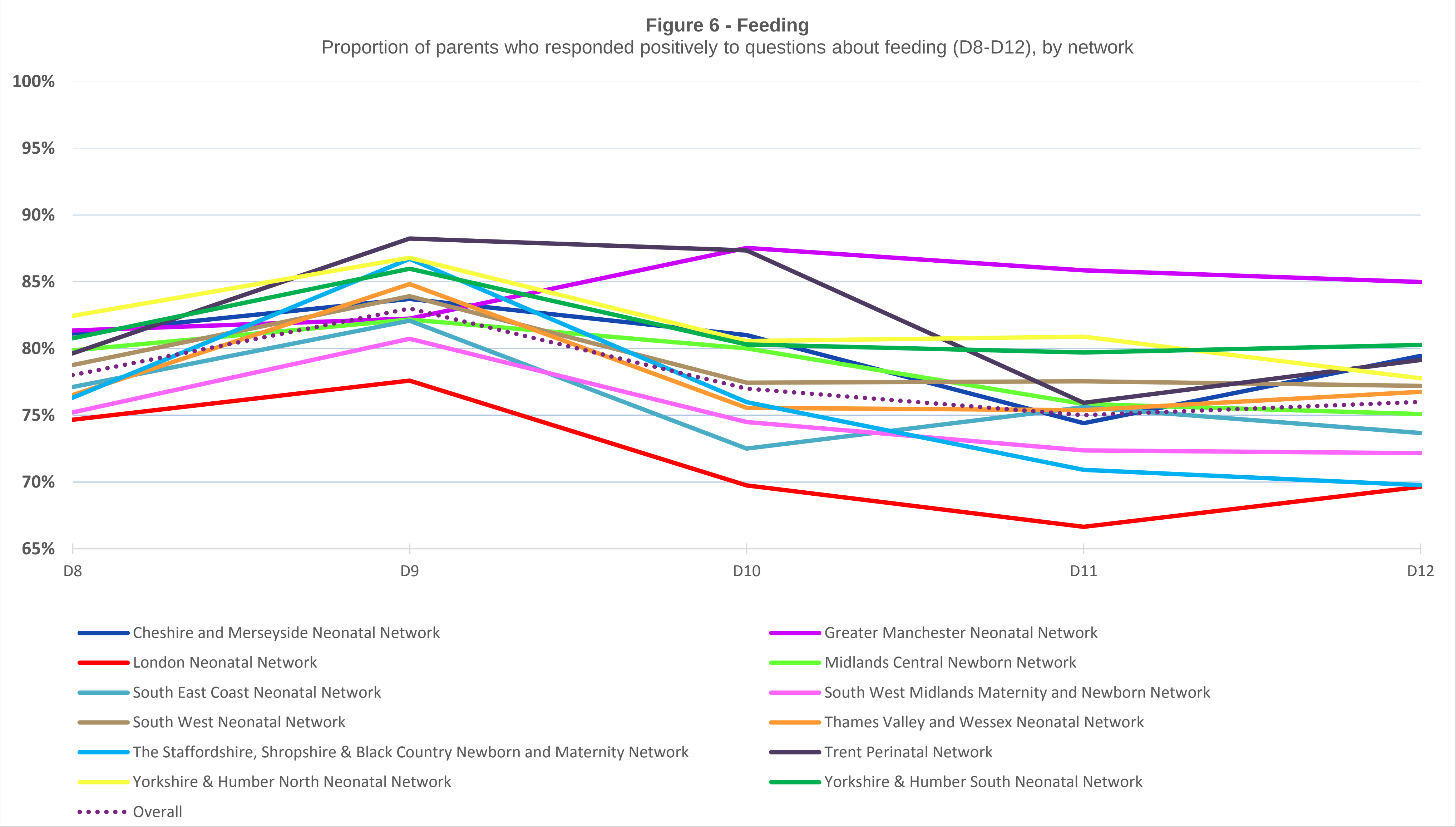

©2015 Picker Institute Europe. All Rights Reserved.

Figure 7: Section D. Graphs

Graph D1. Were you involved as much as you wanted in the day-to-day care of your baby, such as nappy changing and feeding? (Overall n=5852)

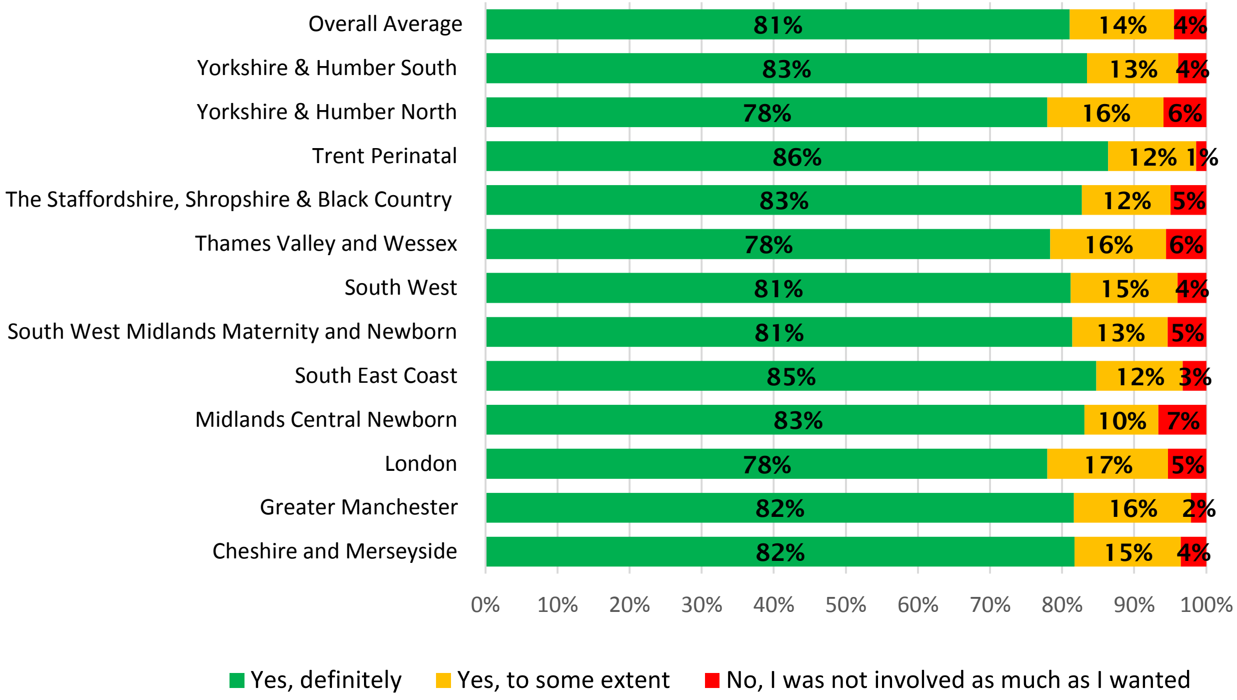

Graph D2. Did you have as much skin- to-skin contact with your baby as you wanted? (Overall n=5106)

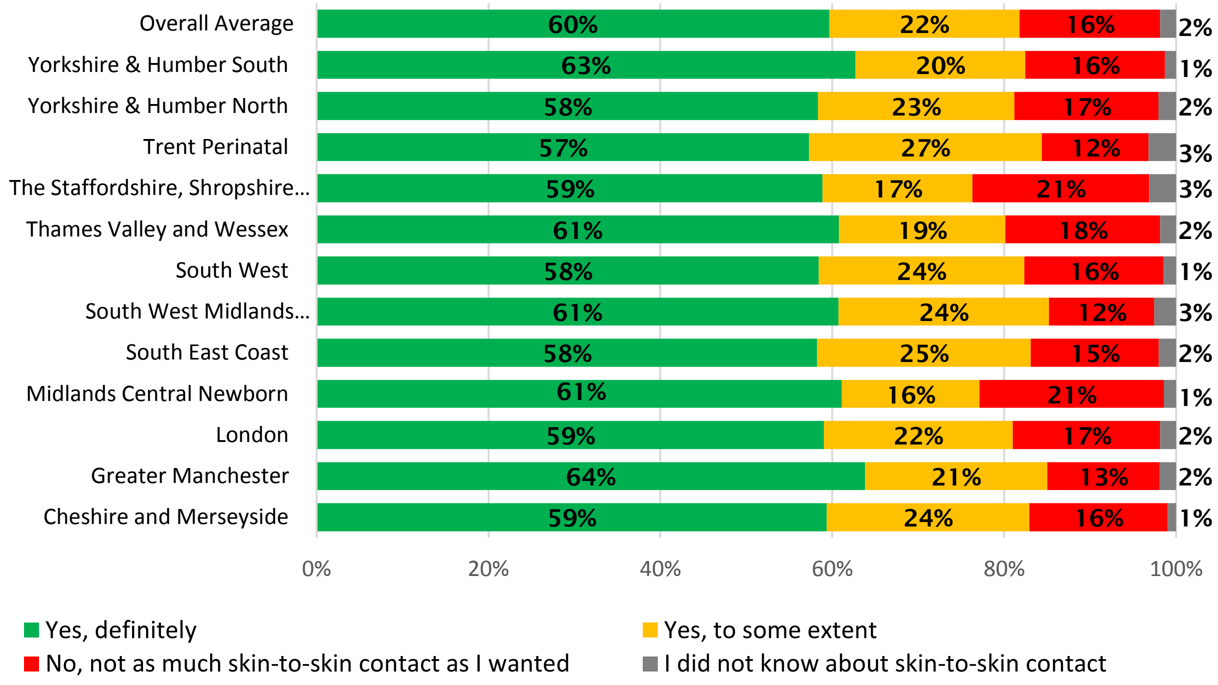

©2015 Picker Institute Europe. All Rights Reserved.

Graph D3. Did the neonatal staff include you in discussions about your baby's care and treatment? (Overall n=5902)

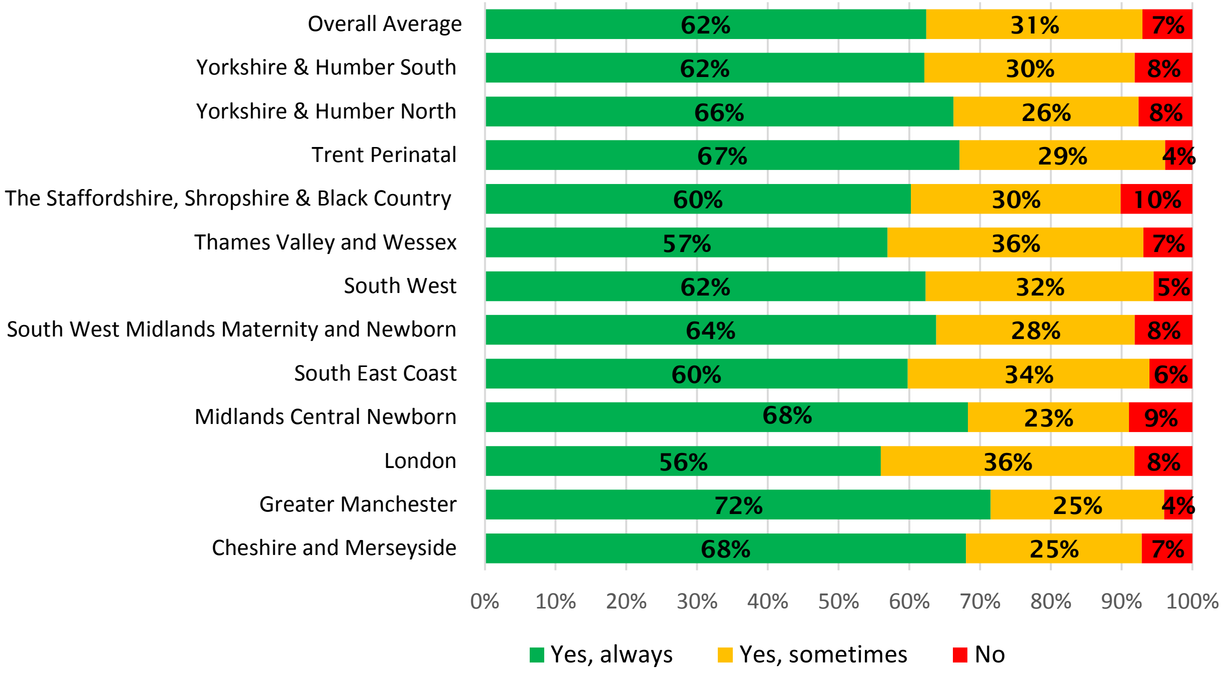

Graph D4. Were you told about any changes in your baby's condition or care? (Overall n=5833)

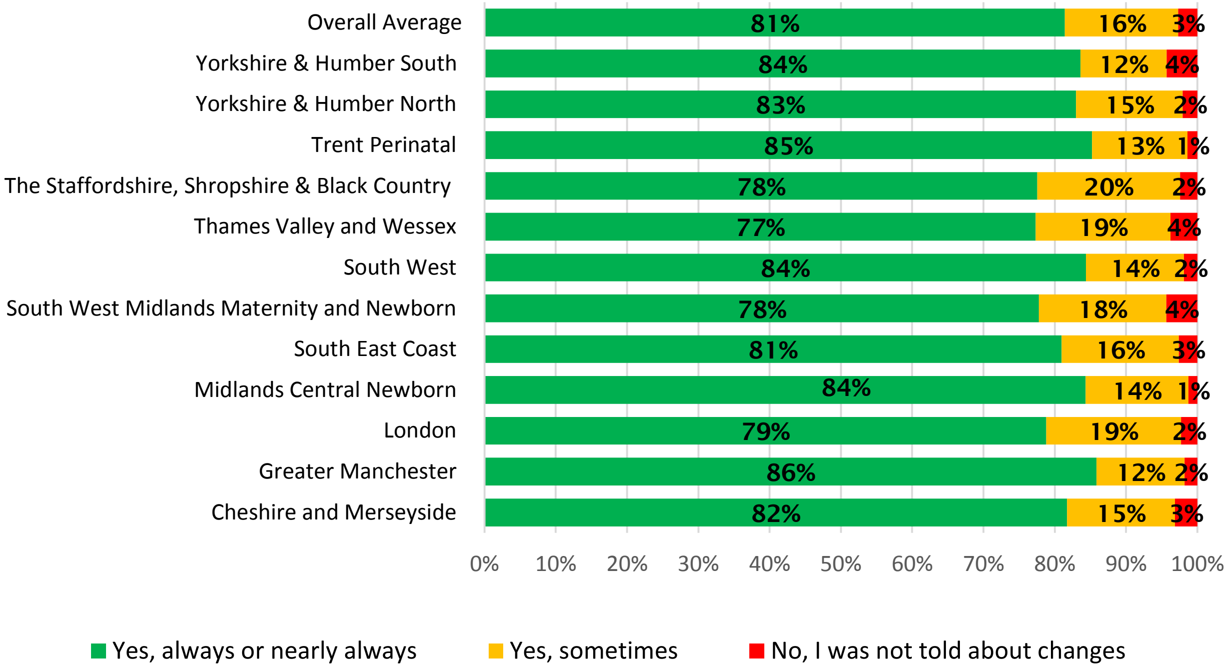

©2015 Picker Institute Europe. All Rights Reserved.

Graph D5. When a ward round was taking place, were you allowed to be present when your baby was being discussed? (Overall n= 5037)

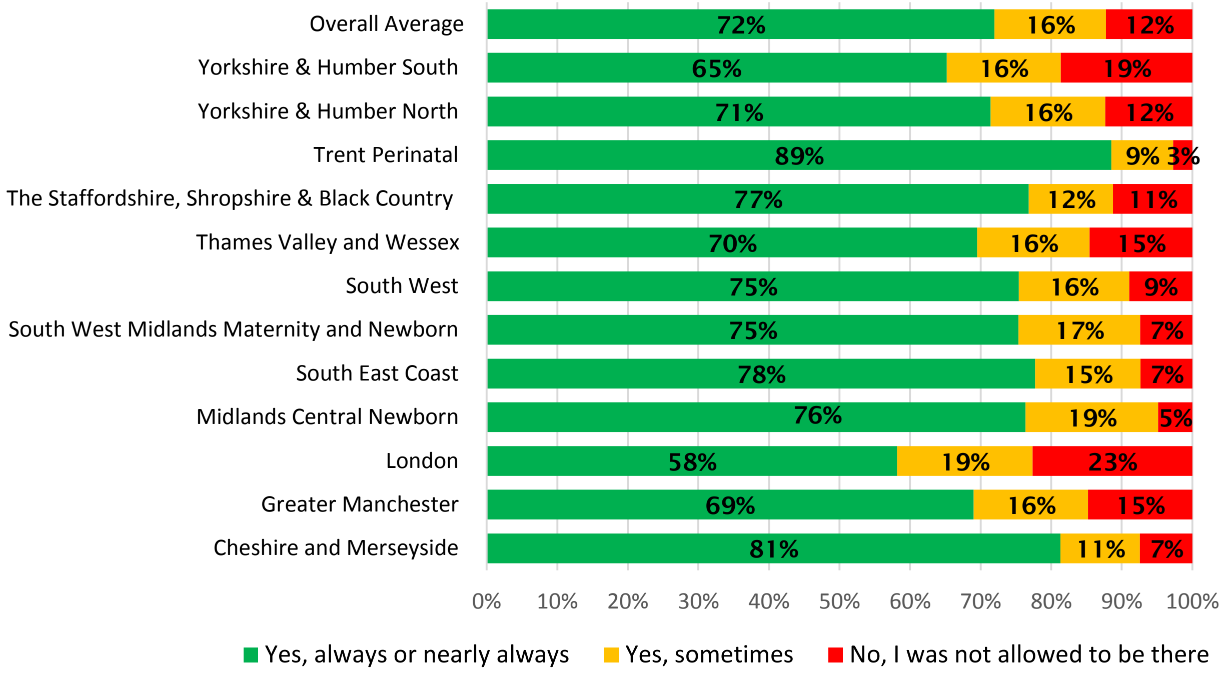

Graph D6. Where possible, did staff arrange your baby's care (such as weighing, bathing) to fit in with your usual visiting times? (Overall n=5833)

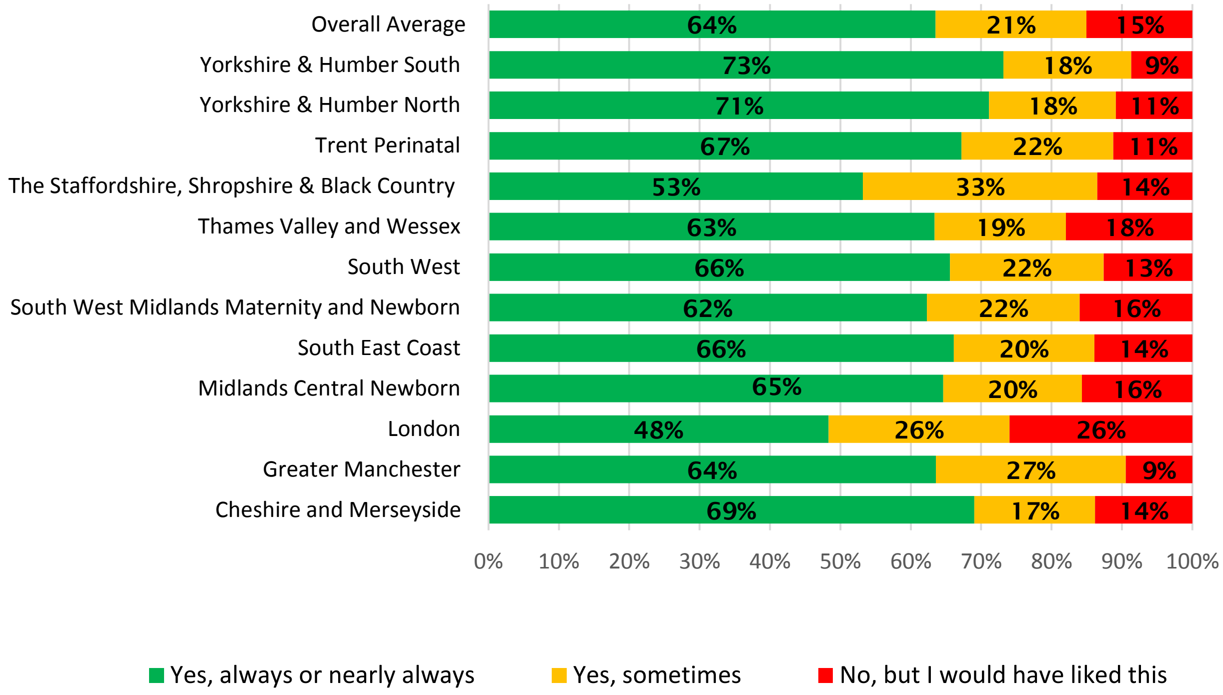

©2015 Picker Institute Europe. All Rights Reserved.

Graph D7. Overall, did staff help you feel confident in caring for your baby? (Overall n=5876)

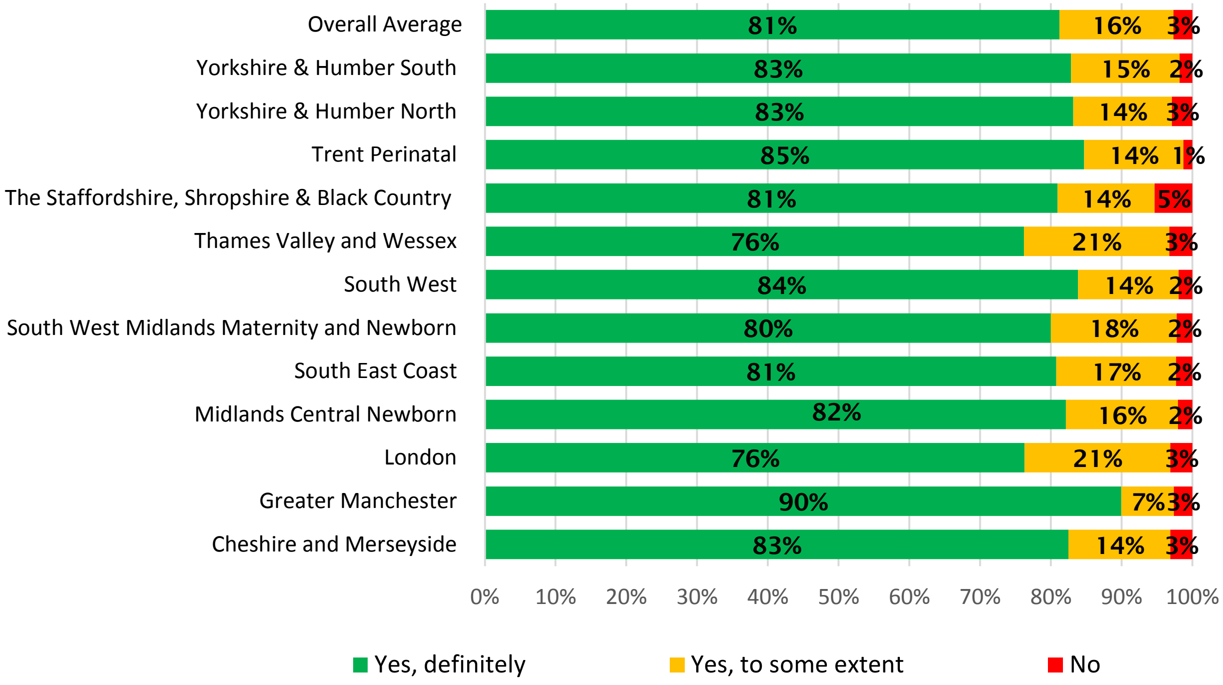

Graph D8. If you wanted to express breast milk for your baby, were you given the support you needed from neonatal staff? (Overall n=4735)

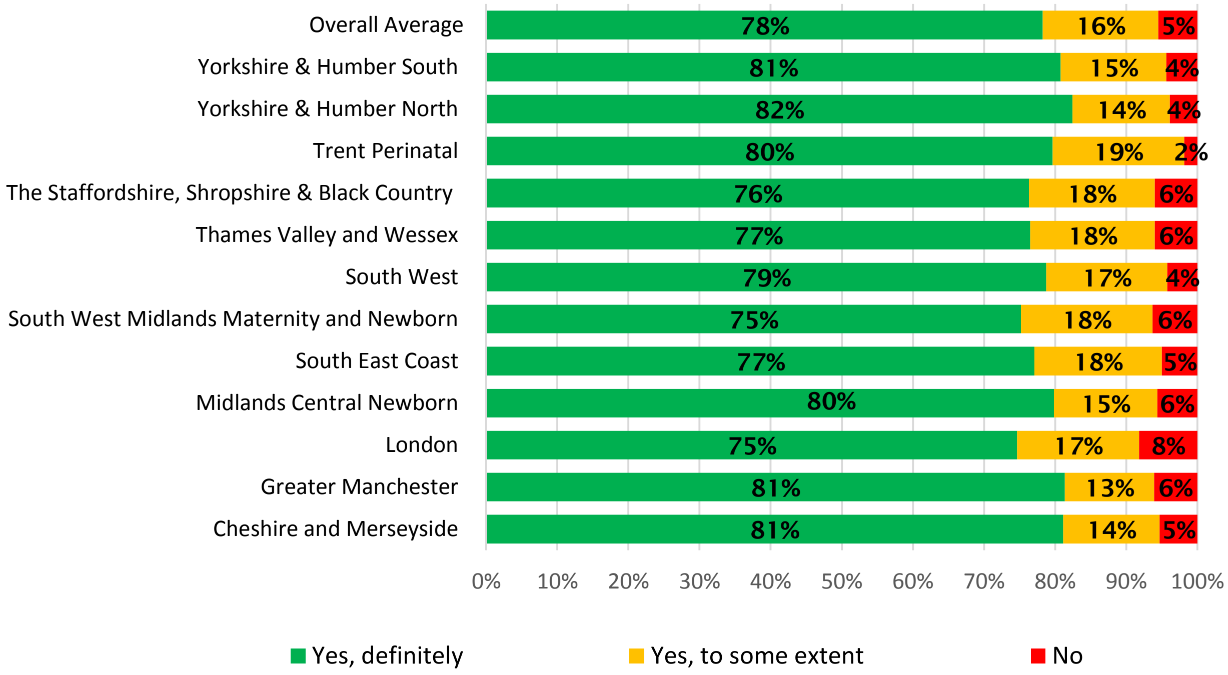

©2015 Picker Institute Europe. All Rights Reserved.

Graph D9. When you were in the neonatal unit, were you given the feeding equipment you needed for expressing, such as a breast pump and sterilisation equipment? (Overall n=4721)

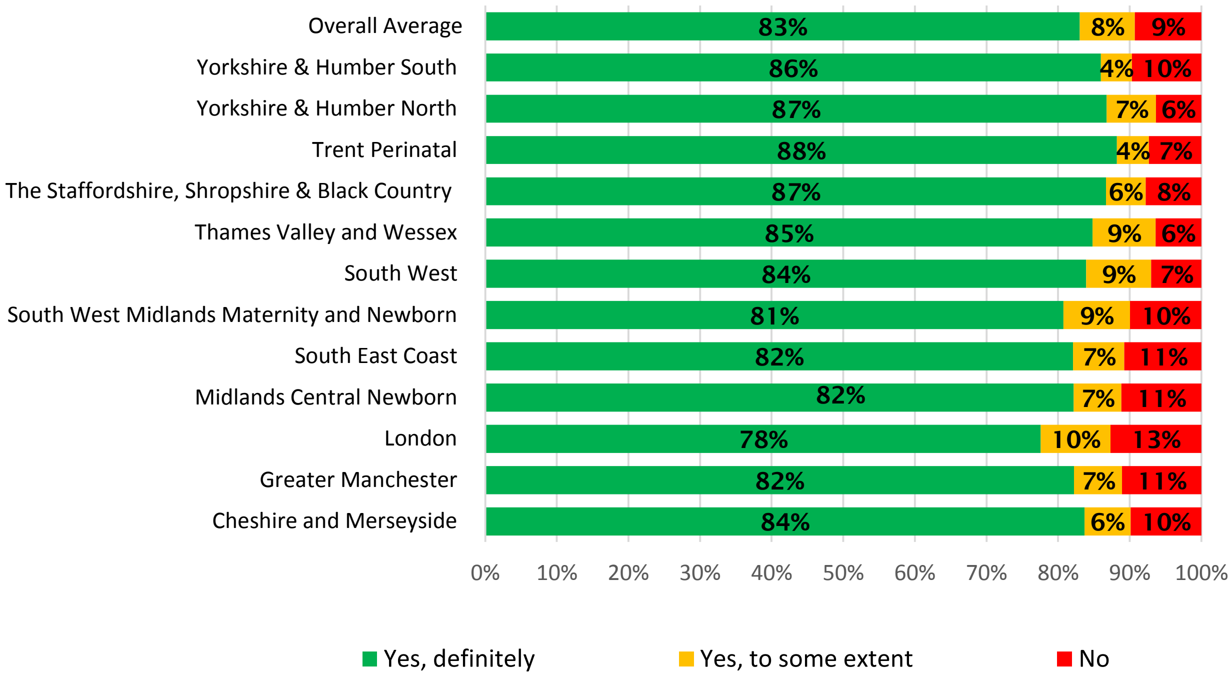

Graph D10. Were you given enough privacy in the neonatal unit for expressing milk and/or breastfeeding your baby? (Overall n=4682)

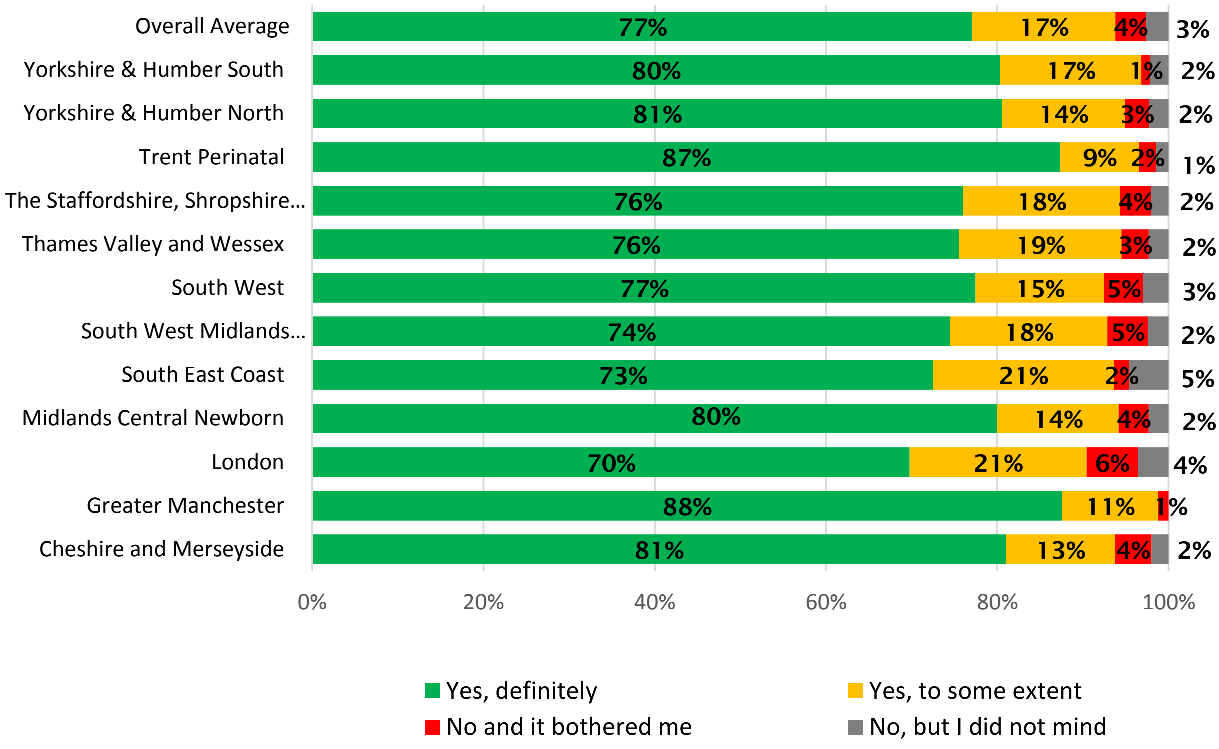

©2015 Picker Institute Europe. All Rights Reserved.

Graph D11. If you wanted to breastfeed your baby, were you given enough support to do this from neonatal staff? (Overall n=4602)

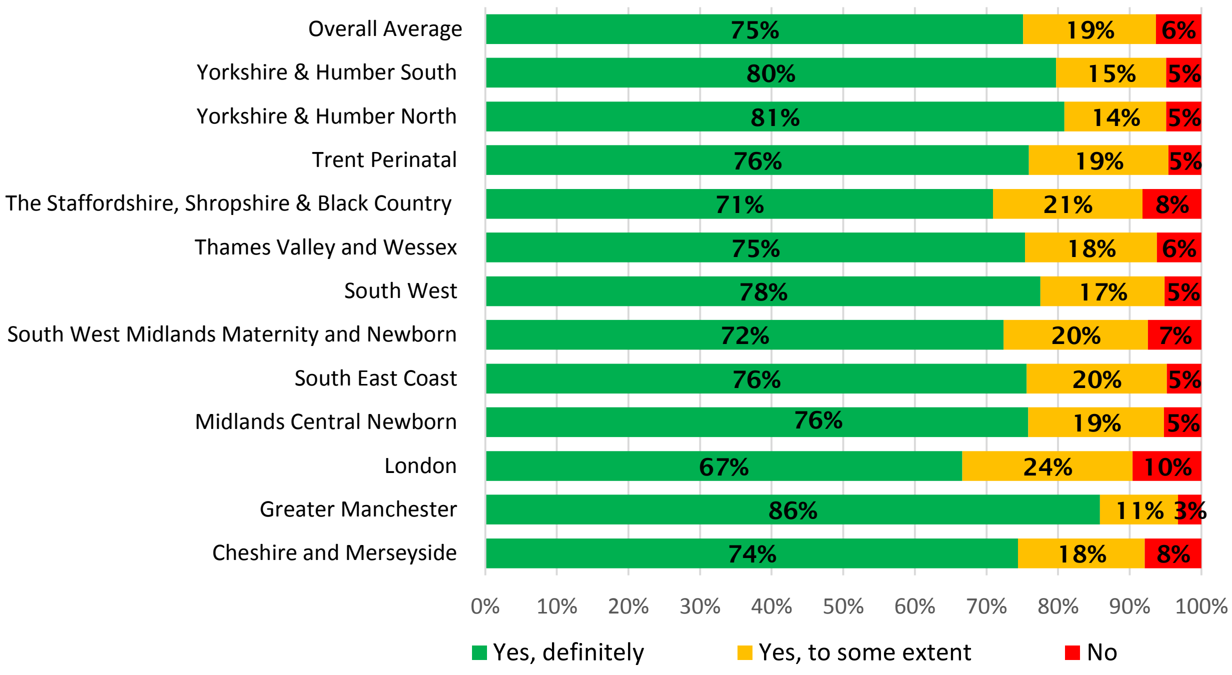

Graph D12. If you fed your baby formula milk, were you given enough support to do this from neonatal staff?

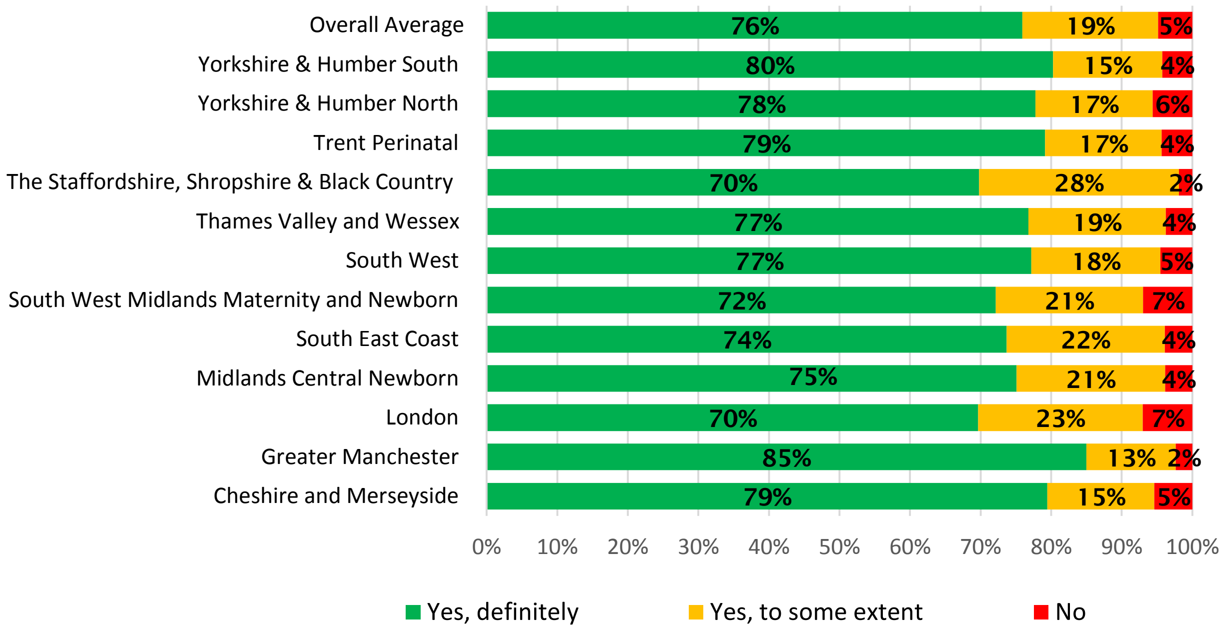

E. Environment and facilities

Parents were asked about the facilities and environment of the neonatal unit. Of those respondents who visited their baby, over 90% across all networks felt they were able to visit their baby on the unit as much as they wanted to, with the exception of the London network (86%, n=810) (figure 8, graph E5). There was a similar trend across networks regarding the environment. For most networks, more than two thirds of parents felt they were given enough privacy when discussing their baby's care with staff on the neonatal unit (68%, n=4026) (figure 8, graph E1), and a large proportion of parents felt there was definitely adequate security on the neonatal unit (80%, n=4708) (figure 8, graph E3). That said, apart from London and Greater Manchester networks, approximately a third of parents stated there was not always enough room to sit alongside their baby's cot in the unit (32%, n=1886) (figure 8, graph E2). Further, the proportion of parents who were offered accommodation by the hospital who wanted to be close to their baby varied across networks (see figure 8, graph E4).

Figure 8: Section E. Graphs

Graph E1. Were you given enough privacy when discussing your baby's care on the neonatal unit with staff? (Overall n=5502)

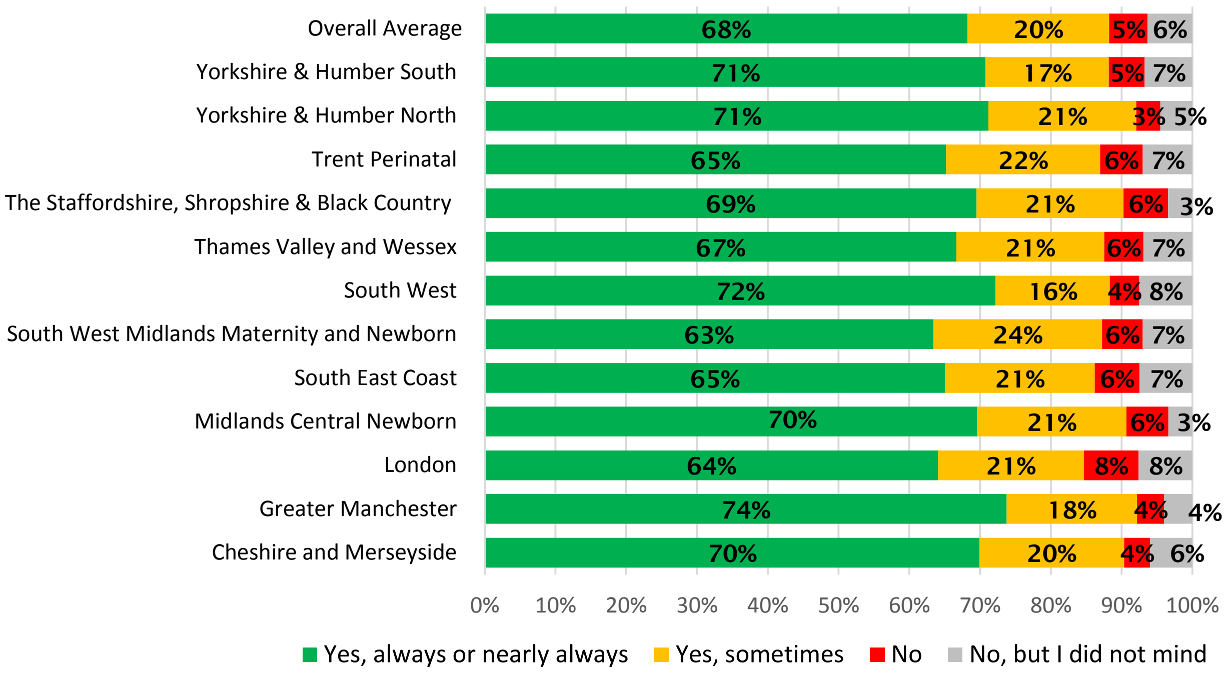

©2015 Picker Institute Europe. All Rights Reserved.

Graph E2. Was there enough space for you to sit alongside your baby's cot in the unit? (Overall n=5875)

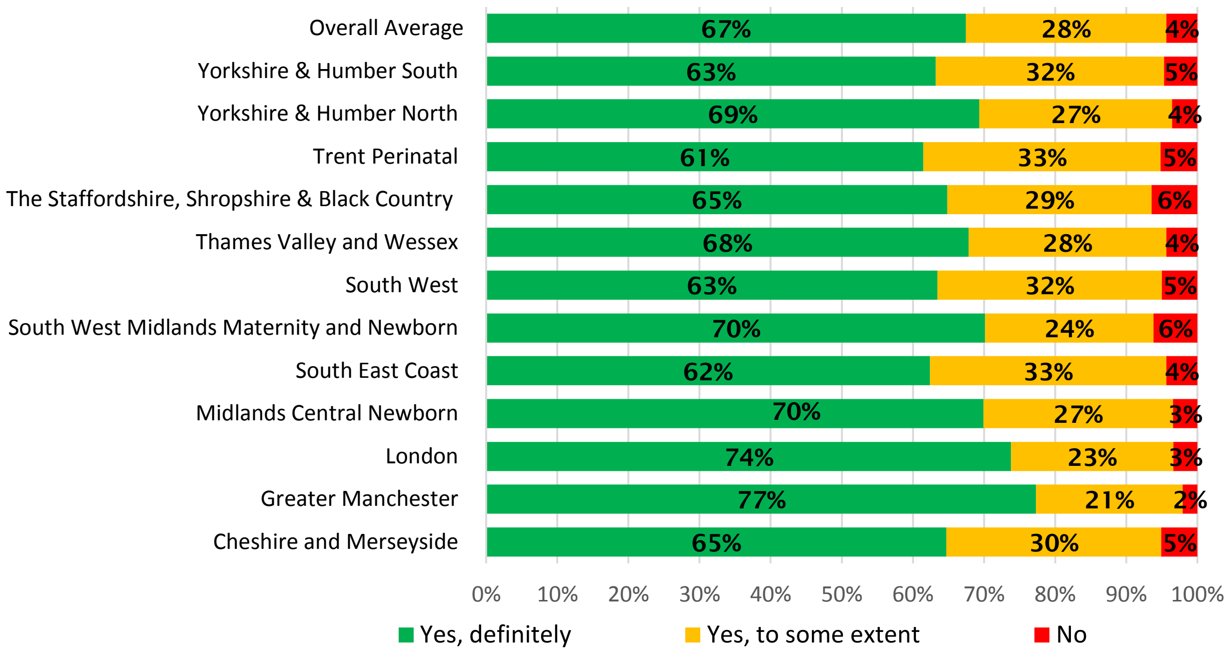

Graph E3. In your opinion, was there adequate security on the neonatal unit? (Overall n=5894)

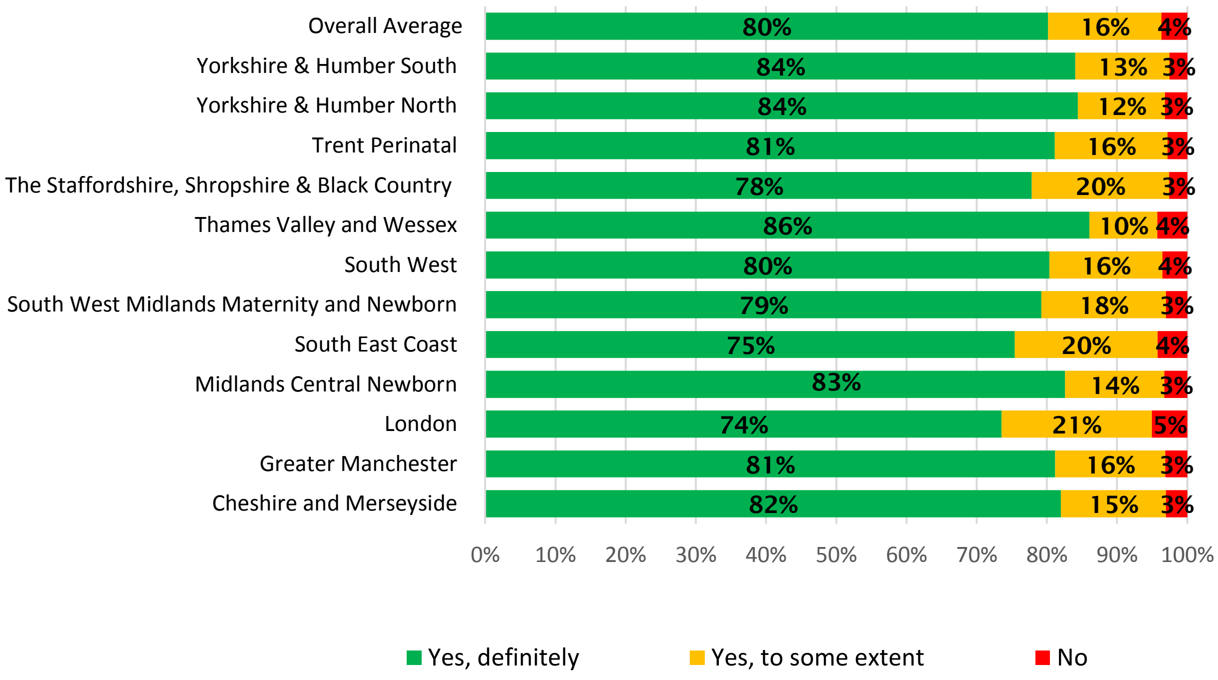

©2015 Picker Institute Europe. All Rights Reserved.

Graph E4. If you wanted to stay overnight to be close to your baby, did the hospital offer you accommodation? (Overall n=4538)

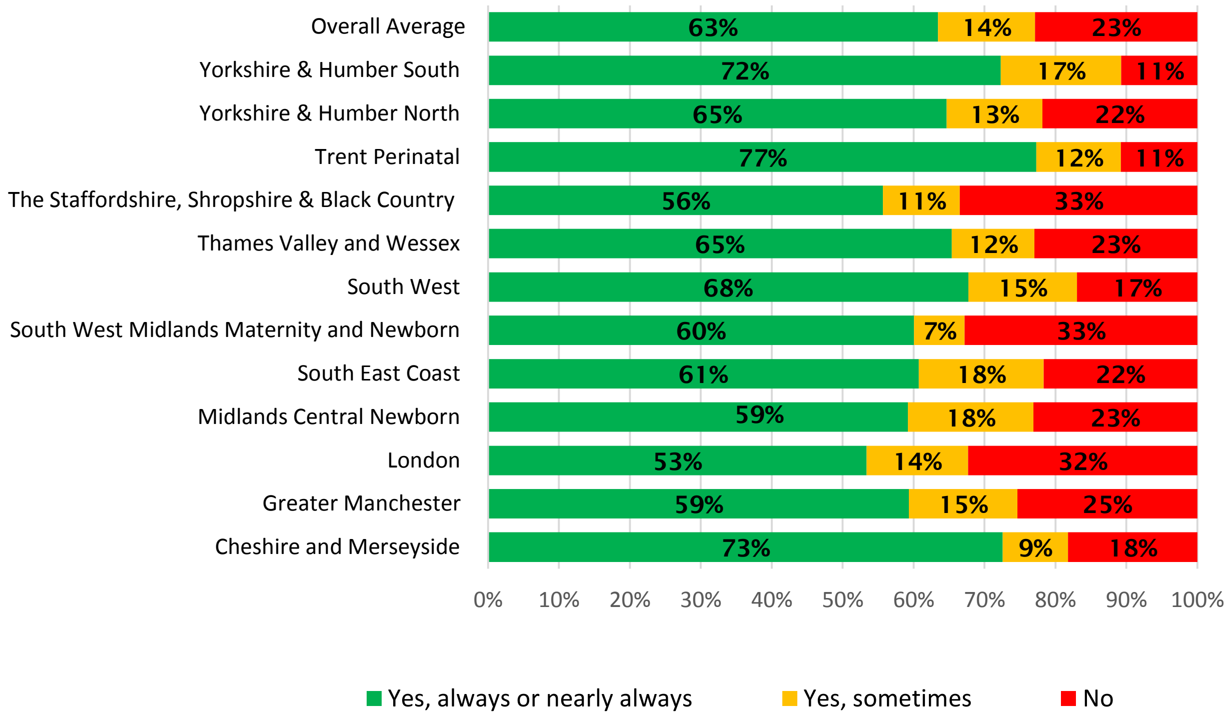

Graph E5. Were you able to visit your baby on the unit as much as you wanted to? (Overall n=5840)

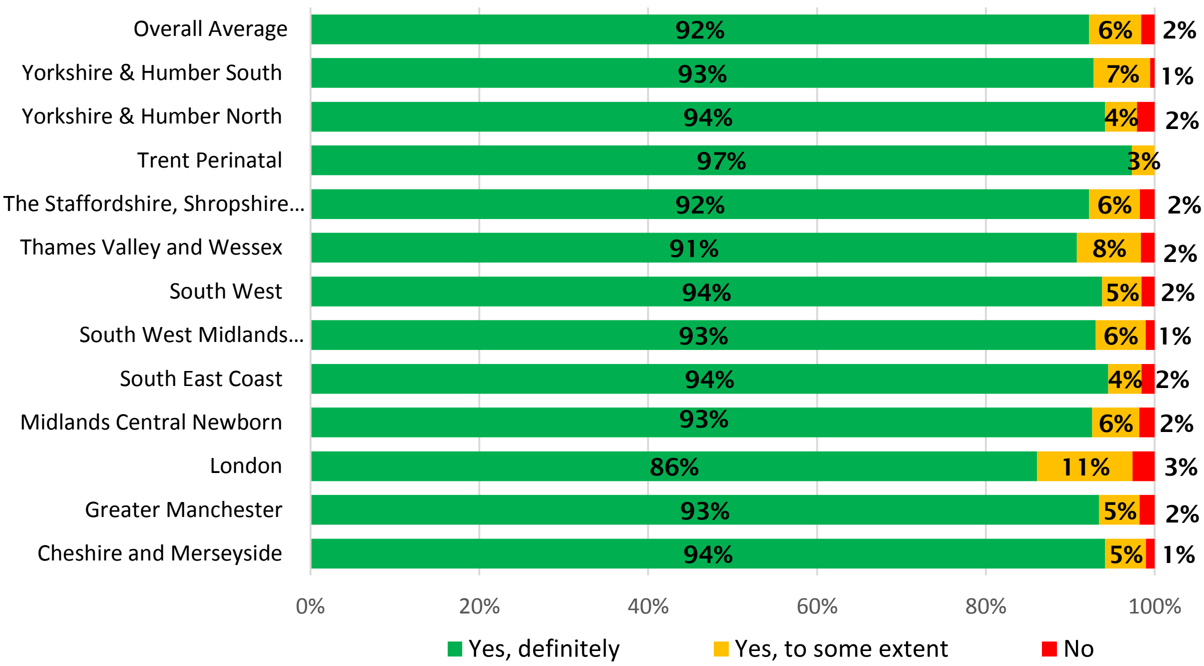

©2015 Picker Institute Europe. All Rights Reserved.

F. Information and support for parents

Overall, the majority of parents from all networks felt they always or sometimes received answers they could understand to their questions about their baby's condition and treatment (see figure 9, graph F1). That said, there is room for improvement for all networks to provide written information to assist parents with understanding their baby's condition and treatment. The London, South East Coast, and Staffordshire, Shropshire and Black Country networks, have the most room for improvement where 42% of parents stated they did not receive any or enough written information (see figure 9, graph F2).

Almost three quarters of parents (71%, n=3336) who wanted or needed it, did not always receive information about help they could get with expenses related to their baby's stay in the neonatal unit, or about support groups such as Bliss (64%, n=3292). Parents from South West Midlands Maternity and Newborn (17%, n=45), and South East Coast networks (19%, n=85) were least likely to receive information about expenses, compared to other networks (see figure 9, graph F5). Parents from Greater Manchester were slightly less likely than parents from other networks to state they received information about support groups (30%, n=94) (see figure 9, graph F6).

Figure 9. Section F. Graphs

Graph F1. If you asked questions about your baby's condition and treatment, did you get answers you could understand? (Overall n=5898)

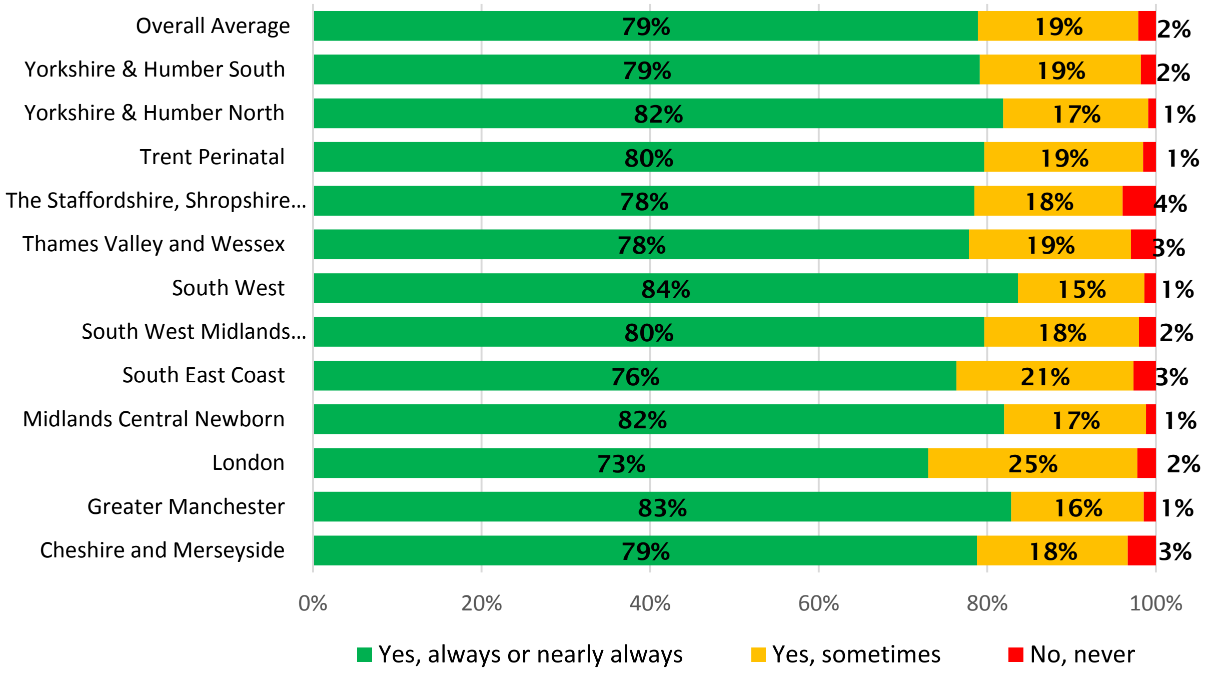

©2015 Picker Institute Europe. All Rights Reserved.

Graph F2. Were you given enough written information to help you understand your baby's condition and treatment? (Overall n=5251)

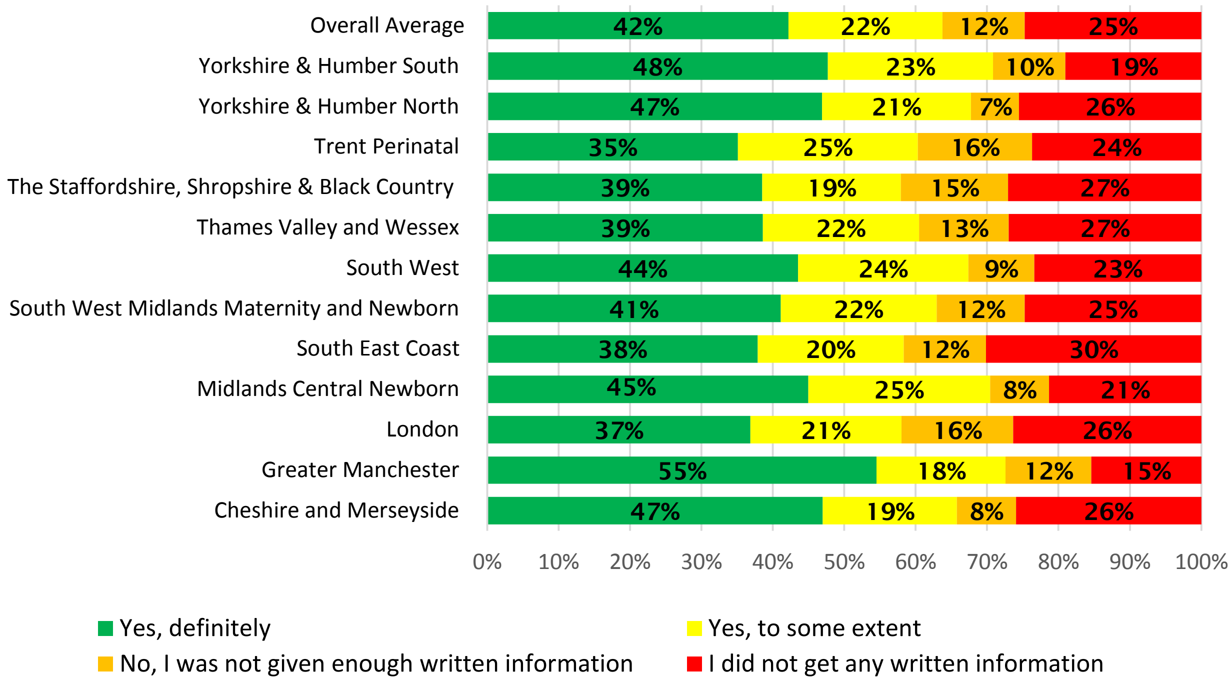

Graph F3. Did you have an opportunity to go through your baby's medical notes with staff while they were in the neonatal unit?

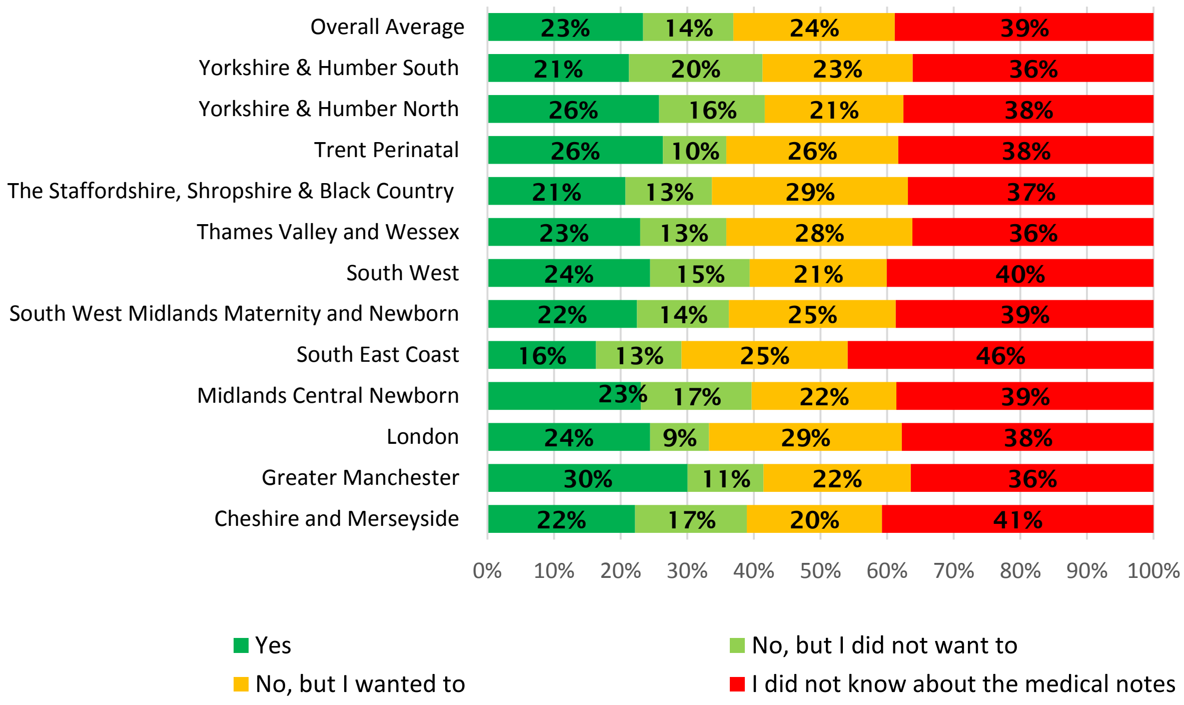

©2015 Picker Institute Europe. All Rights Reserved.

Graph F4. Were you offered emotional support or counselling services from neonatal unit staff? (Overall n=5856)

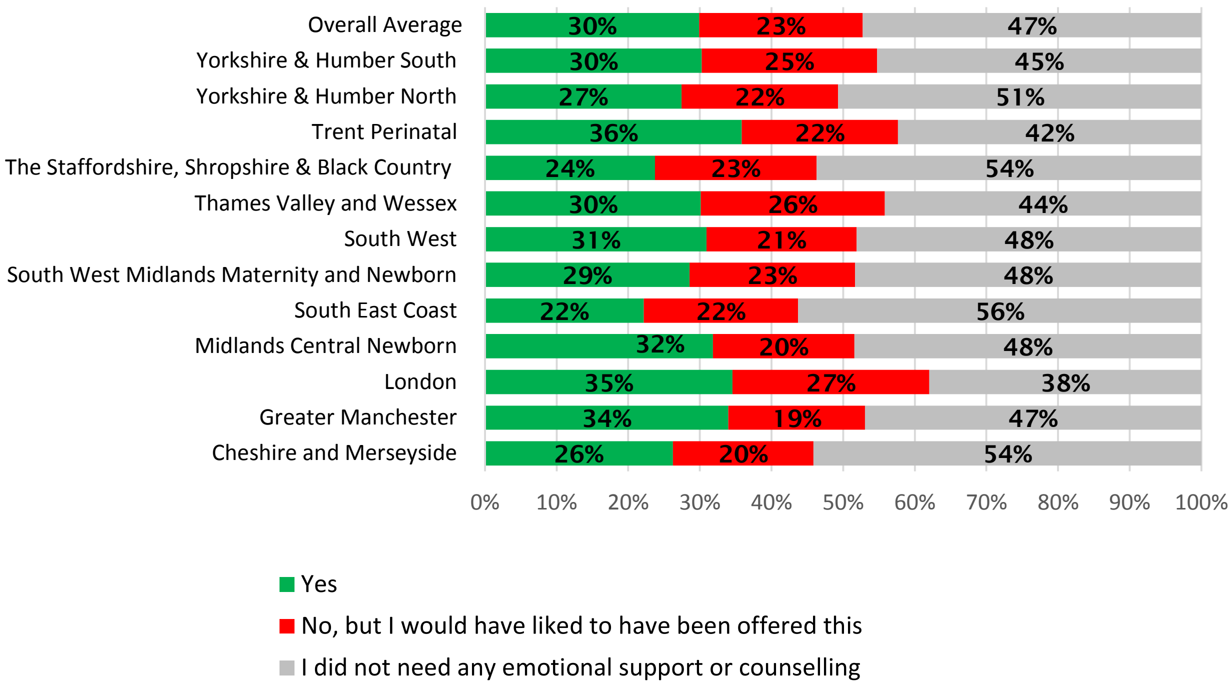

Graph F5. Were you given enough information about help you could get with expenses related to your baby's stay in the neonatal unit? (Overall n=4709)

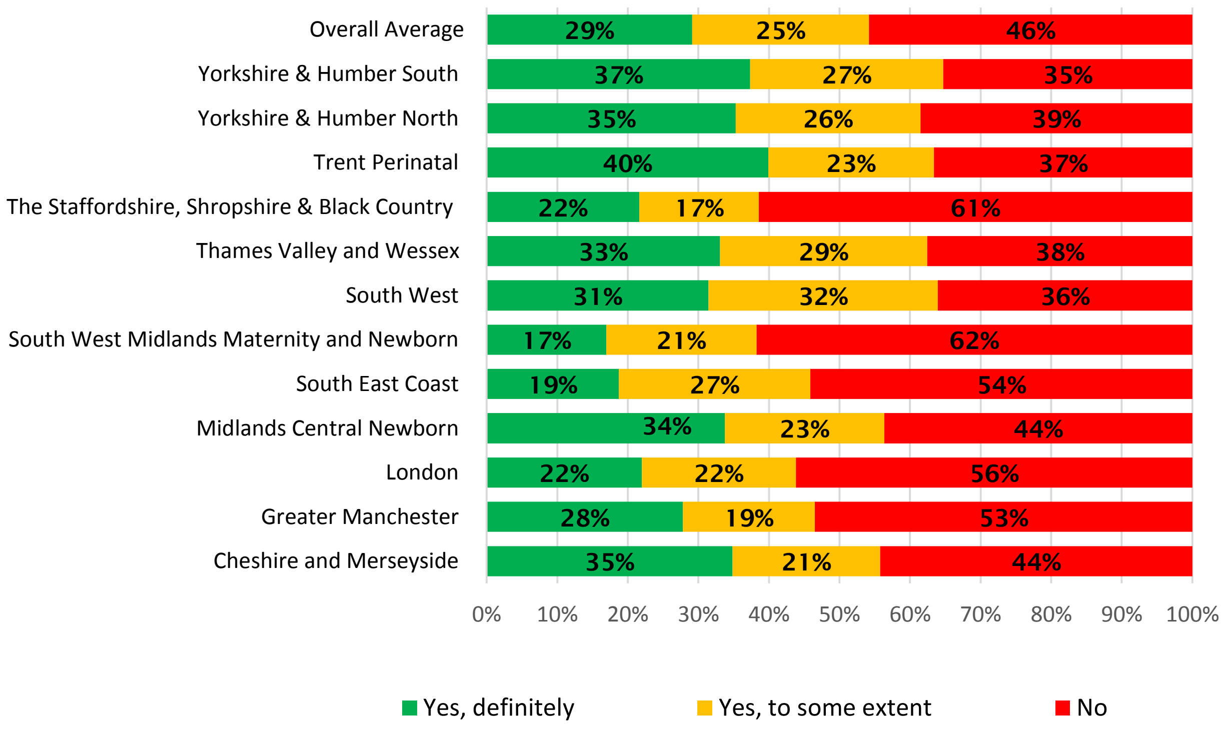

©2015 Picker Institute Europe. All Rights Reserved.

Graph F6. Did staff give you any information about parent support groups, such as Bliss or other local groups? (Overall n=5153)

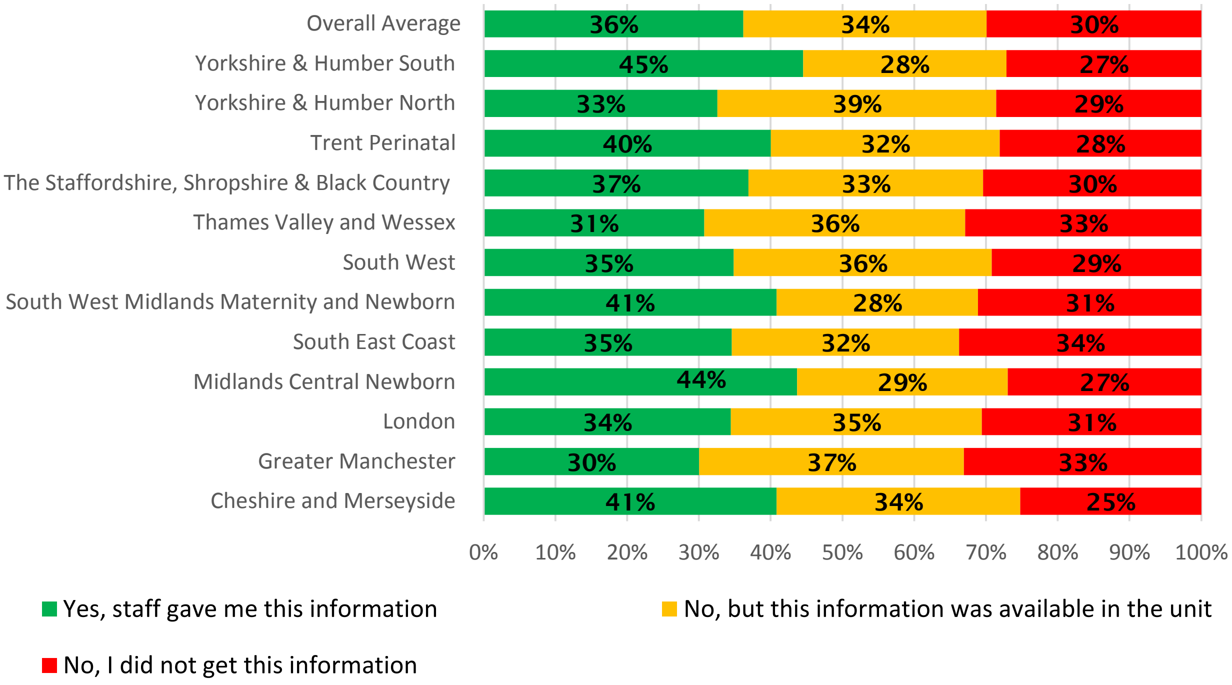

©2015 Picker Institute Europe. All Rights Reserved.

G. Leaving the neonatal unit

Parents were asked questions about leaving the neonatal unit when their baby was discharged. Overall, the majority of parents who's baby went home after discharge stated they were offered overnight accommodation at the hospital before they left the neonatal unit (82%, n=2991). However, parents from South West Midlands (61%, n=87) were least likely compared to other networks to state they were offered, but also had the highest proportion who said they did not want or need this (28%, n=42) (figure 10, graph G3).

Overall, three quarters of parents (75%; n=4427) stated they felt prepared for their baby's discharge from the neonatal network, with those from Midlands Central Newborn Network most likely compared to other networks to note they felt prepared (84%, n=282) (see figure 10, graph G4). That said, more than a third of parents said they did not fully receive enough information on what to expect in terms of their baby's progress and recovery (38%, n=2245) (See figure 10, graph G5). .

Finally, overall 76% (n=4452) of parents said it is extremely likely they would recommend the neonatal unit to friends and family if their baby needed similar care or treatment. The London network however has the most room for improvement overall compared to other networks with 69% (n=648) of parents saying it is extremely like they would recommend units in this network. Please refer to figure 10, graph G6 for a full breakdown of this question by network.

Figure 10: Section G. Graphs

Graph G3. Were you offered overnight accommodation with your baby at the hospital before they left the neonatal unit? (Overall n=3629)

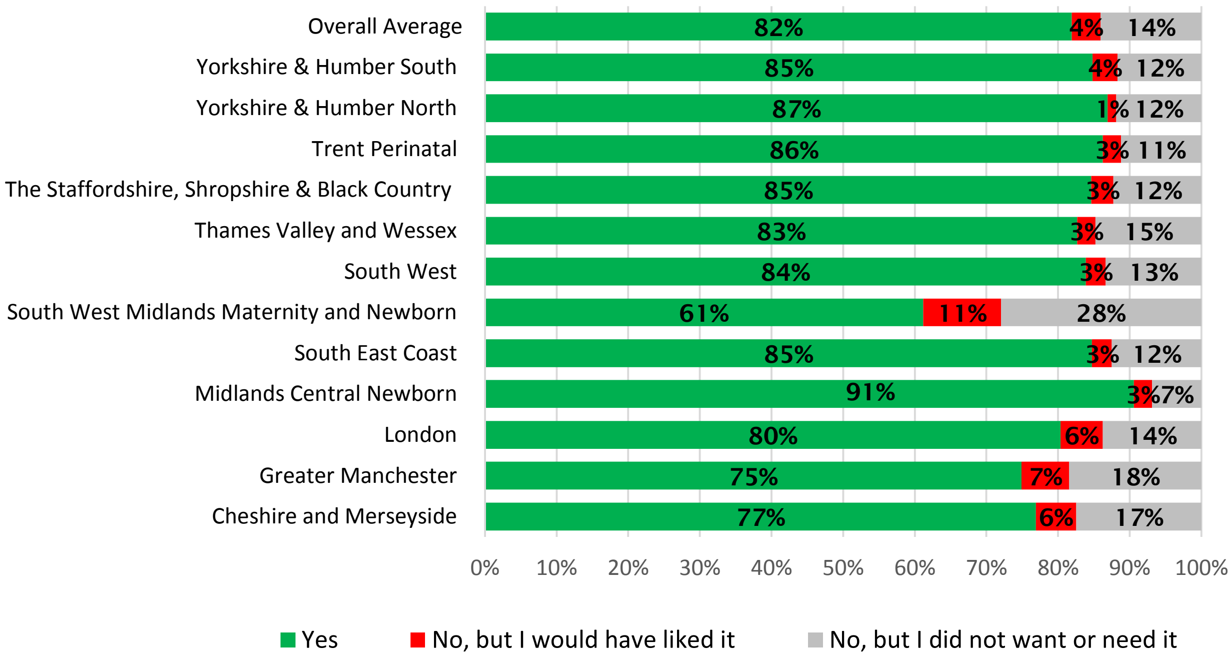

©2015 Picker Institute Europe. All Rights Reserved.

Graph G4. Did you feel prepared for your baby's discharge from neonatal care? (Overall n=5903)

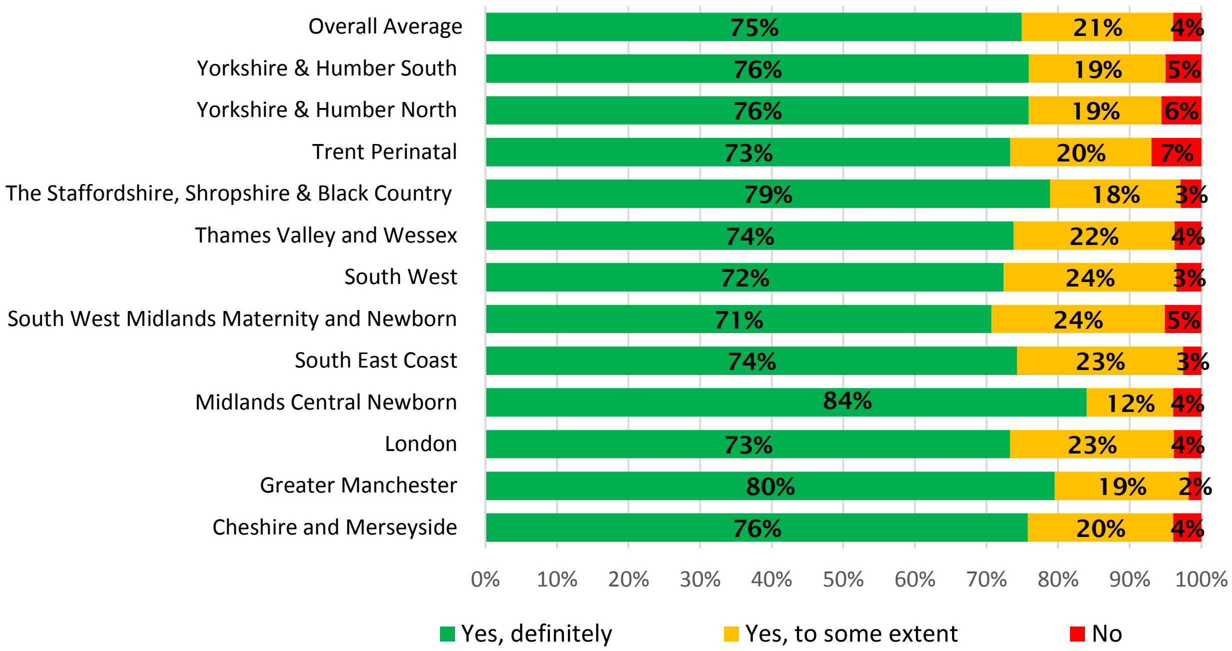

Graph G5. Were you given enough information on what to expect in terms of your baby's progress and recovery? (Overall n=5903)

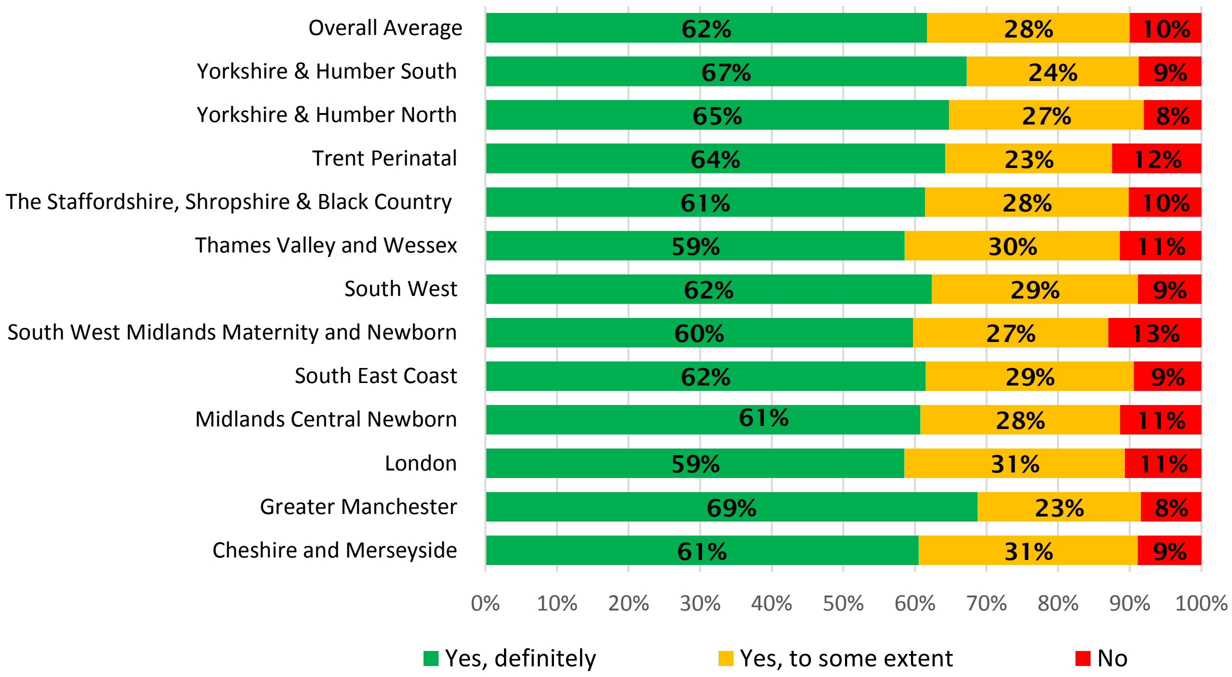

©2015 Picker Institute Europe. All Rights Reserved.

Graph G6. How likely are you to recommend this neonatal unit to friends and family, if their baby needed similar care or treatment? (Overall n=5153)

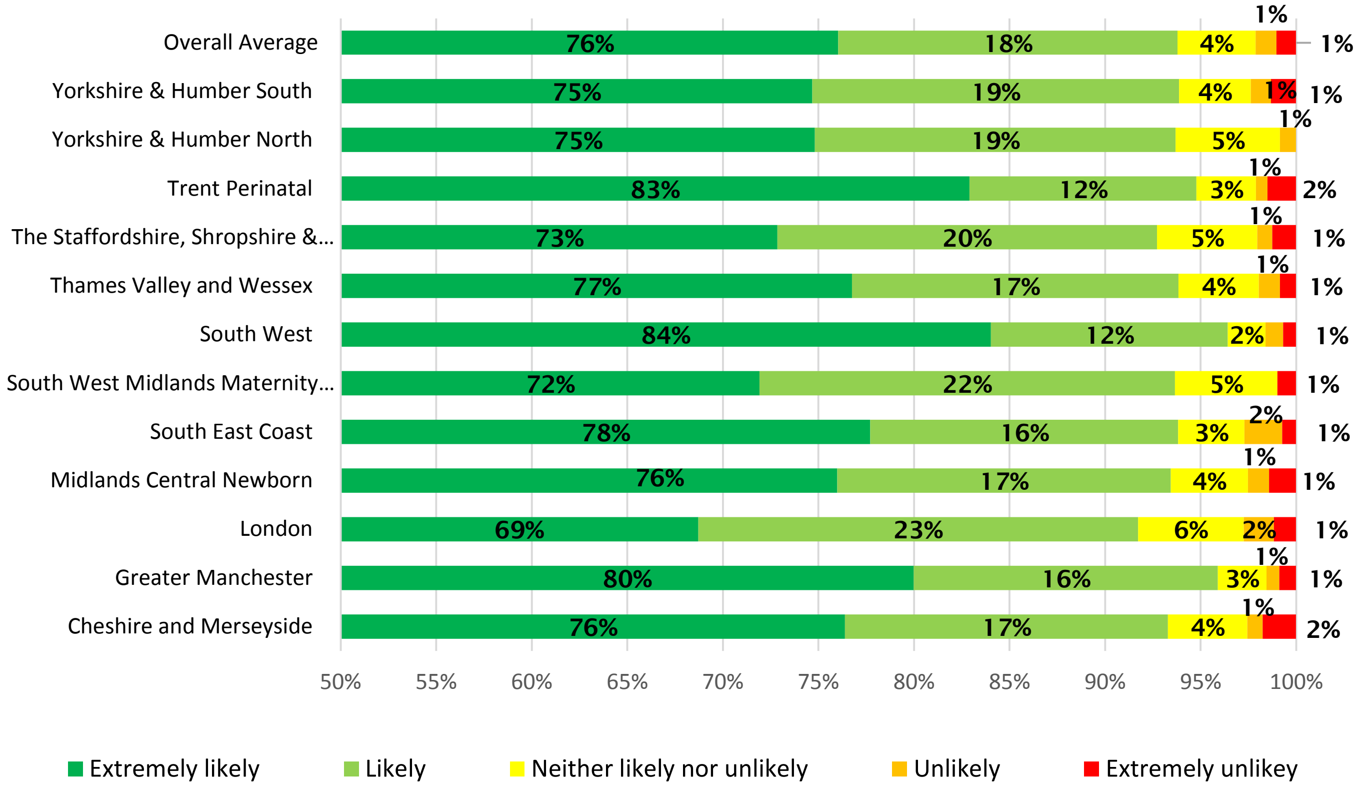

©2015 Picker Institute Europe. All Rights Reserved.

Section Five

Overall Results by Unit Type

Overview by section

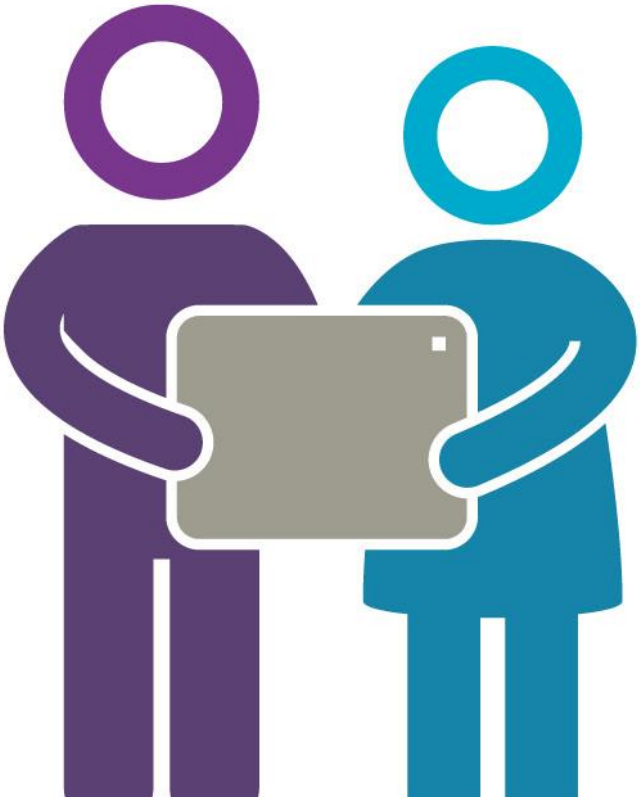

Overall Results by Unit Type

This section shows the overall results for all participating neonatal units by unit type (special care baby units; local neonatal units; neonatal intensive care units) and a comparison against the overall average for all participating units. The results are presented in graphical format only by the sections in the questionnaire. Table 6 below presents units included in the breakdowns by unit type.

Table 6. List of neonatal units per unit type

Unit Type	Unit Name
Special Care Baby Unit	YEOVIL DISTRICT HOSPITAL
	THE ROYAL FREE HOSPITAL
	EALING HOSPITAL
	SCARBOROUGH HOSPITAL
	KING GEORGE HOSPITAL
	QUEEN ELIZABETH HOSPITAL WOOLWICH
	QUEEN'S HOSPITAL, BURTON UPON TRENT
	GEORGE ELIOT HOSPITAL
	BASSETLAW DISTRICT GENERAL HOSPITAL
	GOOD HOPE HOSPITAL
	HORTON GENERAL HOSPITAL
	THE QUEEN ELIZABETH THE QUEEN MOTHER HOSPITAL
	ALEXANDRA HOSPITAL
	CONQUEST HOSPITAL
	DEWSBURY AND DISTRICT HOSPITAL

©2015 Picker Institute Europe. All Rights Reserved.

Table 6. *continued*

Unit Type	Unit Name
Local Neonatal Unit	ST MARY'S HOSPITAL, ISLE OF WIGHT
	WHIPPS CROSS UNIVERSITY HOSPITAL AND NEWHAM GENERAL HOSPITAL
	KINGSTON HOSPITAL
	MUSGROVE PARK HOSPITAL
	WHISTON HOSPITAL
	ALDER HEY HOSPITAL
	YORK HOSPITAL
	SHEFFIELD CHILDREN’S HOSPITAL NEONATAL SURGICAL UNIT
	WEXHAM PARK HOSPITAL
	MILTON KEYNES HOSPITAL
	FRIMLEY PARK HOSPITAL
	ROYAL CORNWALL HOSPITAL
	ROTHERHAM DISTRICT GENERAL HOSPITAL
	CHESTERFIELD ROYAL HOSPITAL
	ROYAL DEVON AND EXETER HOSPITAL
	UNIVERSITY HOSPITAL LEWISHAM
	DIANA PRINCESS OF WALES HOSPITAL
	SCUNTHORPE GENERAL HOSPITAL
	MACCLESFIELD DISTRICT GENERAL HOSPITAL
	COUNTESS OF CHESTER HOSPITAL
	KING'S MILL HOSPITAL
	WYTHENSHAWE HOSPITAL
	THE GREAT WESTERN HOSPITAL
	RUSSELLS HALL HOSPITAL
	NORTHAMPTON GENERAL HOSPITAL
	SALISBURY DISTRICT HOSPITAL
	DONCASTER ROYAL INFIRMARY
	ROYAL ALBERT EDWARD INFIRMARY
	GLOUCESTERSHIRE ROYAL HOSPITAL
	EAST SURREY HOSPITAL
	NORTHWICK PARK HOSPITAL
	ORMSKIRK & DISTRICT GENERAL HOSPITAL
	NORTH MANCHESTER GENERAL HOSPITAL
	ROYAL OLDHAM HOSPITAL
	WORCESTERSHIRE ROYAL HOSPITAL
	WARRINGTON HOSPITAL
	CALDERDALE ROYAL HOSPITAL
	PINDERFIELDS GENERAL HOSPITAL
	STOKE MANDEVILLE HOSPITAL
	PRINCESS ROYAL HOSPITAL
	ST MARY'S HOSPITAL

©2015 Picker Institute Europe. All Rights Reserved.

Table 6. *continued*

Unit Type	Unit Name
**Neonatal Intensive Care Unit**	THE ROYAL LONDON HOSPITAL
	ST MICHAEL'S HOSPITAL
	BRADFORD ROYAL INFIRMARY MATERNITY UNIT
	ARROWE PARK HOSPITAL
	LIVERPOOL WOMENS HOSPITAL
	QUEEN'S HOSPITAL, ROMFORD
	NOTTINGHAM CITY HOSPITAL
	PRINCESS ANNE HOSPITAL
	THE JESSOP WING, SHEFFIELD
	QUEEN ALEXANDRA HOSPITAL
	ST THOMAS' HOSPITAL
	ST GEORGE'S HOSPITAL
	UNIVERSITY HOSPITAL OF NORTH STAFFORDSHIRE
	DERRIFORD HOSPITAL
	UNIVERSITY HOSPITAL COVENTRY
	BIRMINGHAM WOMEN'S HOSPITAL
	ROYAL BOLTON HOSPITAL
	MEDWAY MARITIME HOSPITAL
	CHELSEA & WESTMINSTER HOSPITAL
	BIRMINGHAM HEARTLANDS HOSPITAL
	LEEDS NEONATAL SERVICE
	UNIVERSITY COLLEGE HOSPITAL
	QUEENS MEDICAL CENTRE
	JOHN RADCLIFFE HOSPITAL
	ST PETER'S HOSPITAL
	SOUTHMEAD HOSPITAL
	WILLIAM HARVEY HOSPITAL
	ST MARY'S HOSPITAL, MANCHESTER
	LEICESTER NEONATAL SERVICE
	ROYAL PRESTON HOSPITAL
	QUEEN CHARLOTTE'S AND CHELSEA HOSPITAL

©2015 Picker Institute Europe. All Rights Reserved.

Figure 11: Graphs by unit type:

A. Before your baby was born

A2. Before your baby was born (i.e. during pregnancy or labour), did a member of staff from the neonatal unit talk to you about what to expect after the birth? (Overall n=1919)

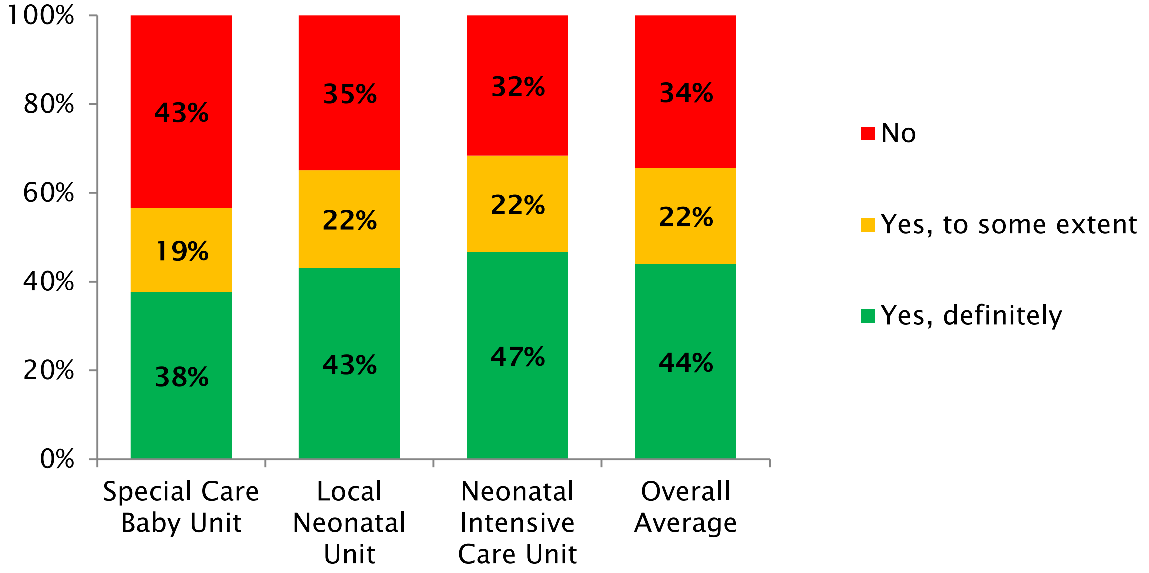

B. Your baby's admission to neonatal care

B2. After you gave birth, were you offered a photograph of your baby? (Overall n=4972)

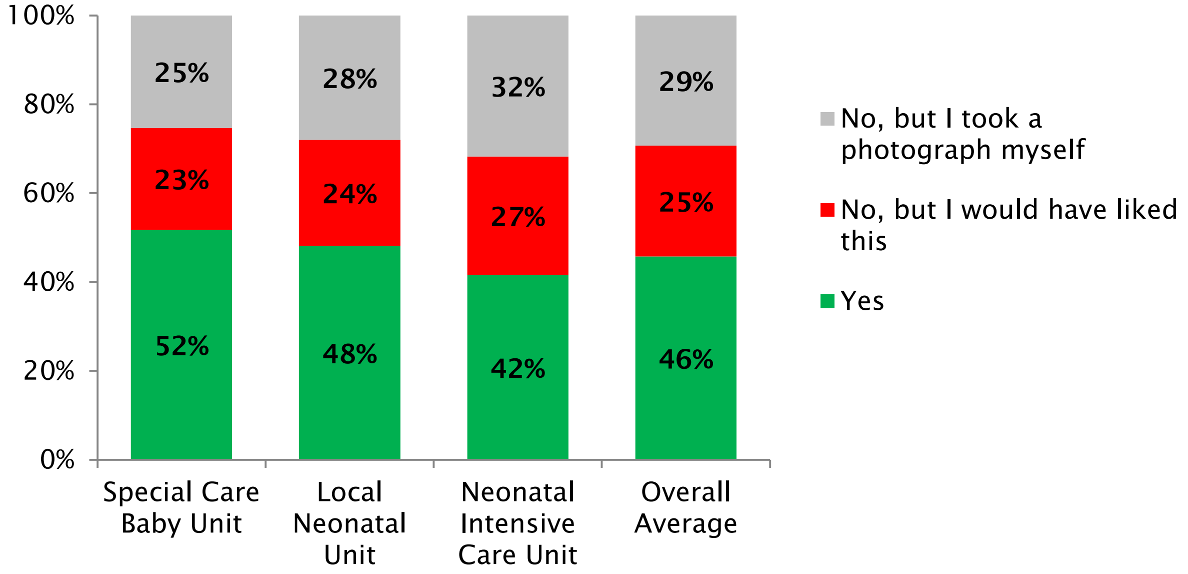

©2015 Picker Institute Europe. All Rights Reserved.

B3. After you gave birth, were you ever cared for in the same ward as mothers who had their baby with them? (Overall n=5167)

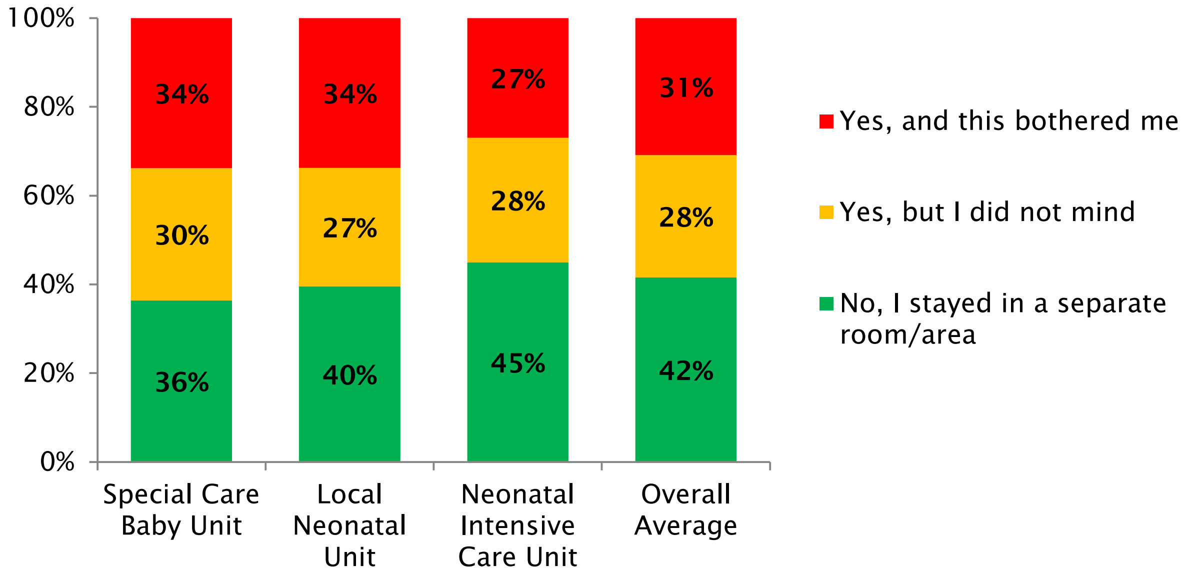

B4. After your baby was admitted to the neonatal unit, were you able to see your baby as soon as you wanted? (Overall n=3858)

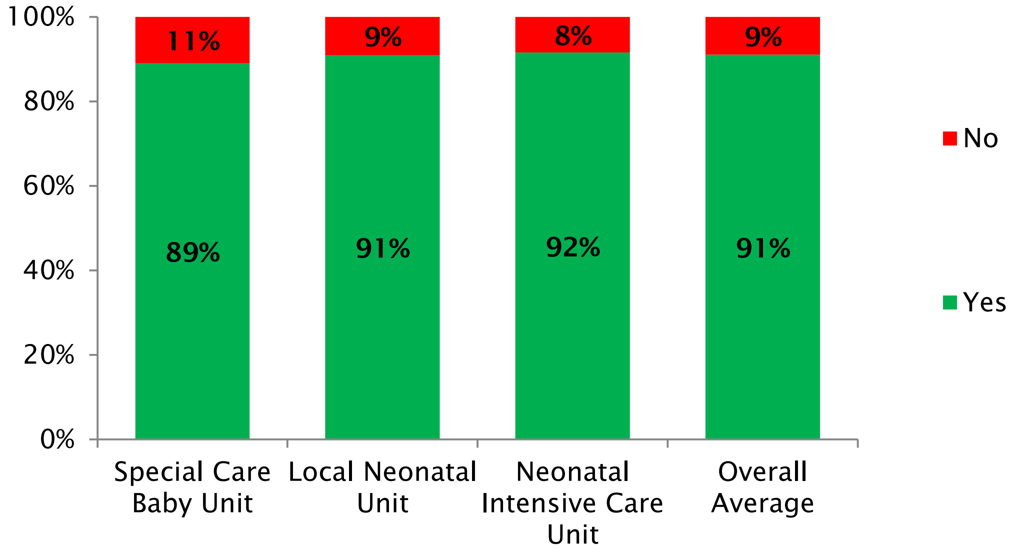

©2015 Picker Institute Europe. All Rights Reserved.

C. Staff on the neonatal unit

C1. When you visited the unit, did the staff caring for your baby introduce themselves to you? (Overall n=5804)

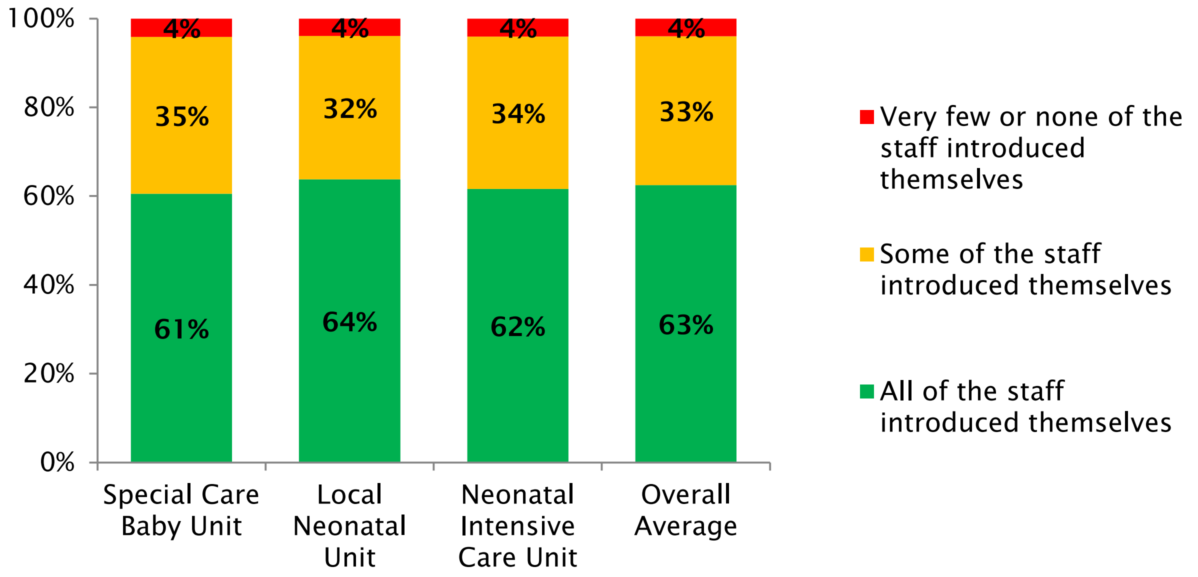

C2. Were you given enough information about the neonatal unit (such as rules, procedures and facilities for parents)? (Overall n=5804)

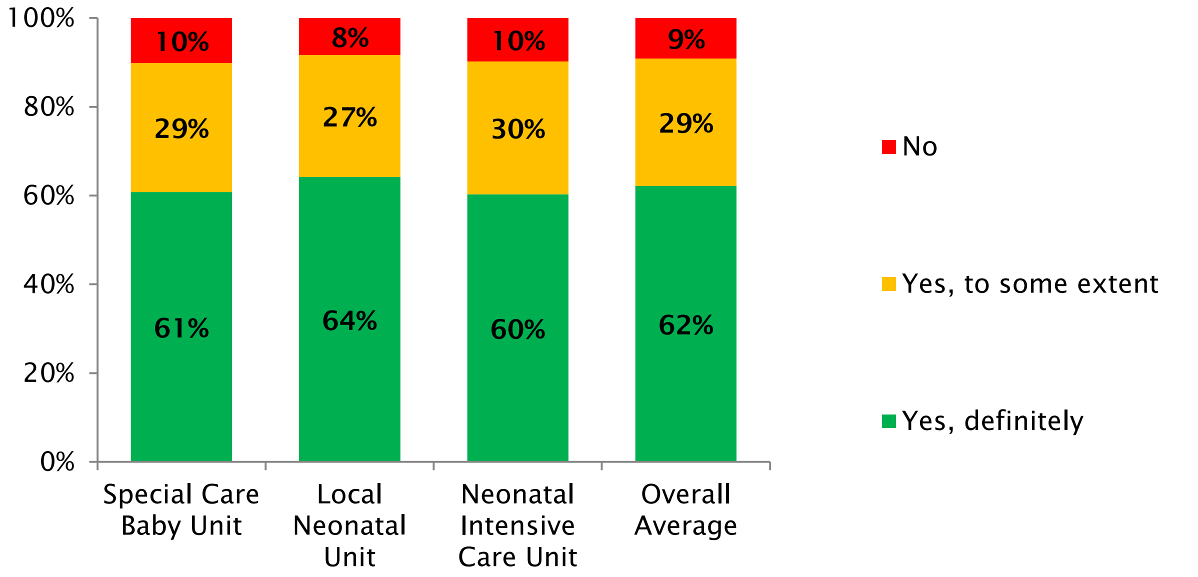

©2015 Picker Institute Europe. All Rights Reserved.

C3. Was the purpose of the machines, monitors and alarms used in the neonatal unit clearly explained to you? (Overall n=5767)

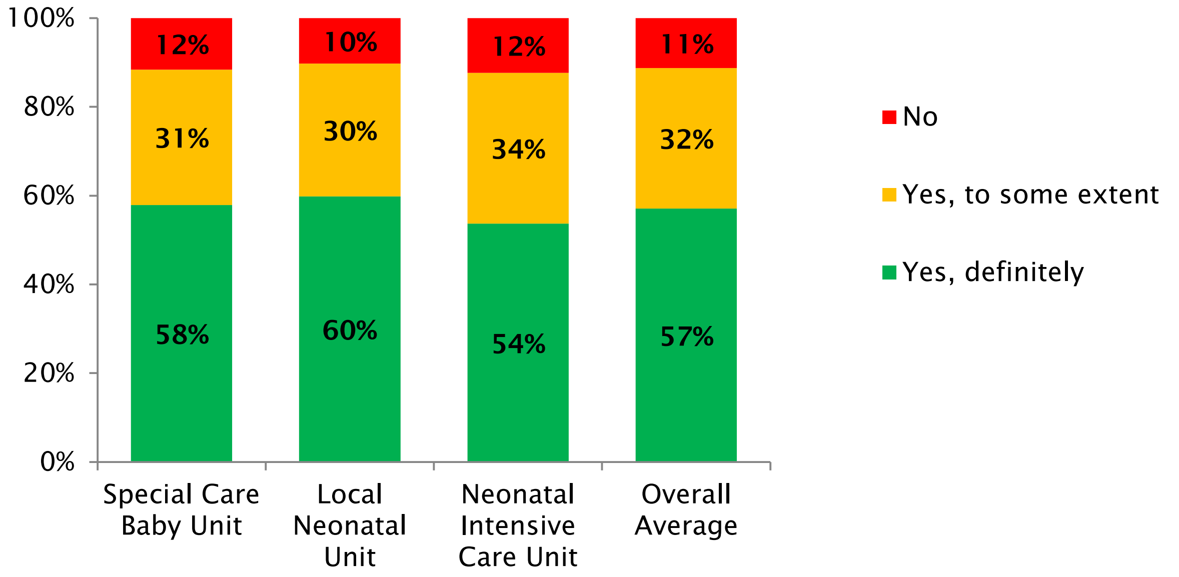

C4. Were infection control practices explained to you, such as hand washing and procedures for visitors? (Overall n=5820)

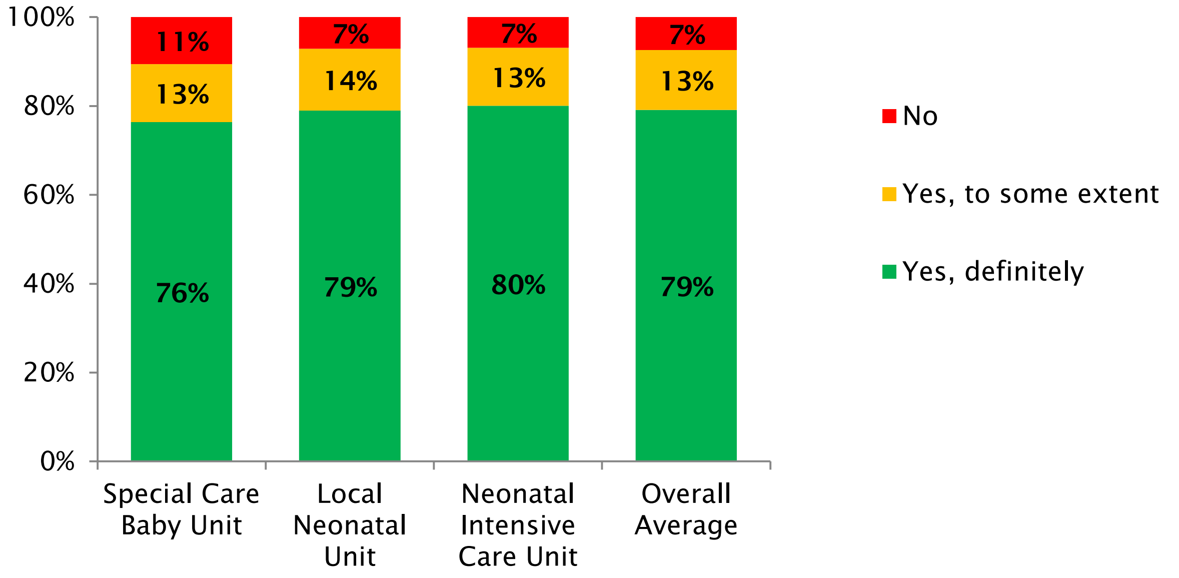

©2015 Picker Institute Europe. All Rights Reserved.

C5. Were you told which nurse was responsible for your baby's care each day s/he was in the neonatal unit? (Overall n=5888)

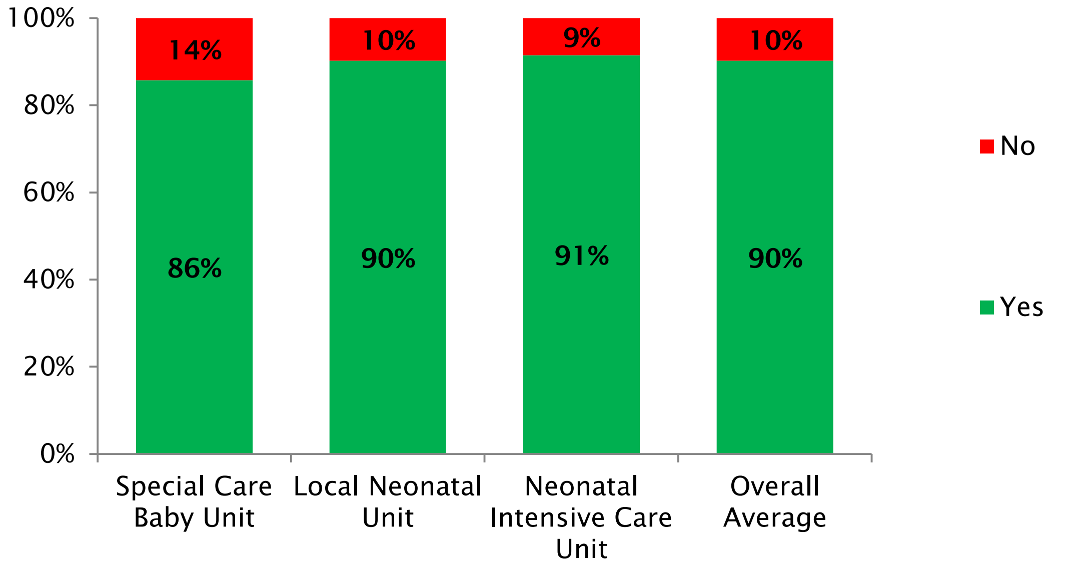

C6. Were you able to talk to staff on the unit about your worries and concerns? (Overall n=5848)

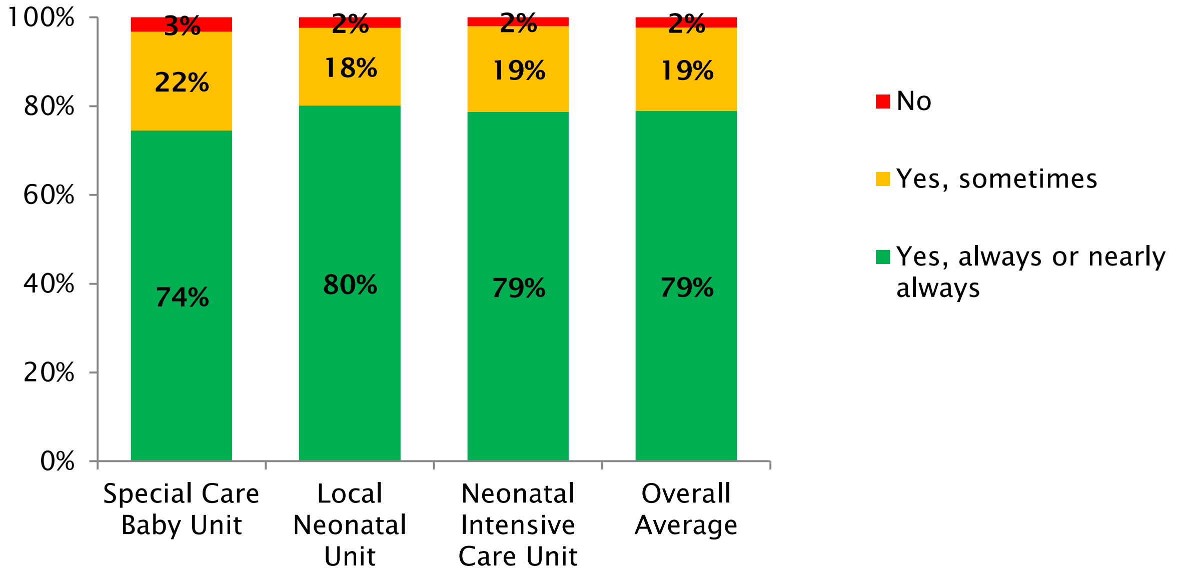

©2015 Picker Institute Europe. All Rights Reserved.

C7. Were you able to speak to a doctor about your baby as much as you wanted? (Overall n=5687)

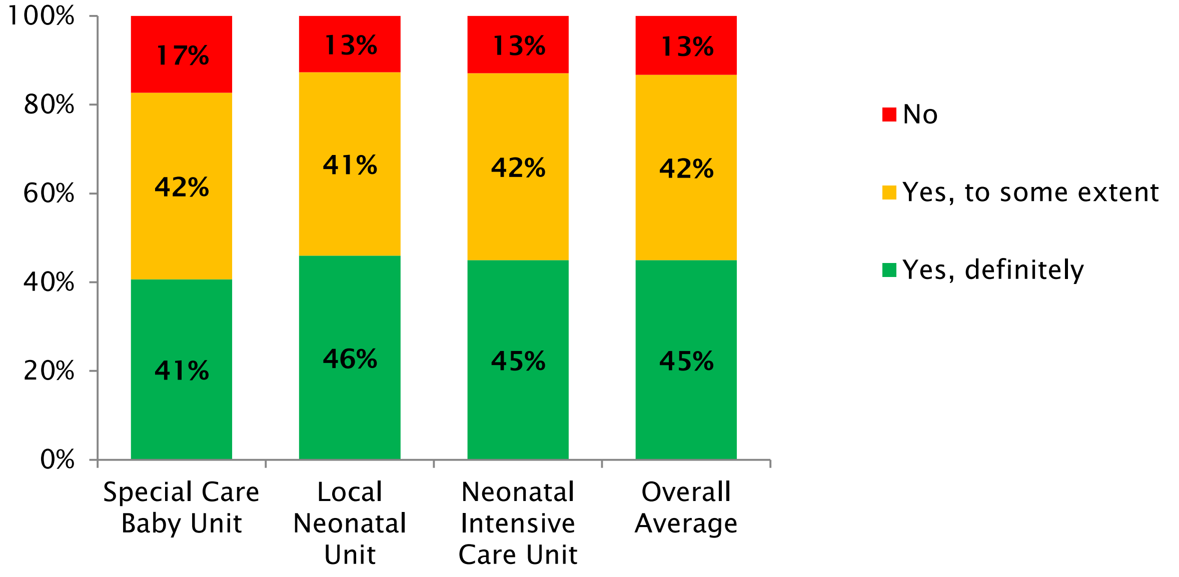

C8. Were the nurses on the unit sensitive to your emotions and feelings? (Overall n=5786)

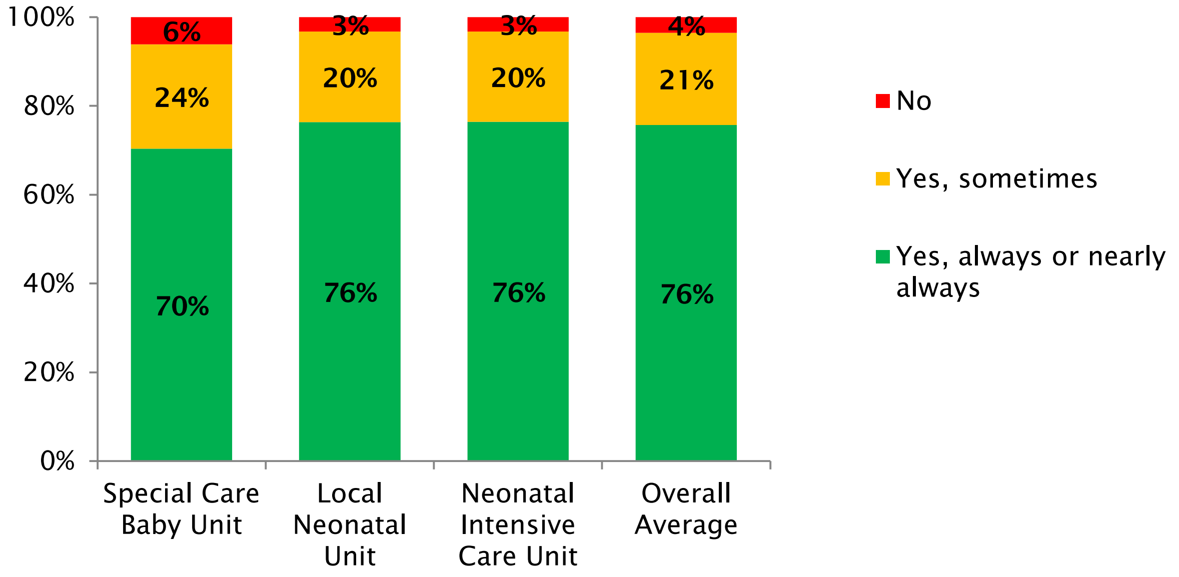

©2015 Picker Institute Europe. All Rights Reserved.

C9. Were the doctors on the unit sensitive to your emotions and feelings? (Overall n=5365)

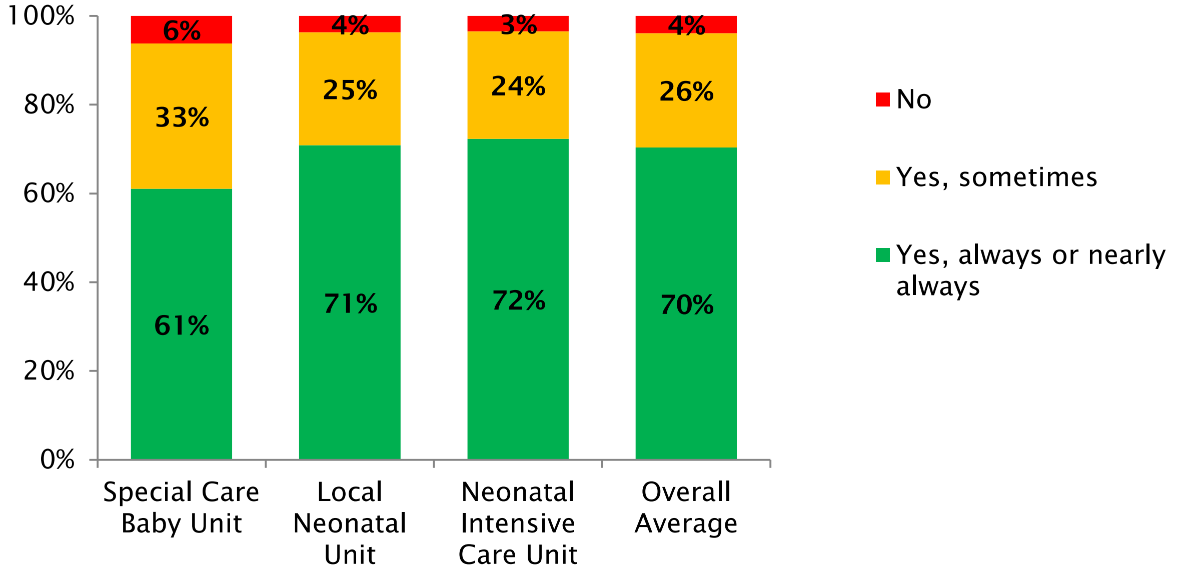

C10. In your opinion, was important information about your baby passed on from one member of staff to another? (Overall n=5603)

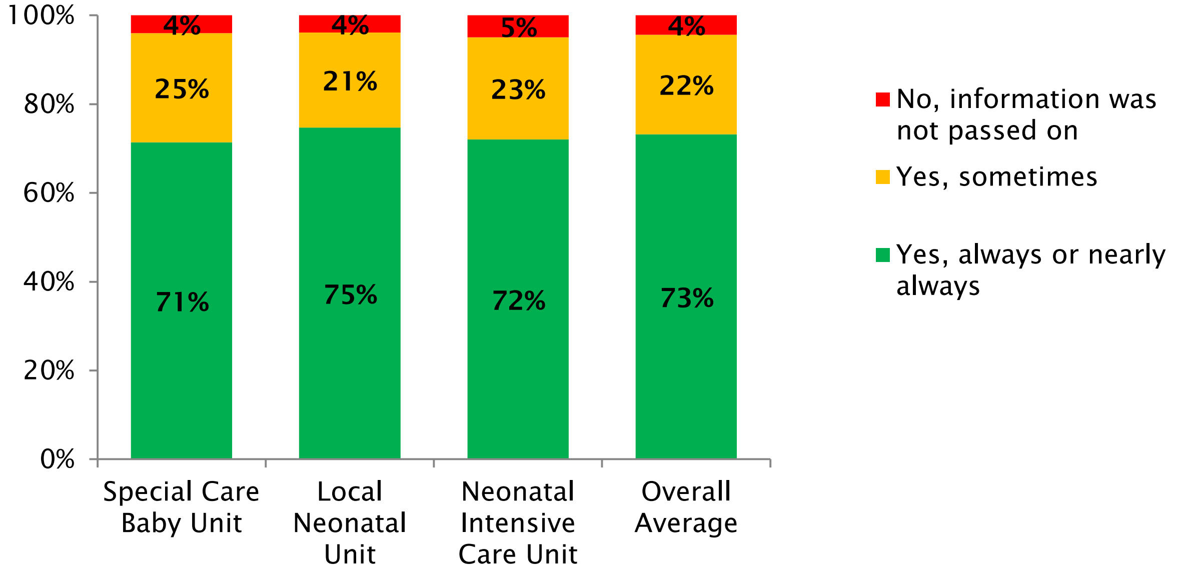

©2015 Picker Institute Europe. All Rights Reserved.

C11. Did staff give you conflicting information about your baby's condition or care? (Overall n=5868)

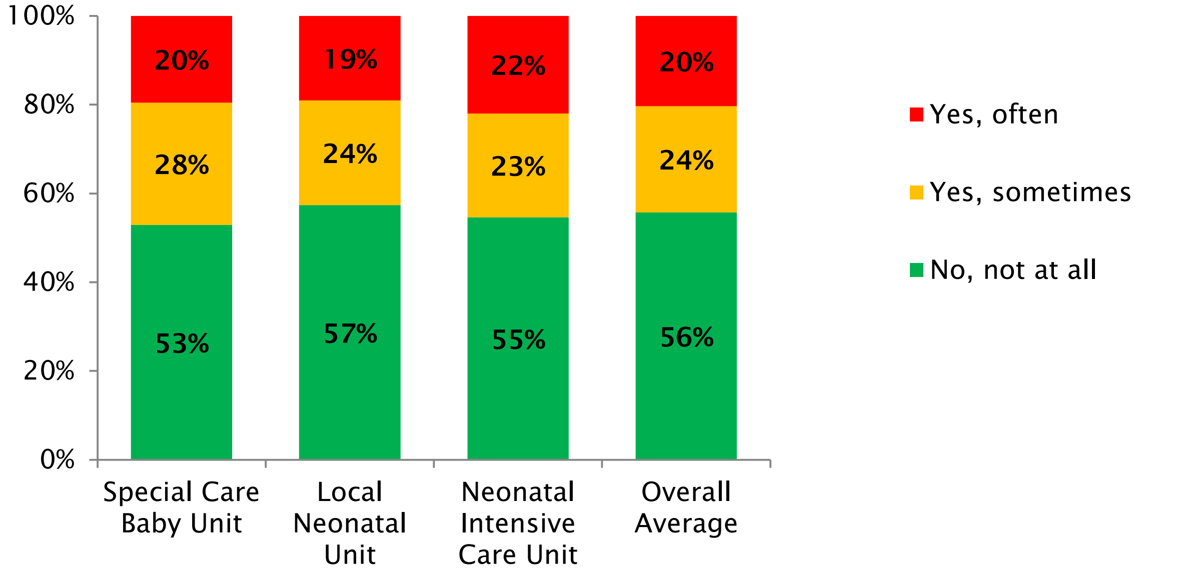

C12. Did staff refer to your baby by his/her first name? (Overall n=5459)

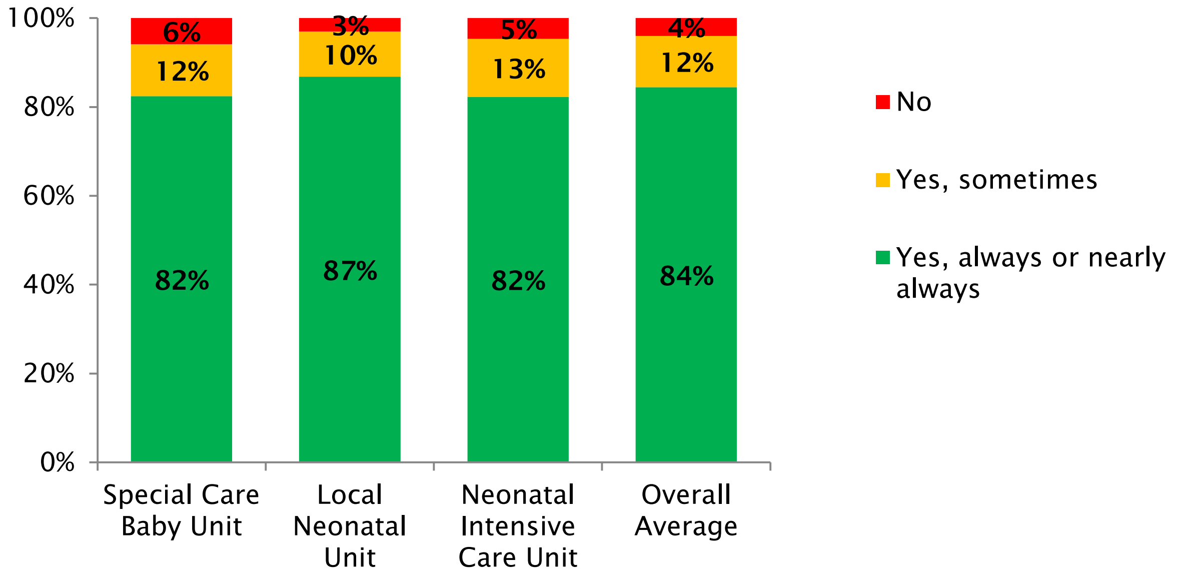

C13. Overall, did you have confidence and trust in the staff caring for your baby? (Overall n=5919)

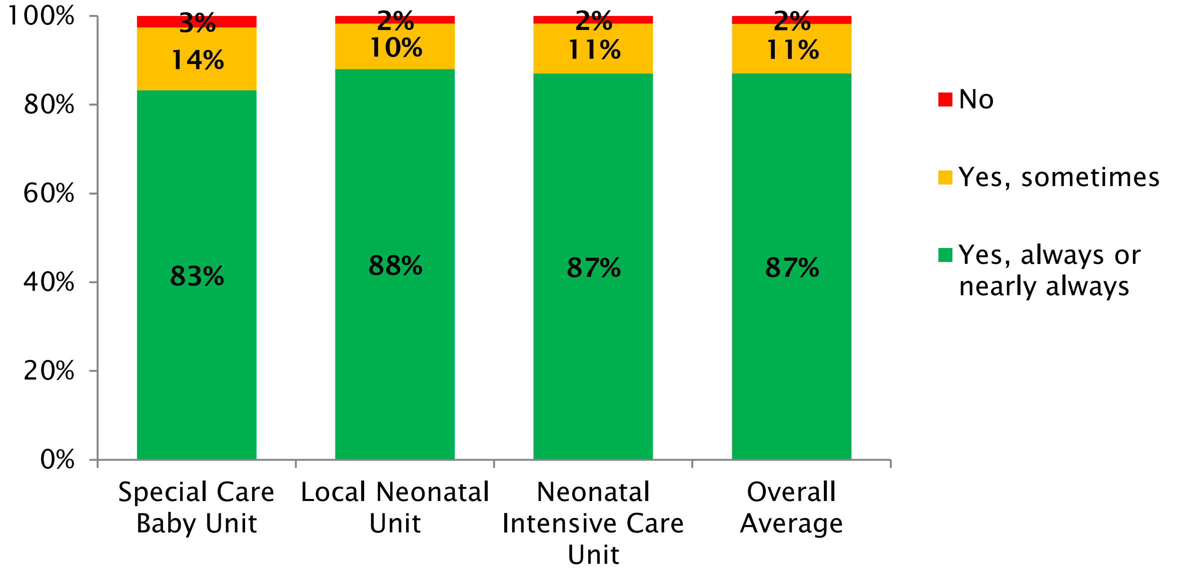

D. Your involvement in your baby's care

D1. Were you involved as much as you wanted in the day-to-day care of your baby, such as nappy changing and feeding? (Overall n=5852)

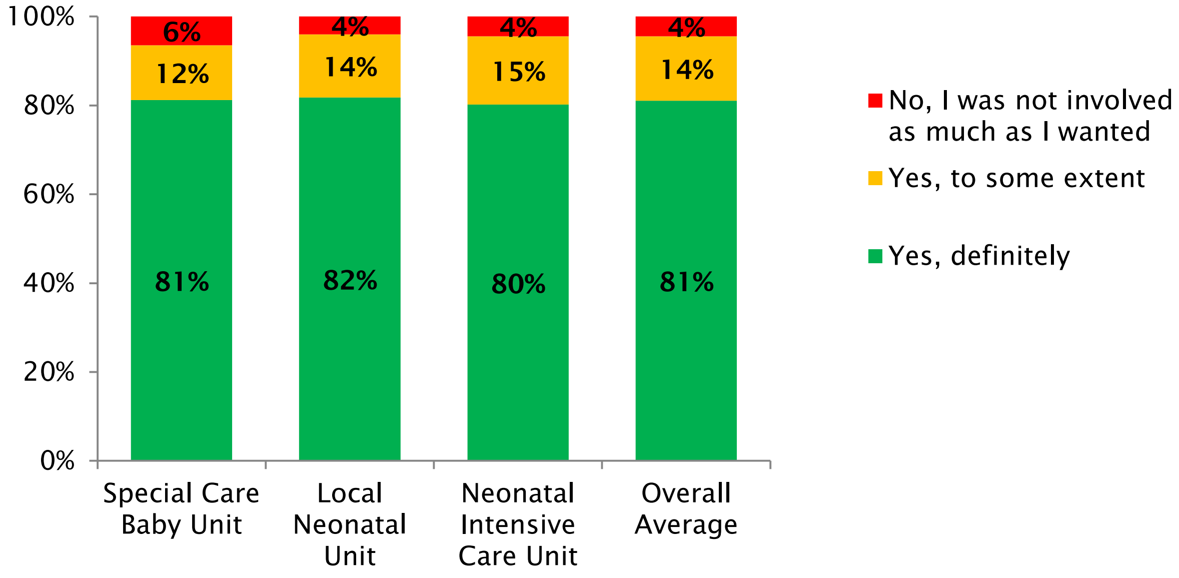

©2015 Picker Institute Europe. All Rights Reserved.

D2. Did you have as much skin- to-skin contact with your baby as you wanted? (Overall n=5106)

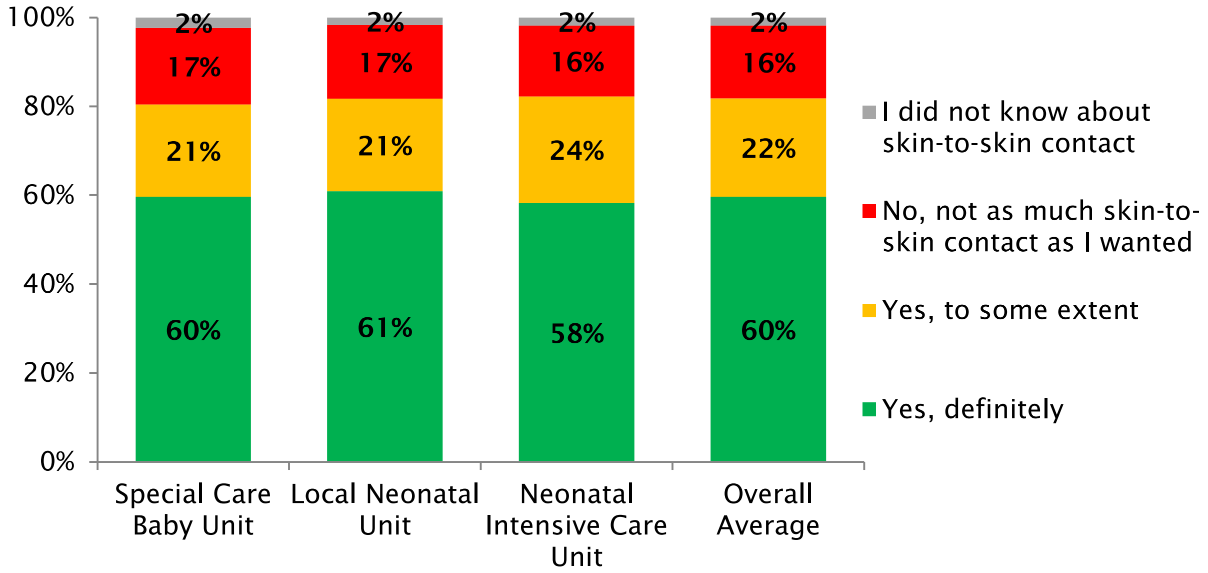

D3. Did the neonatal staff include you in discussions about your baby's care and treatment? (Overall n=5902)

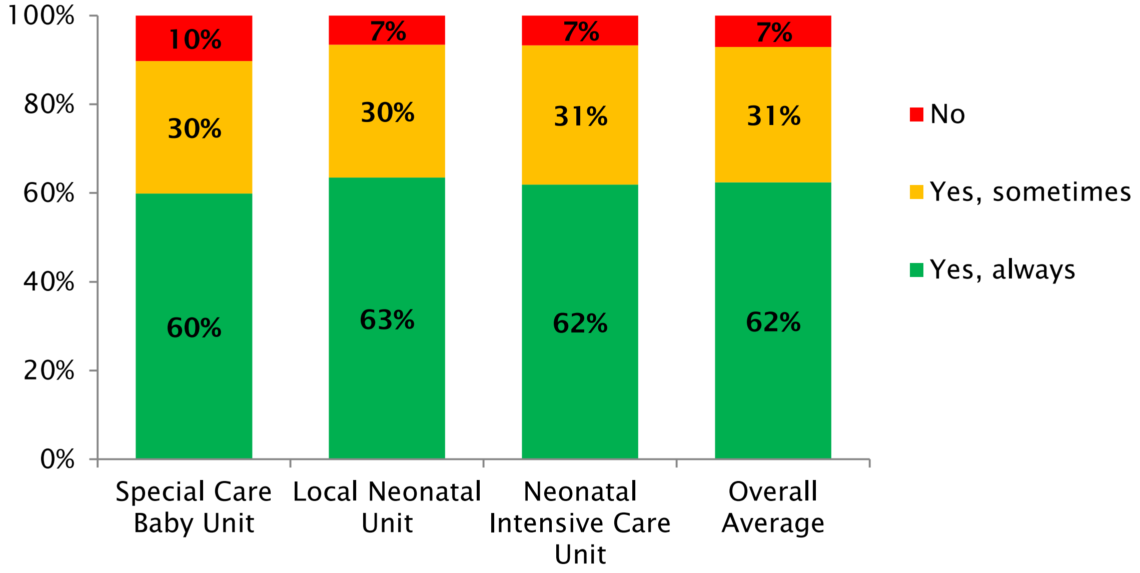

©2015 Picker Institute Europe. All Rights Reserved.

D4. Were you told about any changes in your baby's condition or care? (Overall n=5833)

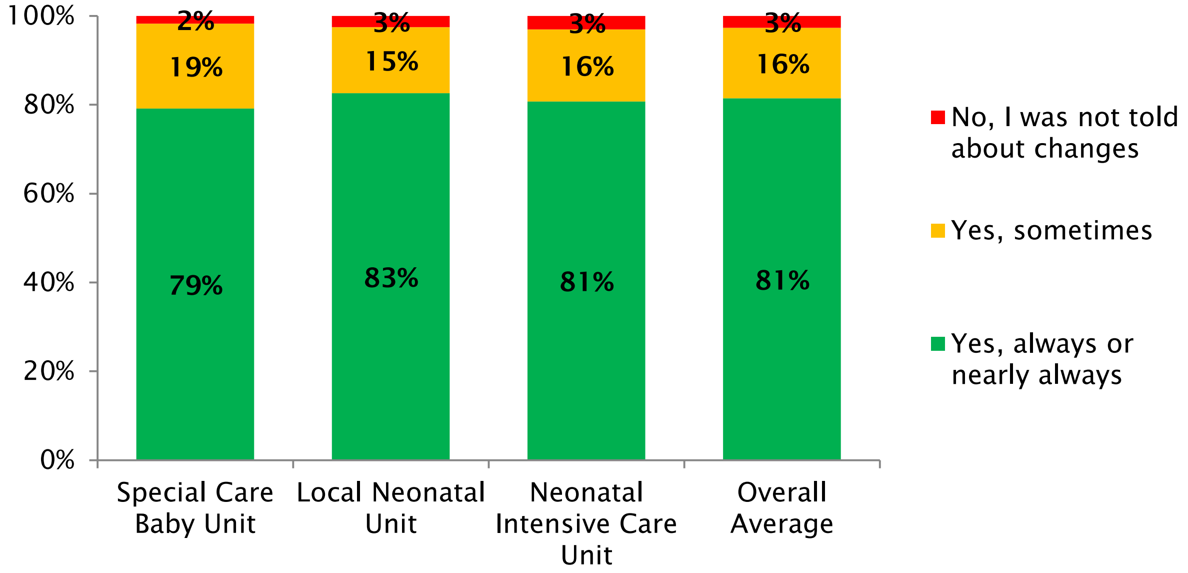

D5. When a ward round was taking place, were you allowed to be present when your baby was being discussed? (Overall n=5037)

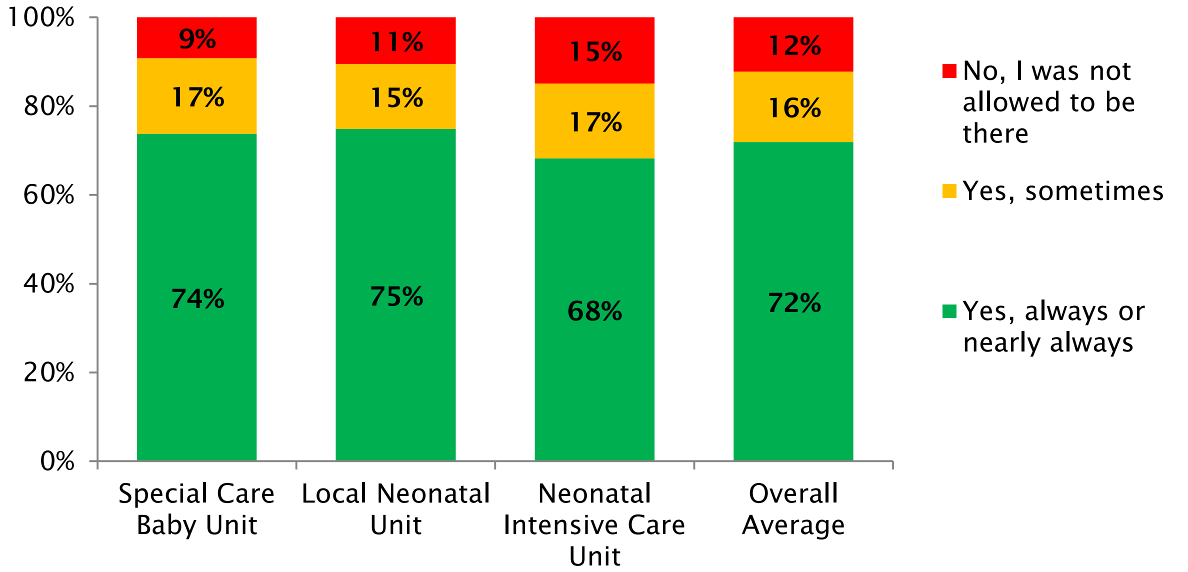

©2015 Picker Institute Europe. All Rights Reserved.

D6. Where possible, did staff arrange your baby's care (such as weighing, bathing) to fit in with your usual visiting times? (Overall n=4615)

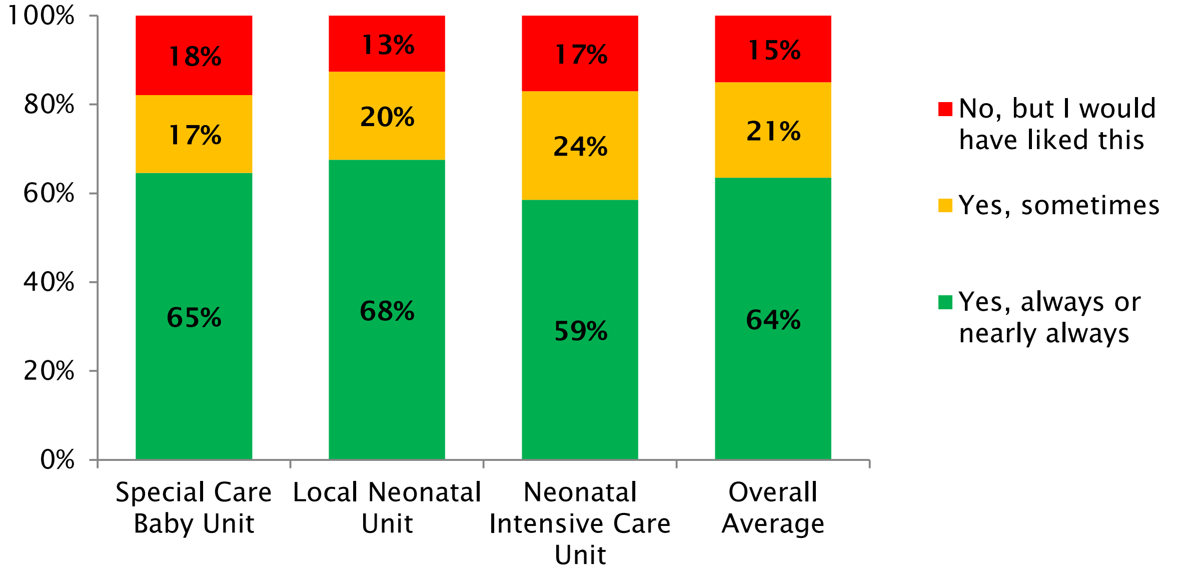

D7. Overall, did staff help you feel confident in caring for your baby? (Overall n=5876)

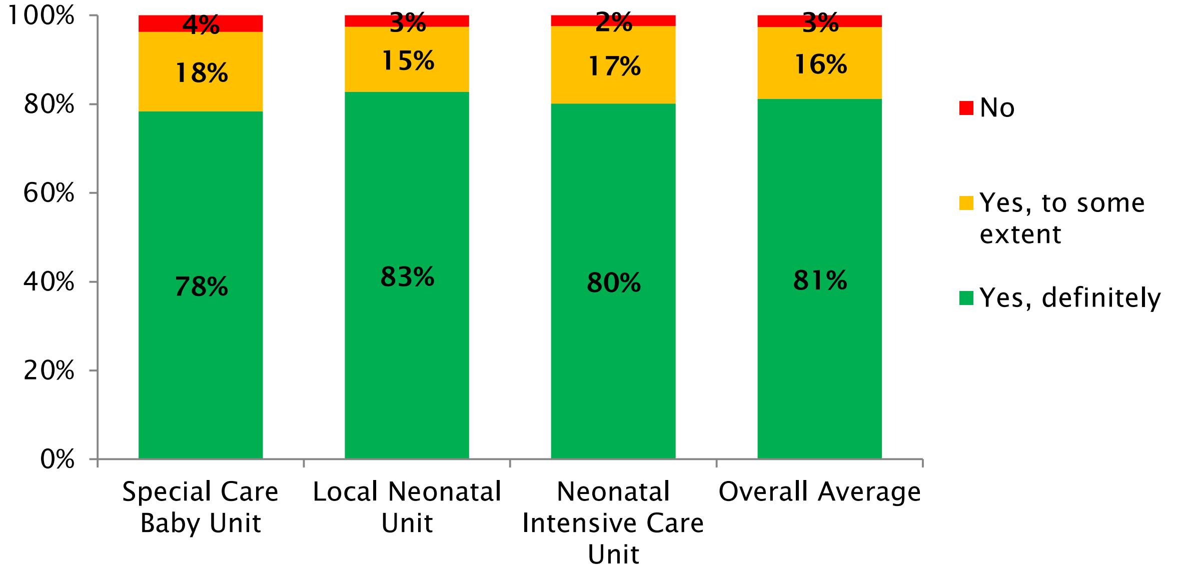

©2015 Picker Institute Europe. All Rights Reserved.

D8. If you wanted to express breast milk for your baby, were you given the support you needed from neonatal staff? (Overall n=4735)

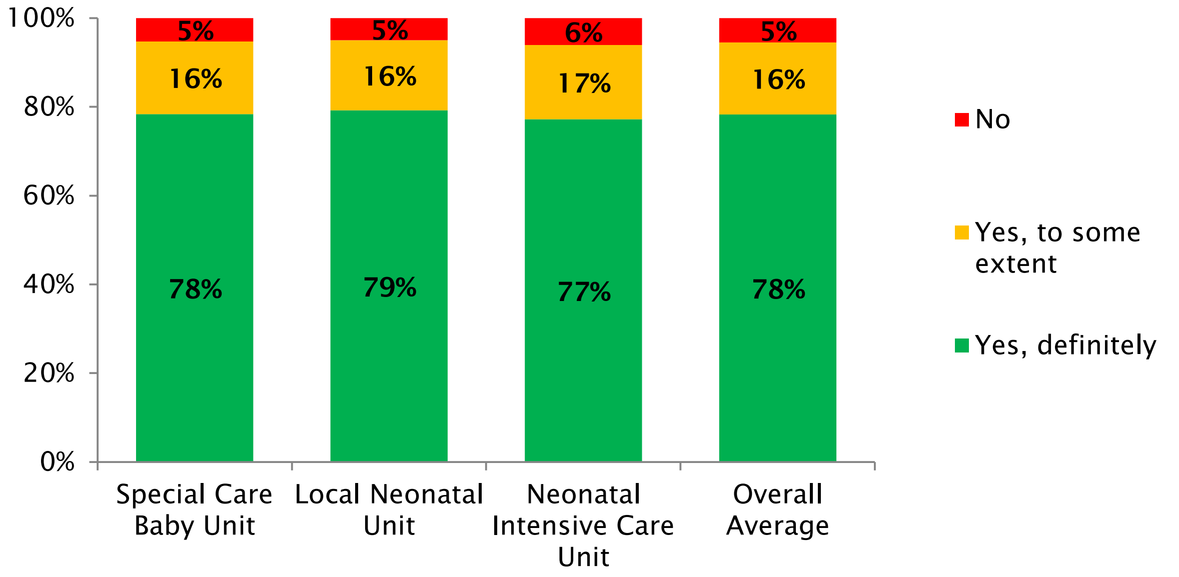

D9. When you were in the neonatal unit, were you given the feeding equipment you needed for expressing, such as a breast pump and sterilisation equipment? (Overall n=4721)

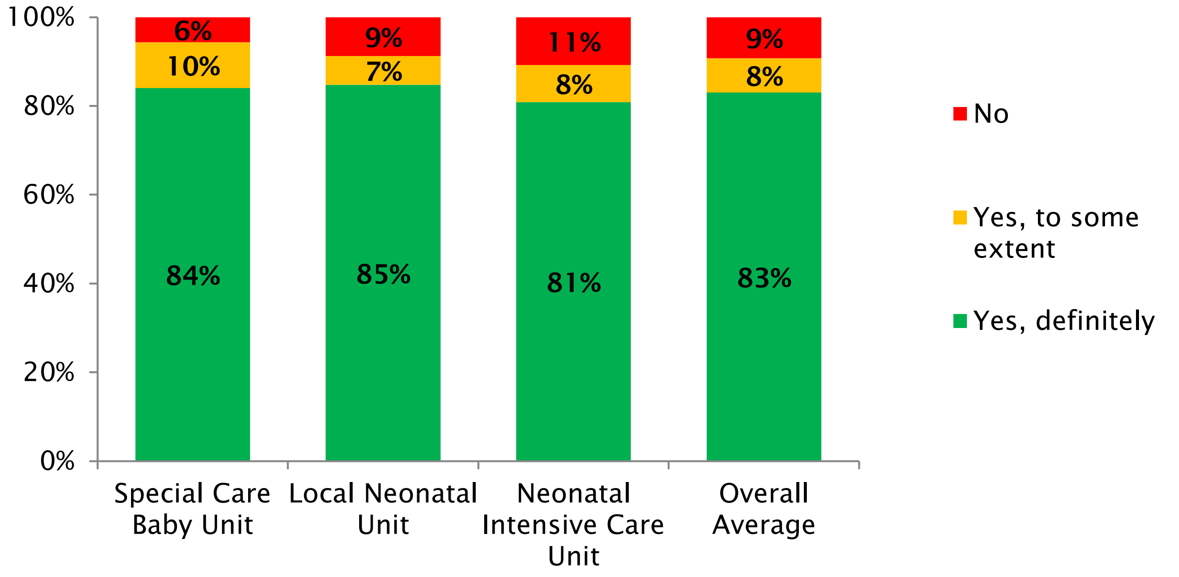

©2015 Picker Institute Europe. All Rights Reserved.

D10. Were you given enough privacy in the neonatal unit for expressing milk and/or breastfeeding your baby? (Overall n=4682)

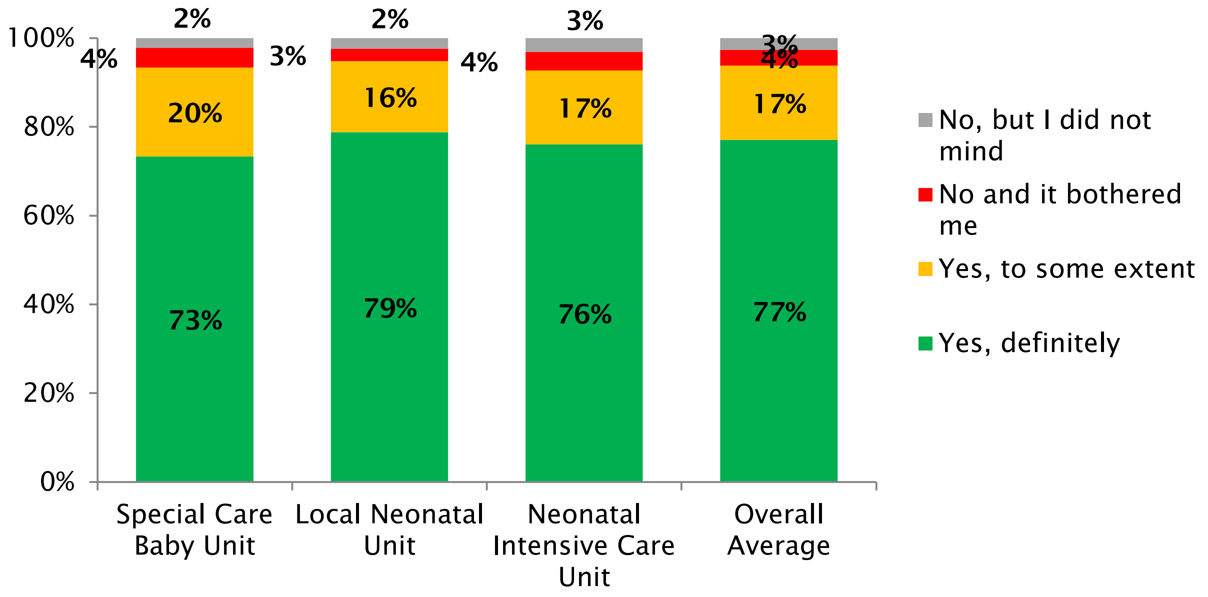

D11. If you wanted to breastfeed your baby, were you given enough support to do this from neonatal staff? (Overall n=4602)

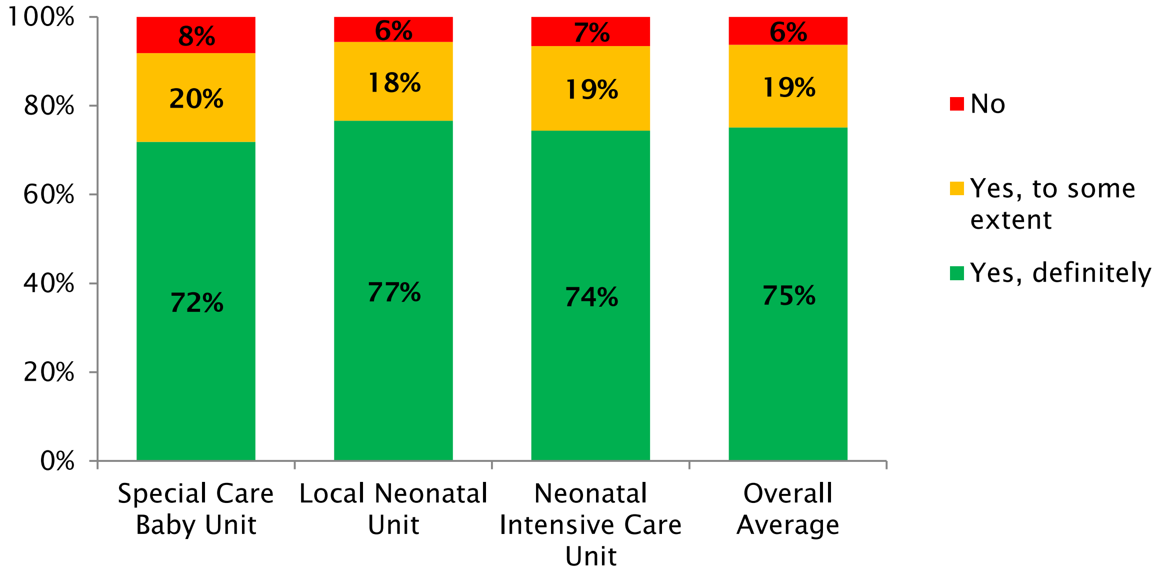

©2015 Picker Institute Europe. All Rights Reserved.

D12. If you fed your baby formula milk, were you given enough support to do this from neonatal staff? (Overall n=3507)

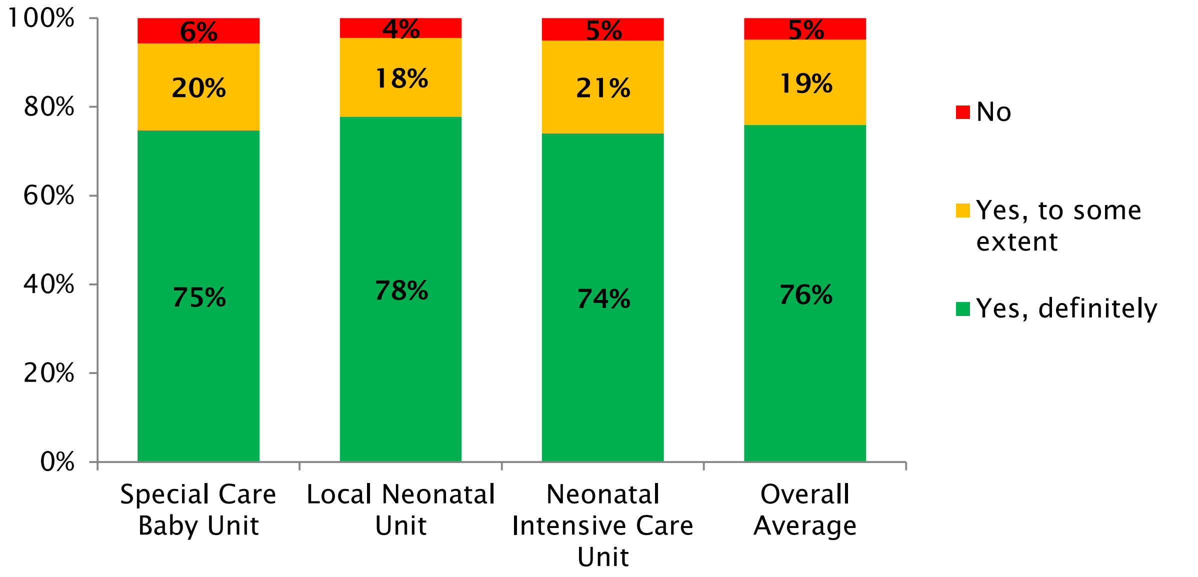

E. Environment and facilities

E1. Were you given enough privacy when discussing your baby's care on the neonatal unit with staff? (Overall n=5502)

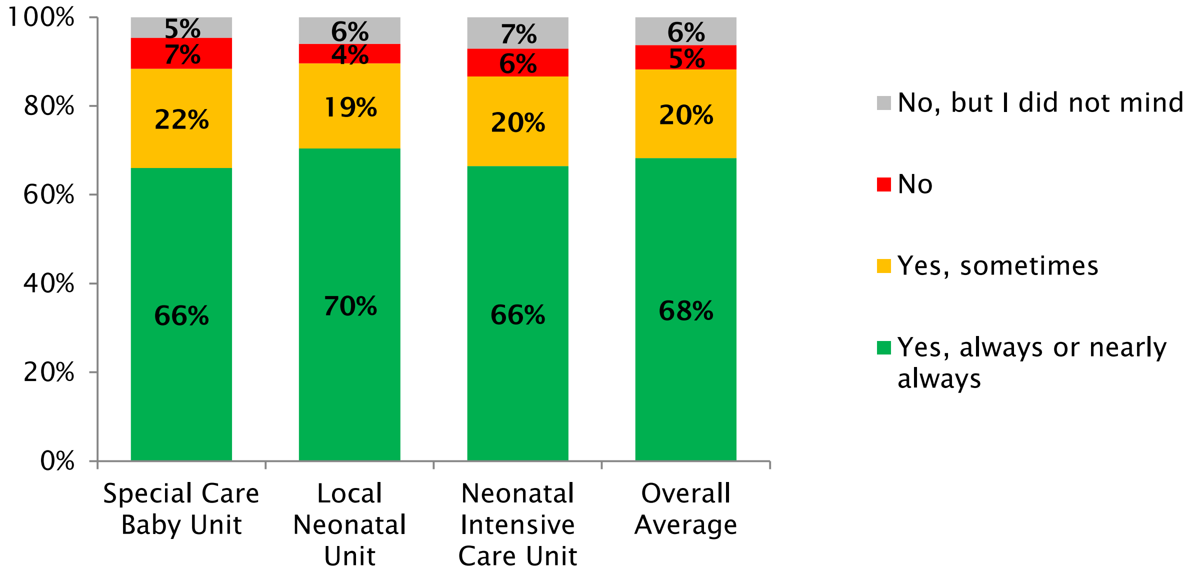

©2015 Picker Institute Europe. All Rights Reserved.

E2. Was there enough space for you to sit alongside your baby's cot in the unit? (Overall n=5875)

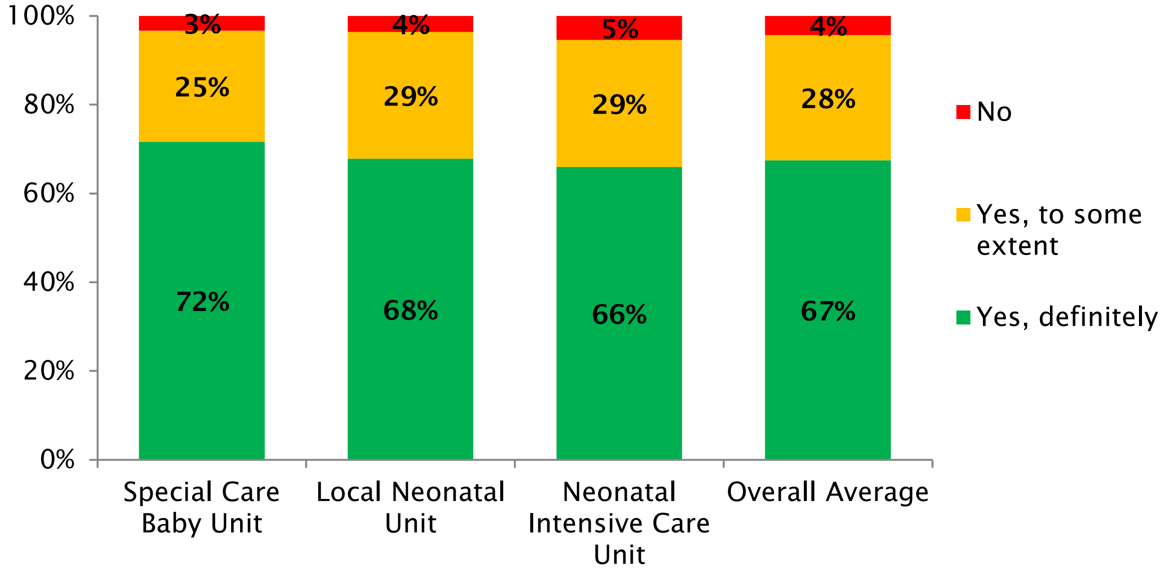

E3. In your opinion, was there adequate security on the neonatal unit? (Overall n=5894)

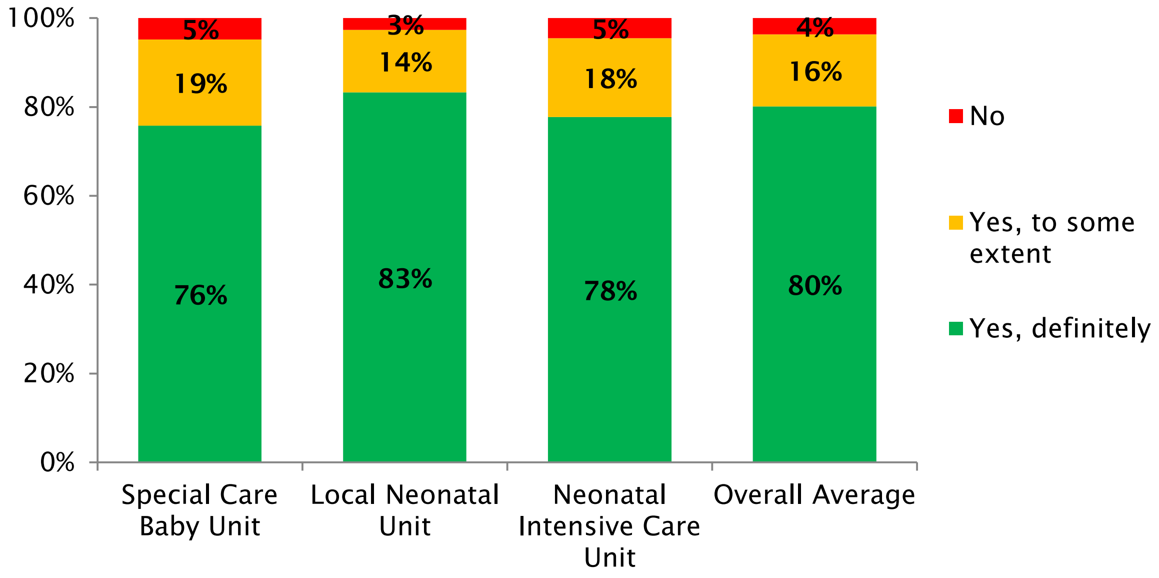

©2015 Picker Institute Europe. All Rights Reserved.

E4. If you wanted to stay overnight to be close to your baby, did the hospital offer you accommodation? (Overall n=4538)

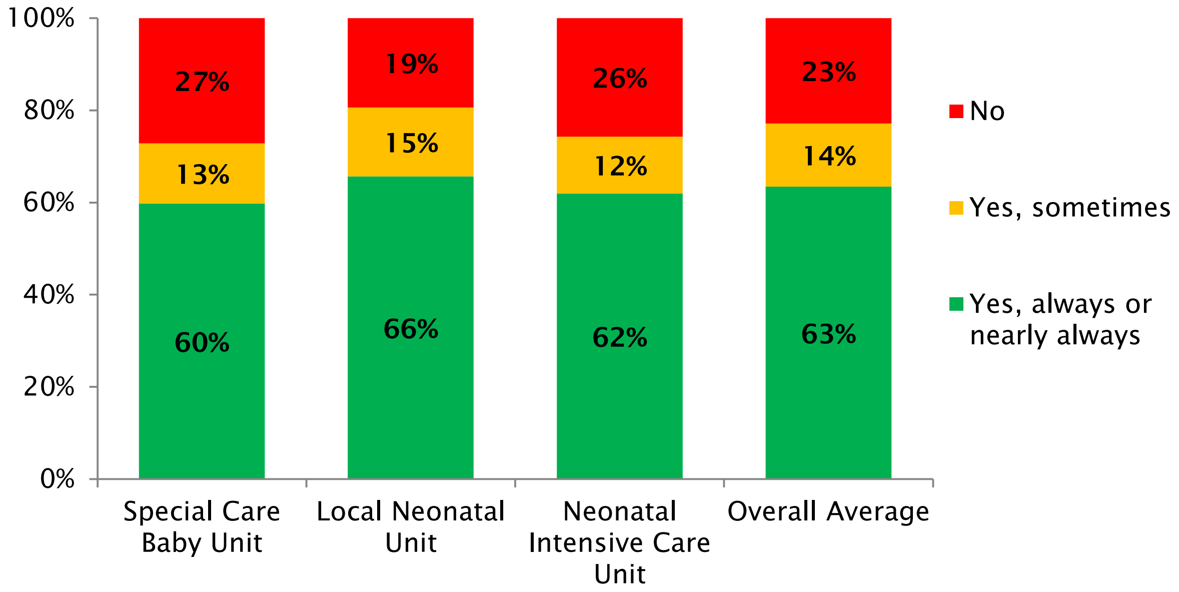

E5. Were you able to visit your baby on the unit as much as you wanted to? (Overall n=5840)

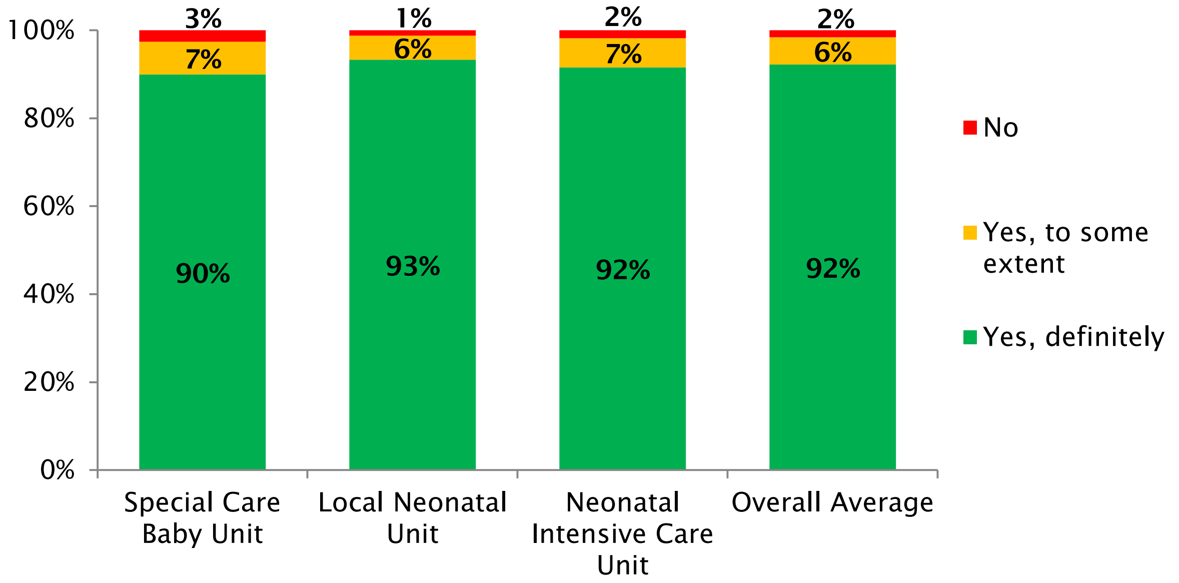

©2015 Picker Institute Europe. All Rights Reserved.

F. Information and support for parents

F1. If you asked questions about your baby's condition and treatment, did you get answers you could understand? (Overall n=5898)

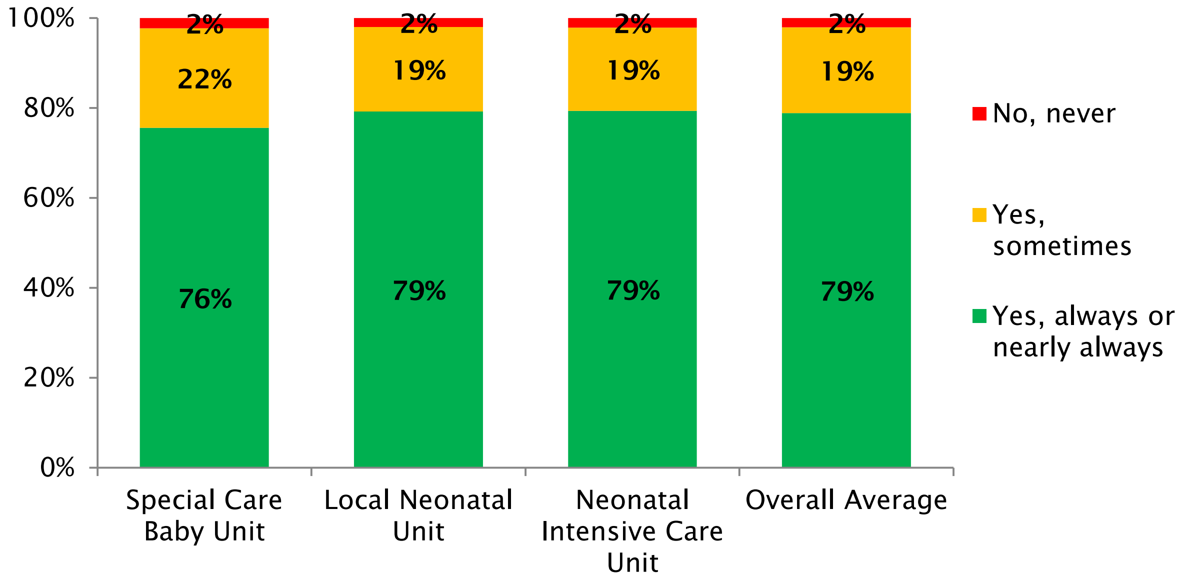

F2. Were you given enough written information to help you understand your baby's condition and treatment? (Overall n=5251)

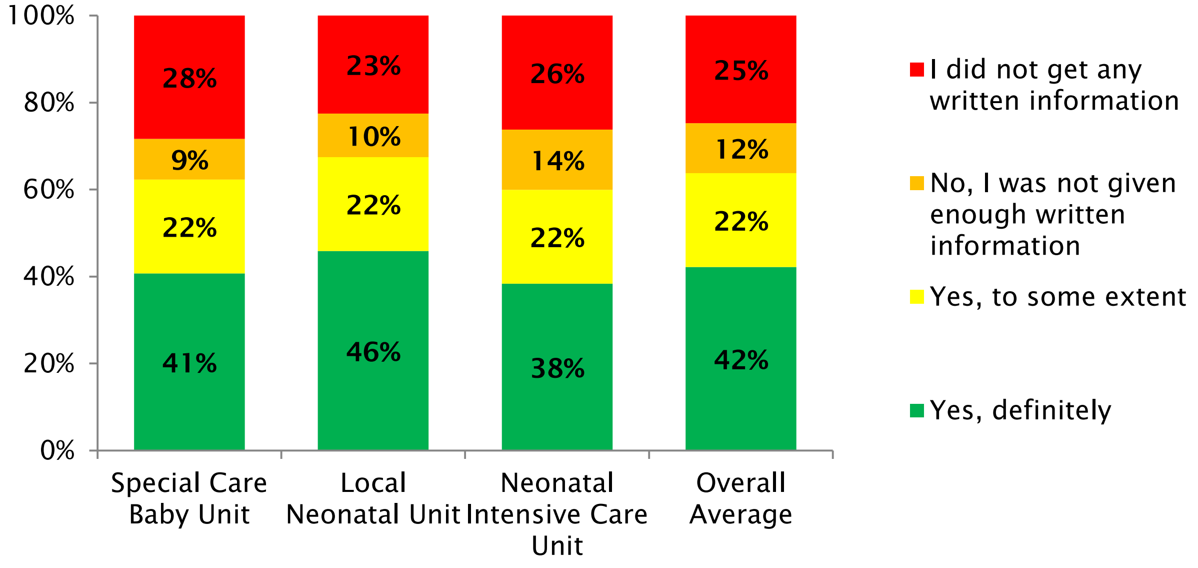

©2015 Picker Institute Europe. All Rights Reserved.

F3 Did you have an opportunity to go through your baby's medical notes with staff while they were in the neonatal unit?

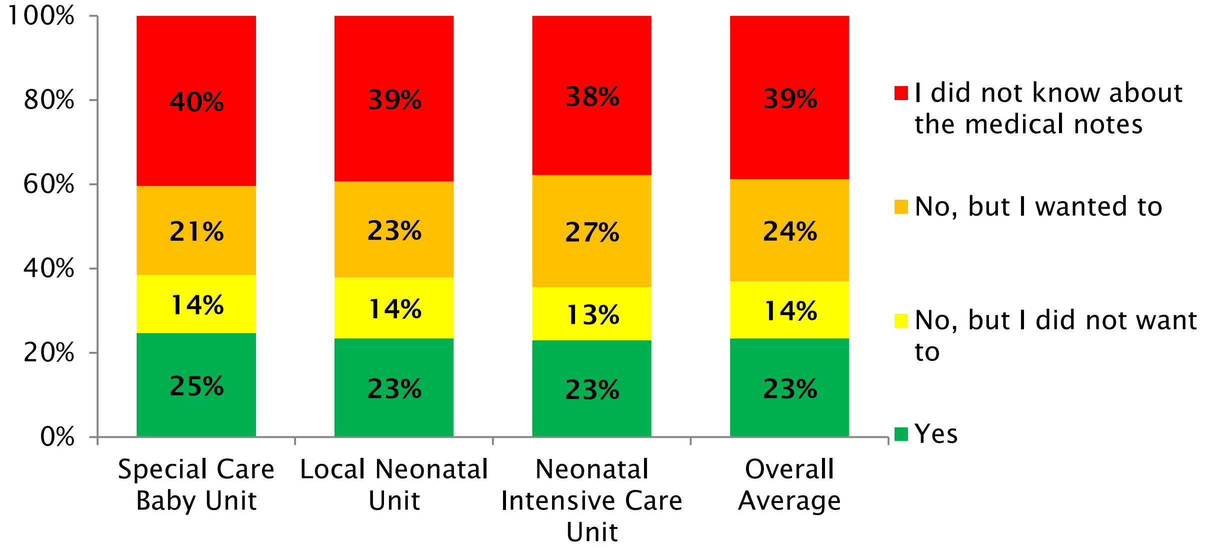

F4. Were you offered emotional support or counselling services from neonatal unit staff? (Overall n=5873)

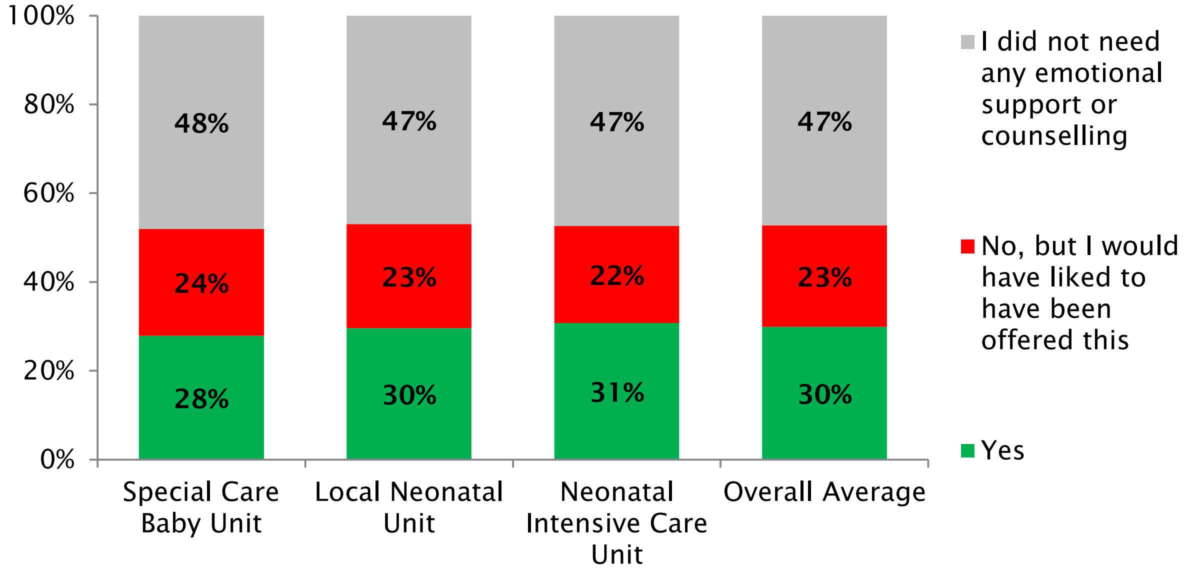

©2015 Picker Institute Europe. All Rights Reserved.

F5. Were you given enough information about help you could get with expenses related to your baby's stay in the neonatal unit? (Overall n=4709)

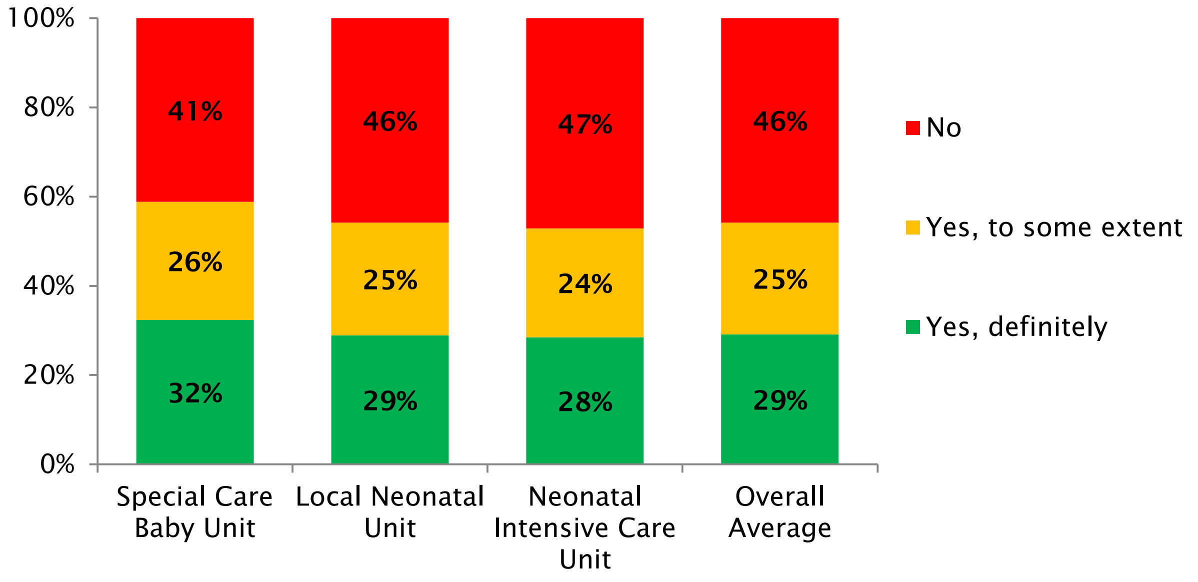

F6. Did staff give you any information about parent support groups, such as Bliss or other local groups? (Overall n=5153)

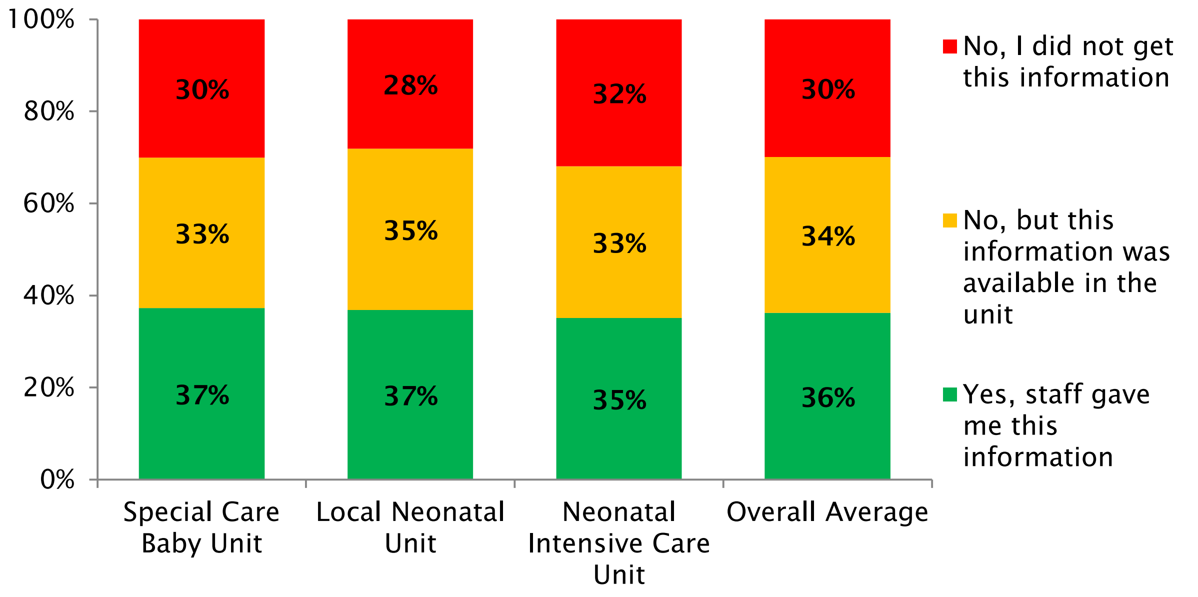

©2015 Picker Institute Europe. All Rights Reserved.

G. Leaving the neonatal unit

G3. Were you offered overnight accommodation with your baby at the hospital before they left the neonatal unit? (Overall n=3629)

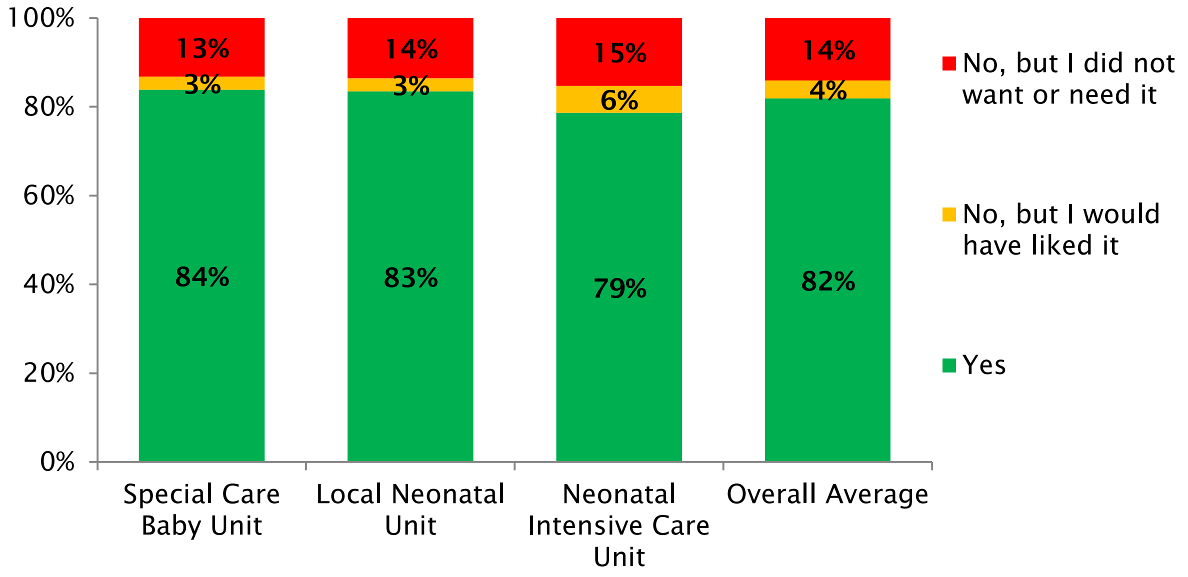

G4. Did you feel prepared for your baby's discharge from neonatal care? (Overall n=5903)

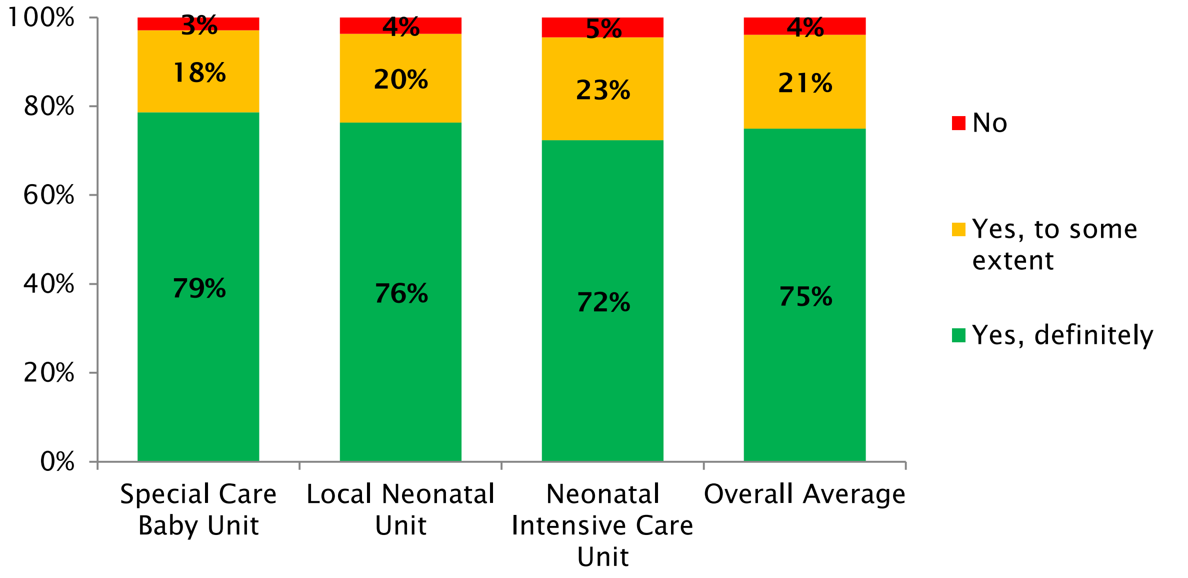

©2015 Picker Institute Europe. All Rights Reserved.

G5. Were you given enough information on what to expect in terms of your baby's progress and recovery? (Overall n=5903)

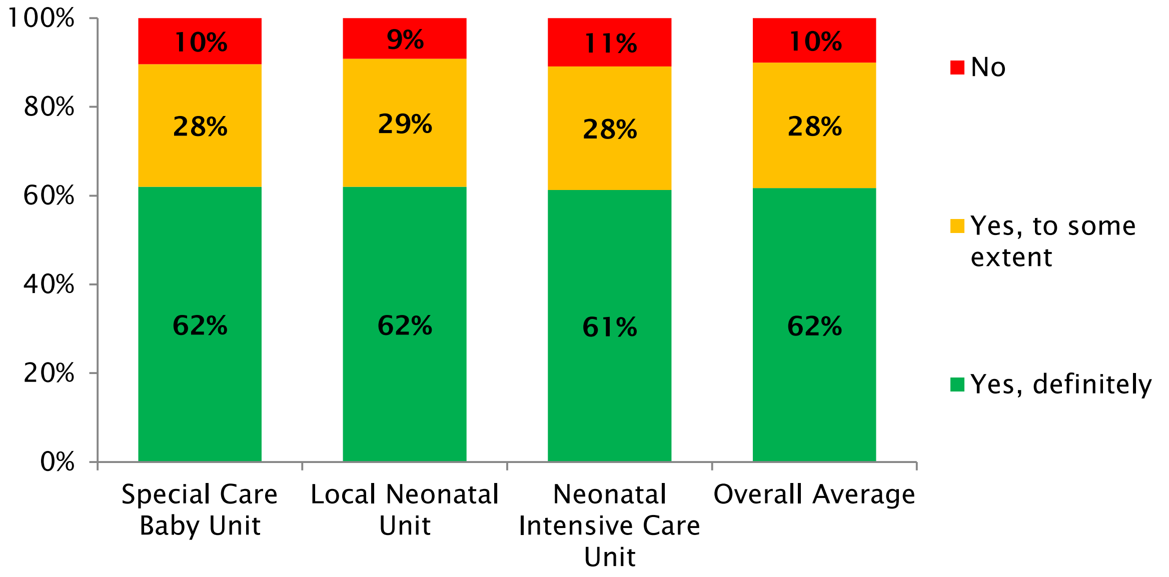

G6. How likely are you to recommend this neonatal unit to friends and family, if their baby needed similar care or treatment? (Overall n=5851)

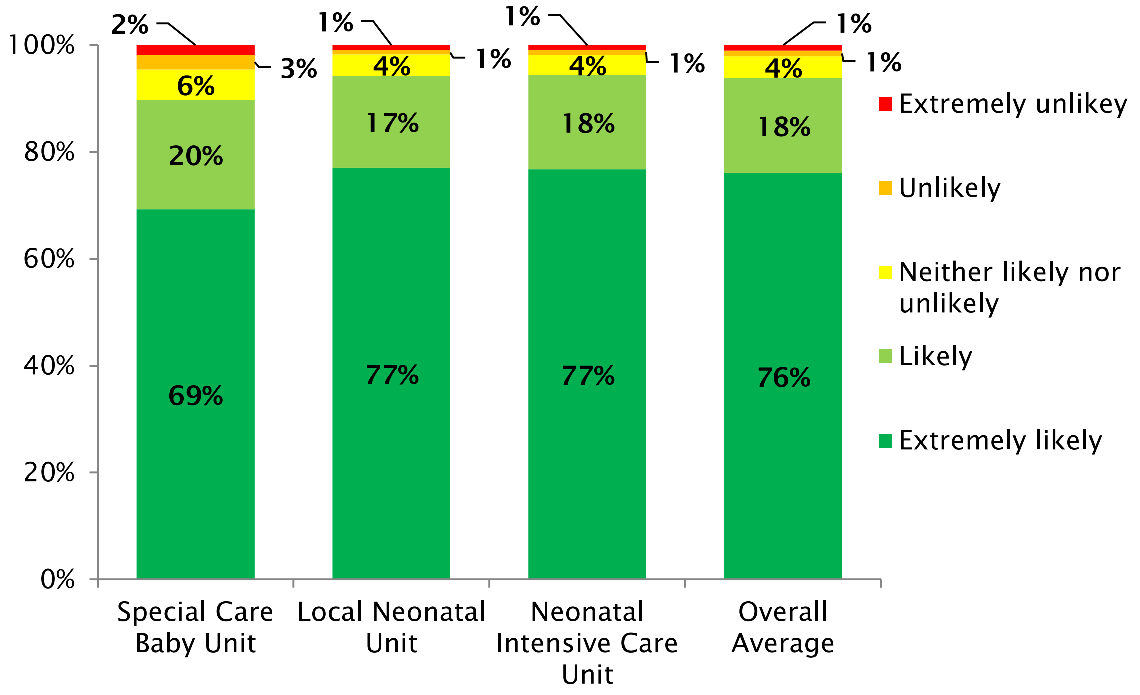

©2015 Picker Institute Europe. All Rights Reserved.

Section Six

Free Text Comments

Themes arising from feedback comments

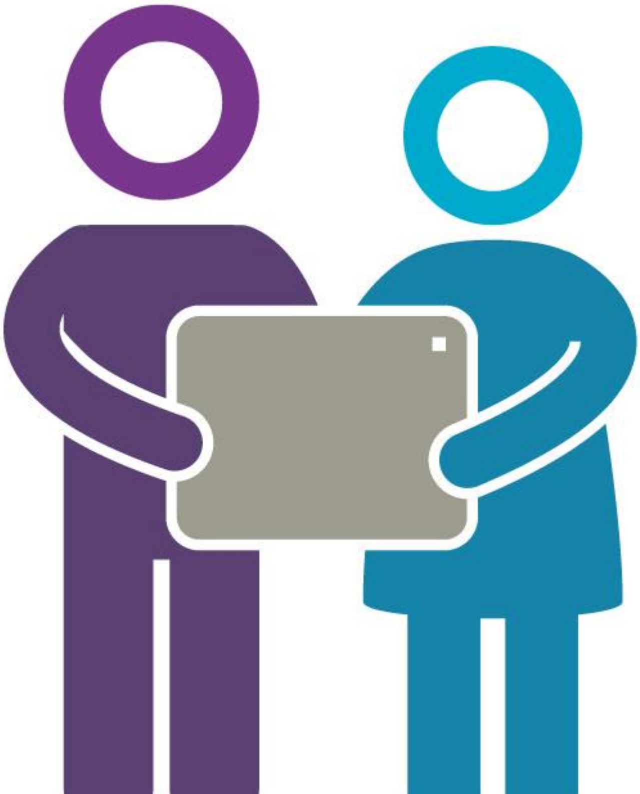

Free Text Comments

Respondents were given the option to provide open-ended feedback answering *“If there is anything else you would like to tell us about the neonatal care received, please do so here”.* Over 3700 participants provided comments. These are presented as a word cloud. Anonymised verbatim comments are available upon request from the Picker Institute.

Word Clouds

Word clouds are a visualisation of the verbatim comments supplied by those who responded to the free text question. It performs a count of the frequency of words to produce an overall imagine. The more a word (or phrase) is mentioned, the bigger the size of the work in the image. All the words are adjusted proportionately and randomly placed with the cloud.

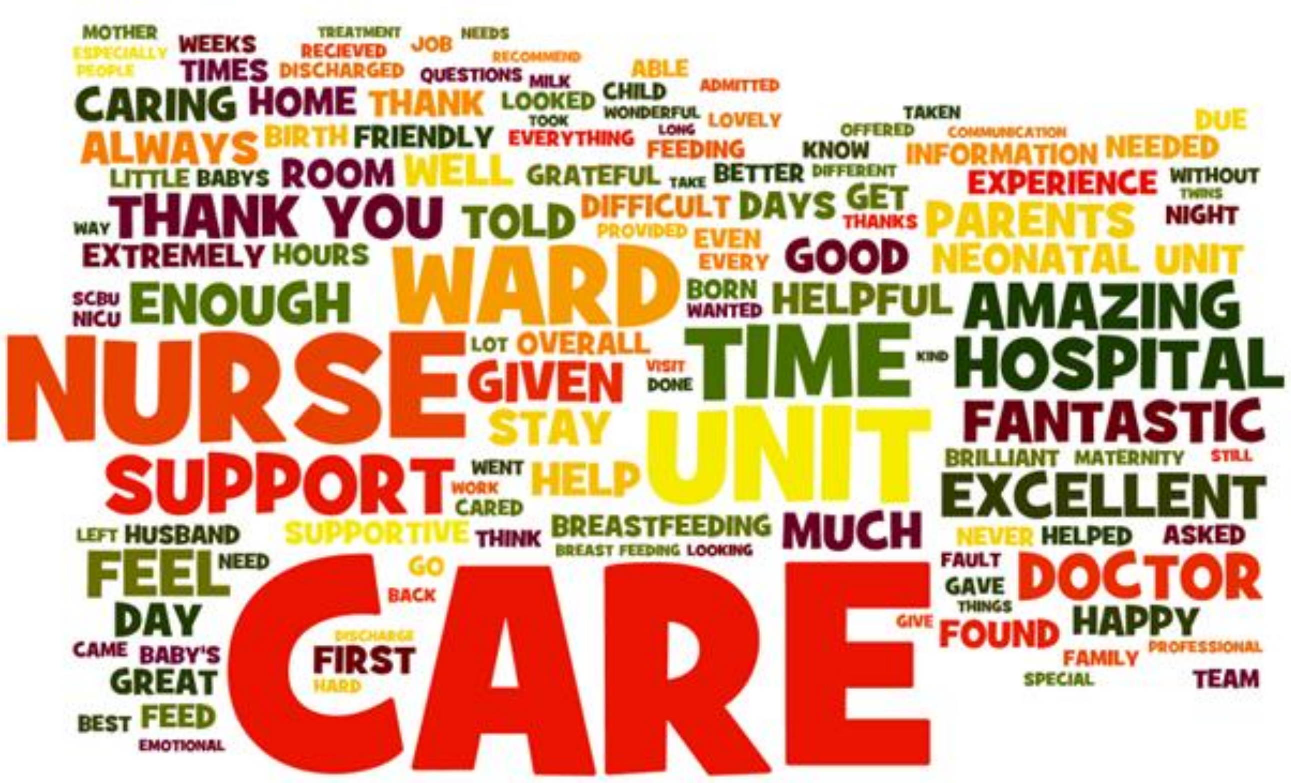

©2015 Picker Institute Europe. All Rights Reserved.

Appendix A

Frequency Tables

Detailed breakdown of overall results by question

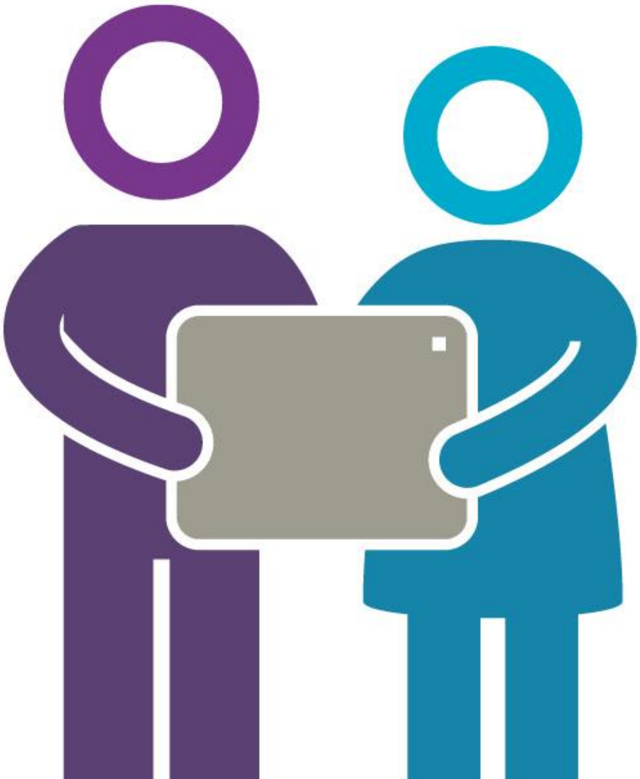

Frequency tables

This section provides a breakdown of overall responses from all units for each question. It also shows which groups of patients responded to each question and which response options are included in the total specific responses included in percentage calculations.

A. Before your baby was born

A1 Before your baby was born (i.e. during pregnancy or labour), did you know that they might need care in a neonatal unit?	Number	Percentage
Yes	2031	34%
No	3880	66%
Total specific responses	5911	100%
Missing responses	43	0%

Answered by all

A2 Before your baby was born (ie during pregnancy or labour), did a member of staff from the neonatal unit talk to you about what to expect after the birth?	Number	Percentage
Yes, definitely	845	44%
Yes, to some extent	405	22%
No	669	34%
Total specific responses	1919	100%
Don't know / can't remember	104	0%
Missing responses	24	0%

Answered by those who knew before their baby was born that it might need care in a neonatal unit

©2015 Picker Institute Europe. All Rights Reserved.

B. Your baby's admission to neonatal care

B1 Was your baby first admitted to the neonatal unit named in the letter sent with this questionnaire?

	Number	Percentage
Yes	5339	92%
No, my baby was first admitted to a neonatal unit at a different hospital	476	8%
Total specific responses	5815	100%
Missing responses	139	0%

Answered by all

B2 After you gave birth, were you offered a photograph of your baby?

	Number	Percentage
Yes	2276	46%
No, but I would have liked this	1225	25%
No, but I took a photograph myself	1471	29%
Total specific responses	4972	100%
I did not want a photograph taken	104	0%
Don't know/can't remember	229	0%
Missing responses	132	0%

Answered by those whose baby was first admitted to named unit

B3 After you gave birth, were you ever cared for in the same ward as mothers who had their baby with them?

	Number	Percentage
Yes, and this bothered me	1590	31%
Yes, but I did not mind	1423	28%
No, I stayed in a separate room/area	2154	42%
Total specific responses	5167	100%
I was discharged from hospital	145	0%
Missing responses	127	0%

Answered by those whose baby was first admitted to named unit

B4 After your baby was admitted to the neonatal unit, were you able to see your baby as soon as you wanted?

	Number	Percentage
Yes	3502	91%
No	356	9%
Total specific responses	3858	100%
No, but this was not possible for medical reasons (baby and/or mother)	1495	0%
No, because we were in different hospitals	24	0%
Missing responses	71	0%

Answered by those whose baby was first admitted to named unit

©2015 Picker Institute Europe. All Rights Reserved.

C. Staff on the neonatal unit

C1 When you visited the unit, did the staff caring for your baby introduce themselves to you?	Number	Percentage
All of the staff introduced themselves	3657	63%
Some of the staff introduced themselves	1911	33%
Very few or none of the staff introduced themselves	236	4%
Total specific responses	5804	100%
Don't know / can't remember	95	0%
Missing responses	55	0%

Answered by all

C2 Were you given enough information about the neonatal unit (such as rules, procedures and facilities for parents)	Number	Percentage
Yes, definitely	3618	62%
Yes, to some extent	1661	29%
No	525	9%
Total specific responses	5804	100%
Can't remember	105	0%
Missing responses	45	0%

Answered by all

C3 Was the purpose of the machines, monitors and alarms used in the neonatal unit clearly explained to you?	Number	Percentage
Yes, definitely	3306	57%
Yes, to some extent	1816	32%
No	645	11%
Total specific responses	5767	100%
Don't know / can't remember	114	0%
Missing responses	73	0%

Answered by all

C4 Were infection control practices explained to you, such as hand washing and procedures for visitors?	Number	Percentage
Yes, definitely	4602	79%
Yes, to some extent	783	13%
No	435	7%
Total specific responses	5820	100%
Don't know / can't remember	88	0%
Missing responses	46	0%

Answered by all

©2015 Picker Institute Europe. All Rights Reserved.

C5 Were you told which nurse was responsible for your baby's care each day s/he was in the neonatal unit?

	Number	Percentage
Yes	5323	90%
No	565	10%
Total specific responses	5888	100%
Missing responses	66	0%

Answered by all

C6 Were you able to talk to staff on the unit about your worries and concerns?

	Number	Percentage
Yes, always or nearly always	4606	79%
Yes, sometimes	1116	19%
No	126	2%
Total specific responses	5848	100%
I had no worries or concerns	60	0%
Missing responses	46	0%

Answered by all

C7 Were you able to speak to a doctor about your baby as much as you wanted?

	Number	Percentage
Yes, definitely	2543	45%
Yes, to some extent	2382	42%
No	762	13%
Total specific responses	5687	100%
I did not want or need to speak to a doctor	218	0%
Missing responses	49	0%

Answered by all

C8 Were the nurses on the unit sensitive to your emotions and feelings?

	Number	Percentage
Yes, always or nearly always	4380	76%
Yes, sometimes	1196	21%
No	210	4%
Total specific responses	5786	100%
This was not necessary	110	0%
Missing responses	58	0%

Answered by all

©2015 Picker Institute Europe. All Rights Reserved.

C9 Were the doctors on the unit sensitive to your emotions and feelings?

	Number	Percentage
Yes, always or nearly always	3787	70%
Yes, sometimes	1369	26%
No	209	4%
Total specific responses	5365	100%
I had no contact with doctors on the unit	296	0%
This was not necessary	232	0%
Missing responses	61	0%

Answered by all

C10 In your opinion, was important information about your baby passed on from one member of staff to another?

	Number	Percentage
Yes, always or nearly always	4078	73%
Yes, sometimes	1269	22%
No, information was not passed on	256	4%
Total specific responses	5603	100%
Don't know / can't remember	303	0%
Missing responses	48	0%

Answered by all

C11 Did staff give you conflicting information about your baby's condition or care?

	Number	Percentage
Yes, often	1177	20%
Yes, sometimes	1391	24%
No, not at all	3300	56%
Total specific responses	5868	100%
Missing responses	86	0%

Answered by all

C12 Did staff refer to your baby by his/her first name?

	Number	Percentage
Yes, always or nearly always	4594	84%
Yes, sometimes	647	12%
No	218	4%
Total specific responses	5459	100%
My baby did not have a name	451	0%
Missing responses	44	0%

Answered by all

©2015 Picker Institute Europe. All Rights Reserved.

C13 Overall, did you have confidence and trust in the staff caring for your baby?

	Number	Percentage
Yes, always or nearly always	5140	87%
Yes, sometimes	667	11%
No	112	2%
Total specific responses	5919	100%
Missing responses	35	0%

Answered by all

©2015 Picker Institute Europe. All Rights Reserved.

D. Your involvement in your baby's care

D1 Were you involved as much as you wanted in the day-to-day care of your baby, such as nappy changing and feeding?	Number	Percentage
Yes, definitely	4768	81%
Yes, to some extent	831	14%
No, I was not involved as much as I wanted	253	4%
Total specific responses	5852	100%
No, my baby was too ill	57	0%
Missing responses	45	0%

Answered by all

D2 Did you have as much skin- to-skin contact with your baby as you wanted?	Number	Percentage
Yes, definitely	3070	60%
Yes, to some extent	1114	22%
No, not as much skin-to-skin contact as I wanted	832	16%
I did not know about skin-to-skin contact	90	2%
Total specific responses	5106	100%
No, but this was not possible for medical reasons	771	0%
Missing responses	77	0%

Answered by all

D3 Did the neonatal staff include you in discussions about your baby's care and treatment?	Number	Percentage
Yes, always	3694	62%
Yes, sometimes	1800	31%
No	408	7%
Total specific responses	5902	100%
Missing responses	52	0%

Answered by all

D4 Were you told about any changes in your baby's condition or care?	Number	Percentage
Yes, always or nearly always	4760	81%
Yes, sometimes	916	16%
No, I was not told about changes	157	3%
Total specific responses	5833	100%
Not sure / can't remember	74	0%
Missing responses	47	0%

Answered by all

©2015 Picker Institute Europe. All Rights Reserved.

D5 When a ward round was taking place, were you allowed to be present when your baby was being discussed?

	Number	Percentage
Yes, always or nearly always	3591	72%
Yes, sometimes	820	16%
No, I was not allowed to be there	626	12%
Total specific responses	5037	100%
Not sure / I did not know about ward rounds	846	0%
Missing responses	71	0%

Answered by all

D6 Where possible, did staff arrange your baby's care (such as weighing, bathing) to fit in with your usual visiting times?

	Number	Percentage
Yes, always or nearly always	2918	64%
Yes, sometimes	1004	21%
No, but I would have liked this	693	15%
Total specific responses	4615	100%
No, but this was not necessary	1243	0%
Missing responses	96	0%

Answered by all

D7 Overall, did staff help you feel confident in caring for your baby?

	Number	Percentage
Yes, definitely	4752	81%
Yes, to some extent	961	16%
No	163	3%
Total specific responses	5876	100%
Missing responses	78	0%

Answered by all

D8 If you wanted to express breast milk for your baby, were you given the support you needed from neonatal staff?

	Number	Percentage
Yes, definitely	3699	78%
Yes, to some extent	777	16%
No	259	5%
Total specific responses	4735	100%
I did not want to express milk	857	0%
I could not express milk	240	0%
Missing responses	122	0%

Answered by all

©2015 Picker Institute Europe. All Rights Reserved.

D9 When you were in the neonatal unit, were you given the feeding equipment you needed for expressing, such as a breast pump and sterilisation equipment?

	Number	Percentage
Yes, definitely	3908	83%
Yes, to some extent	367	8%
No	446	9%
Total specific responses	4721	100%
Missing responses	36	0%

Answered by those who could and wanted to express breast milk

D10 Were you given enough privacy in the neonatal unit for expressing milk and/or breastfeeding your baby?

	Number	Percentage
Yes, definitely	3606	77%
Yes, to some extent	777	17%
No and it bothered me	172	4%
No, but I did not mind	127	3%
Total specific responses	4682	100%
This was not needed	1104	0%
Missing responses	168	0%

Answered by all

D11 If you wanted to breastfeed your baby, were you given enough support to do this from neonatal staff?

	Number	Percentage
Yes, definitely	3454	75%
Yes, to some extent	855	19%
No	293	6%
Total specific responses	4602	100%
I did not want to breastfeed my baby	741	0%
My baby was unable to breastfeed for medical reasons	438	0%
Missing responses	173	0%

Answered by all

D12 If you fed your baby formula milk, were you given enough support to do this from neonatal staff?

	Number	Percentage
Yes, definitely	2677	76%
Yes, to some extent	662	19%
No	168	5%
Total specific responses	3507	100%
I did not want to feed my baby formula milk	2060	0%
Missing responses	387	0%

Answered by all

©2015 Picker Institute Europe. All Rights Reserved.

E. Environment and facilities

E1 Were you given enough privacy when discussing your baby's care on the neonatal unit with staff?	Number	Percentage
Yes, always or nearly always	4026	68%
Yes, sometimes	1166	20%
No	310	5%
Total specific responses	5502	94%
No, but I did not mind	393	6%
Missing responses	59	0%

Answered by all

E2 Was there enough space for you to sit alongside your baby's cot in the unit?	Number	Percentage
Yes, definitely	3989	67%
Yes, to some extent	1628	28%
No	258	4%
Total specific responses	5875	100%
Missing responses	79	0%

Answered by all

E3 In your opinion, was there adequate security on the neonatal unit?	Number	Percentage
Yes, definitely	4708	80%
Yes, to some extent	959	16%
No	227	4%
Total specific responses	5894	100%
Missing responses	60	0%

Answered by all

E4 If you wanted to stay overnight to be close to your baby, did the hospital offer you accommodation?	Number	Percentage
Yes, always or nearly always	2884	63%
Yes, sometimes	636	14%
No	1018	23%
Total specific responses	4538	100%
I did not want/need to stay overnight	1190	0%
Missing responses	226	0%

Answered by all

©2015 Picker Institute Europe. All Rights Reserved.

E5 Were you able to visit your baby on the unit as much as you wanted to? (please only think about unit-related reasons and not personal reasons such as needing to care for other children)	Number	Percentage
Yes, definitely	5393	92%
Yes, to some extent	352	6%
No	95	2%
Total specific responses	5840	100%
I did not visit my baby	26	0%
Missing responses	88	0%

Answered by all

F. Information and support for parents

F1 If you asked questions about your baby's condition and treatment, did you get answers you could understand?	Number	Percentage
Yes, always or nearly always	4682	79%
Yes, sometimes	1081	19%
No, never	135	2%
Total specific responses	5898	100%
I did not ask any questions	17	0%
Missing responses	39	0%

Answered by all

F2 Were you given enough written information to help you understand your baby's condition and treatment?	Number	Percentage
Yes, definitely	2212	42%
Yes, to some extent	1142	22%
No, I was not given enough written information	592	12%
I did not get any written information	1305	25%
Total specific responses	5251	100%
I did not want or need any written information	624	0%
Missing responses	79	0%

Answered by all

F3 Did you have an opportunity to go through your baby's medical notes (not just the nursing notes) with staff while they were in the neonatal unit?	Number	Percentage
Yes	1379	23%
No, but I wanted to	1411	24%
No, but I did not want to	817	14%
I did not know about the medical notes	2249	39%
Total specific responses	5856	100%
Missing responses	98	0%

Answered by all

F4 Were you offered emotional support or counselling services from neonatal unit staff?	Number	Percentage
Yes	1752	30%
No, but I would have liked to have been offered this	1339	23%
I did not need any emotional support or counselling	2782	47%
Total specific responses	5873	100%
Missing responses	81	0%

Answered by all

©2015 Picker Institute Europe. All Rights Reserved.

F5 Were you given enough information about help you could get with expenses related to your baby's stay in the neonatal unit (such as travelling/parking expenses, hardship fund or food vouchers?	Number	Percentage
Yes, definitely	1373	29%
Yes, to some extent	1183	25%
No	2153	46%
Total specific responses	4709	100%
I did not want/need this information	1166	0%
Missing responses	79	0%

Answered by all

F6 Did staff give you any information about parent support groups, such as Bliss or other local groups?	Number	Percentage
Yes, staff gave me this information	1861	36%
No, but this information was available in the unit (e.g. a leaflet in the parents' room)	1749	34%
No, I did not get this information	1543	30%
Total specific responses	5153	100%
Don't know / can't remember	710	0%
Missing responses	91	0%

Answered by all

©2015 Picker Institute Europe. All Rights Reserved.

G. Leaving the neonatal unit

G1 In total, how long did your baby stay in neonatal care (include all the hospitals they stayed in)?

	Number	Percentage
Up to 1 week	2712	47%
More than 1 week but less than 4 weeks	2040	35%
Between 4 weeks and 8 weeks	669	11%
More than 8 weeks, but less than 12 weeks	237	4%
12 weeks or more	187	3%
Total specific responses	5845	100%
Missing responses	109	0%

Answered by all

G2 When your baby was discharged from the neonatal unit, where did they go next?

	Number	Percentage
A hospital maternity ward	1724	30%
Another ward in the hospital	375	7%
Home	3637	61%
Somewhere else	137	2%
Total specific responses	5873	100%
Missing responses	81	0%

Answered by all

G3 Were you offered overnight accommodation with your baby at the hospital before they left the neonatal unit?

	Number	Percentage
Yes	2991	82%
No, but I would have liked it	136	4%
No, but I did not want or need it	502	14%
Total specific responses	3629	100%
Missing responses	46	0%

Answered by those whose baby was discharged home

G4 Did you feel prepared for your baby's discharge from neonatal care?

	Number	Percentage
Yes, definitely	4427	75%
Yes, to some extent	1244	21%
No	232	4%
Total specific responses	5903	100%
Missing responses	51	0%

Answered by all

©2015 Picker Institute Europe. All Rights Reserved.

G5 Were you given enough information on what to expect in terms of your baby's progress and recovery?

	Number	Percentage
Yes, definitely	3658	62%
Yes, to some extent	1668	28%
No	577	10%
Total specific responses	5903	100%
Missing responses	51	0%

Answered by all

G6 How likely are you to recommend this neonatal unit to friends and family, if their baby needed similar care or treatment?

	Number	Percentage
Extremely likely	4452	76%
Likely	1020	18%
Neither likely nor unlikely	241	4%
Unlikely	70	1%
Extremely unlikey	68	1%
Total specific responses	5851	100%
Don't know	56	0%
Missing responses	47	0%

Answered by all

©2015 Picker Institute Europe. All Rights Reserved.

H. You and your baby

H1 Did your baby stay in a neonatal unit in more than one hospital?

	Number	Percentage
Yes	747	13%
No	5201	87%
Total specific responses	5948	100%
Missing responses	52	0%

Answered by all

H2 Did your baby spend most of its time in the neonatal unit named in the letter that came with this questionnaire?

	Number	Percentage
Yes, my baby spent most time in the unit named in the letter that came with this questionnaire	469	62%
No, my baby spent most of its time in another unit	176	23%
My baby stayed a similar amount of time in two or more different units	110	15%
Total specific responses	755	100%
Missing responses	8	0%

Answered by those whose baby stayed in a neonatal unit in more than one hospital

H3 Did you give birth to a single baby, twins or more in your most recent pregnancy?

	Number	Percentage
A single baby	5409	91%
Twins	543	9%
Triplets, quads or more	24	0%
Total specific responses	5976	100%
Missing responses	24	0%

Answered by all

H4 Roughly how many weeks pregnant were you when your baby was born?

	Number	Percentage
Before I was 25 weeks pregnant	57	1%
When I was 25 to 29 weeks pregnant	433	7%
When I was 30 to 32 weeks pregnant	678	11%
When I was 33 to 37 weeks pregnant	2429	41%
When I was 38 weeks pregnant or more	2362	40%
Total specific responses	5959	100%
Missing responses	41	0%

Answered by all

©2015 Picker Institute Europe. All Rights Reserved.

H5 How much did your baby weigh at birth?

	Number	Percentage
Less than 1000g (1kg / 2lb 3oz)	274	5%
1000g to 1500g (1kg to 1.5.kg / 2lb 3oz to 3lb 5oz)	592	10%
1500g to 2500g (1.5kg to 2.5kg / 3lb 5oz to 5lb 8oz)	1888	32%
More than 2500g (2.5kg / 5lb 8oz)	3154	53%
Total specific responses	5908	100%
Missing responses	92	0%

Answered by all

H6 How many babies have you given birth to before this pregnancy?

	Number	Percentage
None	3365	56%
1-2	2217	37%
3 or more	380	6%
Total specific responses	5962	100%
Missing responses	38	0%

Answered by all

H7 Have you previously had a baby admitted to a neonatal unit?

	Number	Percentage
Yes	532	20%
No	2072	80%
Total specific responses	2604	100%
Missing responses	12	0%

Answered by those who had previously given birth to a baby

H8 Who filled in this questionnaire?

	Number	Percentage
The baby's mother	5040	85%
The baby's father	126	2%
Parents together	730	12%
The baby's guardian(s)	7	0%
Total specific responses	5903	100%
Missing responses	97	0%

Answered by all

©2015 Picker Institute Europe. All Rights Reserved.

H9 To which of these ethnic groups would you say the mother of the baby belongs?

	Number	Percentage
White	4832	81%
Mixed	123	2%
Asian or Asian British	632	11%
Black or Black British	257	4%
Chinese or Other Ethnic Group	154	3%
Total specific responses	5998	100%
Missing	2	0%

Answered by all

H10 Do you have a long-standing physical or mental health problem or disability?

	Number	Percentage
Yes	318	5%
No	5604	94%
I'd rather not say	50	1%
Total specific responses	5972	100%
Missing responses	28	0%

Answered by all

H11 Does this problem or disability affect your day-to-day activities?

	Number	Percentage
Yes, definitely	83	26%
Yes, to some extent	138	43%
No	102	32%
Total specific responses	323	100%
Missing responses	2	0%

Answered by those who have a long-standing physical or mental health problem or disability

Mother's age group from response or sample if missing

	Number	Percentage
16-18	12	0%
19-24	591	10%
25-29	1366	23%
30-34	2030	34%
35 and over	1996	33%
Total specific responses	5995	100%
Missing data	5	0%

Answered by all

©2015 Picker Institute Europe. All Rights Reserved.

Picker Institute Europe
Buxton Court
3 West Way
Oxford OX2 0JB

Tel: + 44 (0) 1865 208100
Fax: + 44 (0) 1865 208101

info@pickereurope.ac.uk
www.pickereurope.org

Charity registered in England and Wales: 1081688
Charity registered in Scotland: SC045048
Company limited by guarantee registered in England and Wales

picker
Institute Europe
