# Supplementary material for: Better Use of Data to improve parent Satisfaction (BUDS): protocol for a prospective before-and-after pilot study employing mixed methods to improve parent experience of neonatal care
Source: BMJ Paediatr Open. 2019 Jun 25;3(1):e000515. doi: 10.1136/bmjpo-2019-000515 (PMC6598559; doi:10.1136/bmjpo-2019-000515)
Supplement: Supplementary data [file bmjpo-2019-000515supp001.pdf]

BUDS Parent Interview Question Guide
v1.1/230418/IRAS ID:232205

Title of Project: Better Use of Data to improve parent Satisfaction (BUDS)

Project aims to explore:

- Parent experience of receiving information updates about their baby in neonatal care
- Parent experience of feeling involved in their baby's care

---

1. Introduction (~5 min)

- Introduce self
  - Explain nature and purpose of project
  - Who research is for
- Talk through key points:
  - Interview length 15-30 minutes
  - Interview like a discussion, although specific topics to cover
  - No right or wrong answers
  - Participation is voluntary and right to withdraw
- Introduce audio recorder (so can actively listen to parent and for accuracy)
- Stress confidentiality and anonymity, secure transfer and storage of data , how Findings will be reported
- Check for parent understanding of information sheet and check consent form signed
- Any questions they may have

2. Background (~5 min)

- Check Parent Demographic Form completed
- Their baby's sex, gestation at birth, current age, length of stay on unit, other children (ages), marital status, occupation

3. Experience of receiving information updates in neonatal care (~10-15min)

Aim to obtain an overview of how parents are given information about their baby in neonatal care

- How informed do they feel about their baby every day
- What type of information are they given about their baby, where in the unit and when during their baby's admission
- In what way are they given information (eg verbal, written, phone), what do they prefer
- Who gives them information (eg doctors, nurses)
- How do they feel about the way information is communicated to them
- How often do they request face-to- face updates from staff / how satisfied are they with them / why
- How often do they phone the unit for updates / how satisfied are they with telephone updates/ why

4. Experience of feeling involved in their baby's care (~5-10min)

- How involved do they feel in their baby's care /why / why not
- How sufficient do they feel the information provided about care involvement is

5. Close

- Reassure them about confidentiality and anonymity
- Ask if they would like to be informed of the outcomes of the research (take email address if that is their preferred way of being informed)
