# Supplementary material for: Better Use of Data to improve parent Satisfaction (BUDS): protocol for a prospective before-and-after pilot study employing mixed methods to improve parent experience of neonatal care
Source: BMJ Paediatr Open. 2019 Jun 25;3(1):e000515. doi: 10.1136/bmjpo-2019-000515 (PMC6598559; doi:10.1136/bmjpo-2019-000515)
Supplement: Supplementary data [file bmjpo-2019-000515supp002.pdf]

BUDS Staff Interview Question Guide
v1.1/230418/IRAS ID:232205

Title of Project: Better Use of Data to improve parent Satisfaction (BUDS)

Project aims to explore:
- Neonatal staff experience of giving parents information updates about their baby in neonatal care
- Neonatal staff perceptions of how updating parents affects their workload

---

1. **Introduction (2 min)**
    - Introduce self
        - Explain nature and purpose of project
        - Who research is for
    - Talk through key points:
        - Interview length 10 minutes
        - Interview like a discussion, although specific topics to cover
        - No right or wrong answers
        - Participation is voluntary and right to withdraw
    - Introduce audio recorder (so can actively listen to staff member and for accuracy)
    - Stress confidentiality and anonymity, secure transfer and storage of data , how findings will be reported
    - Check for understanding of information sheet and check consent form signed
    - Any questions they may have

2. **Background (1 min)**
    - Job role, grade, length of time worked on the unit

3. **Experience of giving parents information updates about their baby in neonatal care (5 min)**
    - How do they give parents information about their baby on the neonatal unit (eg verbal, written, phone)
    - Do they volunteer information to parents or do parents ask for it
    - How often do they offer face-to-face updates to parents / how often do parents ask for them
    - How often do they update parents over the phone

4. **Neonatal staff perceptions of how updating parents affects their workload (5 min)**
    - How much of their job role / regular shift pattern involves updating parents
    - How does updating parents impact on their other clinical duties

5. **Close**
    - Reassure them about confidentiality and anonymity
